# Recent applications of deep learning and machine intelligence on *in silico* drug discovery: methods, tools and databases

**DOI:** 10.1093/bib/bby061

**Published:** 2018-07-31

**Authors:** Ahmet Sureyya Rifaioglu, Heval Atas, Maria Jesus Martin, Rengul Cetin-Atalay, Volkan Atalay, Tunca Doğan

**Affiliations:** 1 Department of Computer Engineering, Middle East Technical University, Ankara, Turkey; 1a Department of Computer Engineering, İskenderun Technical University, Hatay, Turkey; 2 Cancer System Biology Laboratory (CanSyL), Graduate School of Informatics, Middle East Technical University, Ankara, Turkey; 3 European Molecular Biology Laboratory, European Bioinformatics Institute (EMBL–EBI), Cambridge, Hinxton, UK; 4 Cancer System Biology Laboratory (CanSyL), Graduate School of Informatics, Middle East Technical University, Ankara, Turkey and European Molecular Biology Laboratory, European Bioinformatics Institute (EMBL–EBI), Cambridge, Hinxton, UK

**Keywords:** virtual screening, drug-target interactions, ligand-based VS and proteochemometric modelling, machine learning, deep learning, compound and bioactivity databases, gold-standard data sets

## Abstract

The identification of interactions between drugs/compounds and their targets is crucial for the development of new drugs. *In vitro* screening experiments (i.e. bioassays) are frequently used for this purpose; however, experimental approaches are insufficient to explore novel drug-target interactions, mainly because of feasibility problems, as they are labour intensive, costly and time consuming. A computational field known as ‘virtual screening’ (VS) has emerged in the past decades to aid experimental drug discovery studies by statistically estimating unknown bio-interactions between compounds and biological targets. These methods use the physico-chemical and structural properties of compounds and/or target proteins along with the experimentally verified bio-interaction information to generate predictive models. Lately, sophisticated machine learning techniques are applied in VS to elevate the predictive performance.

The objective of this study is to examine and discuss the recent applications of machine learning techniques in VS, including deep learning, which became highly popular after giving rise to epochal developments in the fields of computer vision and natural language processing. The past 3 years have witnessed an unprecedented amount of research studies considering the application of deep learning in biomedicine, including computational drug discovery. In this review, we first describe the main instruments of VS methods, including compound and protein features (i.e. representations and descriptors), frequently used libraries and toolkits for VS, bioactivity databases and gold-standard data sets for system training and benchmarking. We subsequently review recent VS studies with a strong emphasis on deep learning applications. Finally, we discuss the present state of the field, including the current challenges and suggest future directions. We believe that this survey will provide insight to the researchers working in the field of computational drug discovery in terms of comprehending and developing novel bio-prediction methods.

## Introduction

The development of new drugs remains the key problem and challenge to improve the current field of biomedicine. Computational methods have been used in bioinformatics and cheminformatics studies for nearly three decades, to aid understanding the molecular mechanisms and propose novel treatment options for several diseases. Recent advances in computational power (e.g. massively parallel and computing on graphical processing units (GPU)) and in data analysis and inference techniques (e.g. artificial intelligence, machine learning and deep learning) provide opportunities for various fields of data science, including biomedicine.

In this study, our objective is to provide an overview of recent applications of computational drug discovery methods, called virtual screening (VS), where the aim is to predict the bio-interactions between drug-like small molecules (i.e. compounds) and potential target proteins for the identification of novel drugs, using structural and physico-chemical properties of compounds and targets along with the experimentally known (i.e. validated) bioactivities. In this review, we explored various data resources that provide vast amount of information, which is essential for conducting VS studies. We also investigated novel machine learning approaches with recent applications to drug-target interaction (DTI) prediction. In this framework, we discussed in detail the recent applications of deep learning techniques, which outperformed state-of-the-art VS methods. Finally, we stated our observations and comments about the current status of the field of VS.

We divided the text in six main chapters. The first chapter, introduction, defines the basic terminology, provide statistics regarding the relevant information stored in source biological databases, summarizes the experimental procedures along with computational approaches in drug discovery. The second chapter, descriptors and features for VS, lists and explains in detail molecular representations and descriptors for both compounds and targets. The third chapter, libraries and toolkits for VS, expresses the available computational tools and libraries to generate these descriptors/representations. The fourth chapter, compound and bioactivity databases and gold-standard data sets, explains the available repositories for bioactivity data. The fifth chapter, machine learning approaches in VS, provides an overview of the recent machine learning and data mining applications, including the deep learning for drug discovery, together with the explanations of performance evaluation metrics and a predictive performance comparison between the machine learning-based VS methods. The sixth and the last chapter, discussion and conclusion, summarizes the field and briefly discusses the future directions together with challenges.

The terminology used in this survey is given below:

- A ligand is a molecular structure that physically binds another molecular structure and modulates its function.
- A compound is a chemical structure that is formed by the combination of two or more atoms that are connected by chemical bonds.
- Some of the compounds, bioactive compounds, modulate the functions of bio-molecules such as proteins.
- A drug is an approved [by Food and Drug Administration (FDA), for example] bioactive compound that acts on protein targets to cure/decelerate a specific disease or to promote the health of a living being.
- A target protein (or just a target) is a naturally occurring bio-molecule of an organism that is bound by a ligand and has its function modulated, which results in a physiological change in the body of the organism.
- The Anatomical Therapeutic Chemical (ATC) Classification System is a controlled vocabulary to classify drugs hierarchically based on their therapeutic, pharmacological and chemical properties. There are five levels in each ATC code and each level of an ATC code represents a different property of drugs. The first level represents anatomical groups; the second level shows a therapeutic main group; the third level represents a therapeutic and pharmacological subgroup; the fourth level represents a chemical, therapeutic and pharmacological subgroup; and the fifth level shows the indicated chemical substance.
- Cheminformatics is the application of computational techniques to the field of chemistry. Most of the VS methods are considered to be cheminformatics based.

It is important to note that, in this article, the terms: ‘small molecule’ and ‘compound’ are used synonymously to refer to the ‘chemical substances’. The term ‘bioactive compound’ corresponds to chemical substances with biological activities. The term ‘ligand’ represents a chemical substance that interacts with a target biomolecule to accomplish a biological purpose. The term ‘drug’ is used to represent approved bioactive compounds, which are currently being used in the clinics. ‘Active pharmaceutical ingredients’ (APIs) refers to the biologically active ingredient in a drug and is responsible for the interactions with cellular polymeric macromolecules as well as small secondary messenger molecules. The terms ‘biomolecule’, ‘receptor’, ‘target’ and ‘protein’ refer to the cellular biological molecules targeted by APIs and/or bioactive compounds.

**Figure 1.**
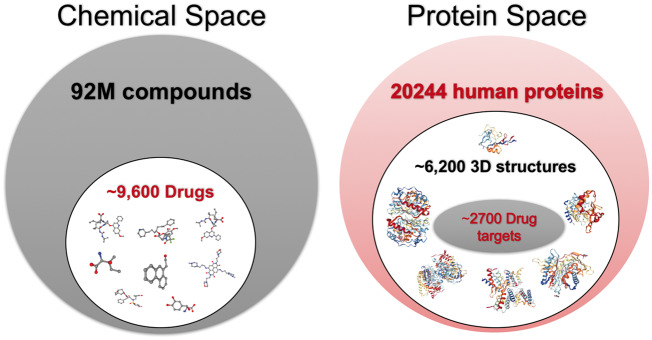
Statistics of current chemical and protein spaces in open access chemical and biological data repositories.

In terms of the statistics, there are tens of millions of compounds available in compound and bioactivity databases [1–4]. There are about 9000 FDA-approved small molecule drugs (approved + experimental) [5], roughly 550 000 reviewed protein records available (20 244 of which are human proteins) in protein sequence and annotations resources (e.g. UniProtKB/Swiss-Prot) and nearly 2700 of human proteins are known to be targeted by either approved or experimental drugs [1, 6]. The 3D structure information of proteins and compounds provide important qualities of these molecules to determine their functions and bioactivities. However, 3D structures of a relatively small subset of compounds (i.e. around 24 000) and human proteins (i.e. about 6200) are experimentally known (partly or completely) and currently available in Protein Data Bank–PDB (Figure 1) [5].

The main role of drugs, which are bioactive compounds, is the alteration of cellular events involved in disease conditions for treatment purposes. The following two problems are of importance for the hit discovery, one of the initial steps in the development of new drugs:

- Identification of novel bioactive compounds for a target protein; and
- identification of new targets for known bioactive compounds.

**Figure 2.**
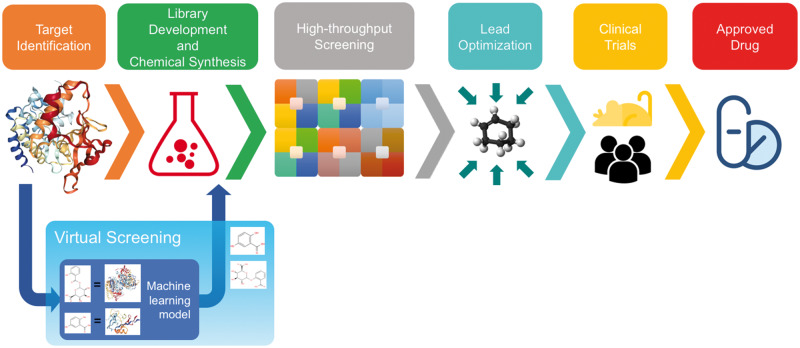
A broad overview of drug development and the place of virtual screening in this process.

Drug discovery is defined as the process of identifying the roles of bioactive compounds to develop new drugs, and it is usually one of the initial steps in a drug development pipeline. Traditionally, drug research and development starts with the identification of the biomolecular targets for an intended treatment and proceeds with the high-throughput screening experiments to identify bioactive compounds for the defined targets, together with the corresponding bioactivity levels. The aim of high-throughput screening is to find suitable drug candidates. With the advancement of high-throughput screening technology, it is now possible to conduct experiments to scan thousands of different compounds and detect their bioactivity levels on selected target proteins [7]. However, designing high-throughput screening experiments is expensive, it is a time-consuming process, and it requires advanced laboratories having chemical and biological libraries. Furthermore, it is not feasible to conduct high-throughput screening experiments for all expressed proteins in the human genome and for all known compounds [8]. Another problem with high-throughput screening is its high failure rates, which limits the identification of novel drugs [9]. The problem escalates when we consider the process of drug development. The term drug development refers to the whole process to bring a drug to the market, starting with the drug discovery and ending with clinical trial phases. In Figure 2, main phases of the drug development procedure are shown. Most of the drug candidates fail to become an approved drug in the late phases of clinical trials because of the unexpected side effects and toxicity problems. In 2010, the cost of developing a single drug was estimated about 1.8 billion US dollars, and the process requires about 13 years the on average [8].

To address the abovementioned challenges and problems, computational methods have been developed and used in the past decades. The field of *in silico* estimation of unknown drug-target pairs using statistical models is called ‘virtual screening’–VS–(i.e. DTI prediction). In drug development pipelines, VS methods are mostly placed just before the high-throughput screening, so that the unlikely drug-target pairs are eliminated; as a result, only potentially active combinations are run through the experimental screening procedure (Figure 2). In this sense, VS has the potential to greatly reduce the cost and time required for high-throughput screening [10]. Although the main purpose of VS is to identify new drug candidates for specified targets, it also has other applications such as finding beneficial drug pairs [11] and the prediction of ATC codes for known drugs [12, 13]. In addition, the computational approaches mainly employed in VS can also be used for drug repurposing and off target effect identification, where the aim is to find new uses for the already approved drugs [14]. Drug repurposing is an important research area since the approved drugs are already tested for safety issues; therefore, the cost and the required time for marketing repurposed drugs is much less than discovering and marketing novel drugs [15]. There are various examples of repurposed drugs in the market, most of which are being used for treatments of multiple diseases [16].

There have been several successful applications of VS in detecting compounds with high affinities against pre-specified targets [17]. Some of these drug candidate compounds have also passed the clinical trials and became marketed drugs [18–22]. Doman *et al.* showed that their VS approach substantially improved the rate of identified drug candidates against protein tyrosine phosphatase-1B enzyme. The authors experimentally showed that the hit rate of their method was 34.8%, whereas the hit rate of the high-throughput screening experiment was only 0.021% [23]. Another successful application of VS was proposed by Powers *et al.*, which led to the discovery of a novel inhibitor of AmpC ß-lactamase [24].

**Figure 3.**
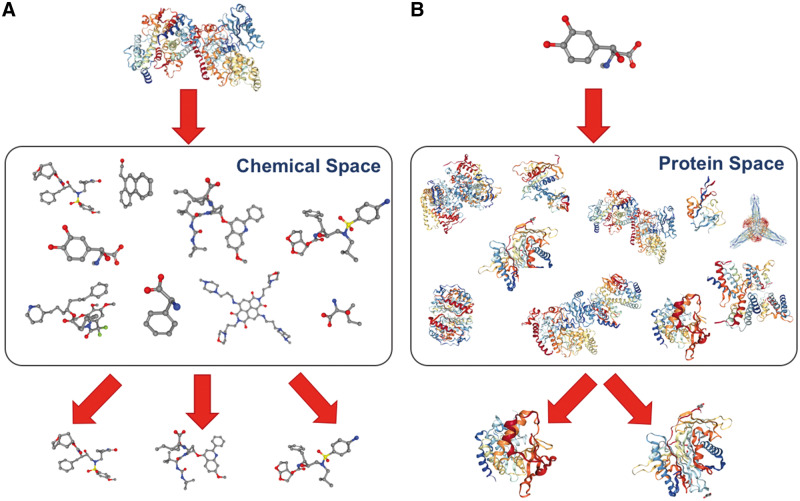
(**A**) In conventional virtual screening, multiple compounds are screened against a pre-specified target, and candidate interacting compounds (i.e. ligands) are identified, whereas (**B**) in target prediction (i.e. reverse virtual screening), a compound is searched against multiple proteins and candidate targets are identified.

Both in high-throughput screening experiments and in conventional VS approaches, the aim is to identify whether a given set of compounds is bound to a pre-specified target protein or not. In these applications, off-target effects are generally overlooked and other possible targets of the compounds cannot be identified. However, it is known that most of the bioactive compounds act on multiple targets (which causes these off target effects); in fact, the cases where a compound interacts with only a one-target protein are considered as exceptional [25, 26]. The identification of the off-target effects is crucial to obtain potential side effect and toxicity information of the test compounds. For this purpose, another type of computational approach, target prediction (also known as the reverse VS), was proposed [27, 28]. In target prediction, a compound is screened against a large set of proteins with the aim of identifying all possible targets of the corresponding compound (Figure 3). Generally speaking, the goal of both approaches is the prediction of unknown interactions between various compound–protein pairs.

Most of the VS methods make use of biological, topological and physico-chemical properties of compounds and/or targets along with the experimentally validated bioactivity values of compound-target pairs to predict the unknown activities [29, 30]. For this, it is required to computationally record the compounds and targets as quantitative vectors (i.e. representations and descriptors) according to their molecular features. VS methods use these feature vectors as input to model the interactions between compounds and target molecules. VS methods can be divided into three groups based on the employed input features:

- Structure-based VS employs 3D structure of targets and compounds to model the interactions [31, 32],
- Ligand-based VS uses the molecular properties of compounds (mostly non-structural) to model the interactions with targets [29, 33, 34],
- Proteochemometric modeling (PCM) approach models the interactions by combining non-structural descriptors of both compounds and targets at the input level [35–38].

Previously, VS was mainly divided in two groups (i.e. structure-based and ligand-based methods) [39, 40]; however, recent advances in PCM have put this field forward to be considered as a third group [37]. Both ligand-based and PCM methods can be considered as non-structure-based VS methods. The field of ligand-based VS has been extensively reviewed by Geppert *et al.* and Lavecchia and Di Giovanni [33, 34]. In another study, Glaab reviewed the recent developments in both ligand- and structure-based VS approaches. The author defined a comprehensive pipeline for VS over a target protein of interest and overviewed workflow management systems. The whole process was divided into four main steps, namely, data collection, pre-processing, screening, selectivity and ADMETox (i.e. absorption, distribution, metabolism, excretion and toxicity) filtering, and explained each step with a focus on relevant open-access software and databases. The author also implemented a downloadable cross-platform software by integrating open-access screening tools using the Docker platform [41]. Qiu *et al.* introduced the emergence of PCM and mentioned its advantages by referring to studies in which PCM models outperform conventional quantitative structure-activity relationship (QSAR) models in DTI modelling. The authors focused on the recent progress in PCM modelling in terms of target descriptors, cross-term descriptors and application scope of PCM, including protein-small molecule and protein-macro molecule interactions. The authors reported that, with further advancements in molecular representations, machine learning techniques and the available bioactivity data, it may be possible to generate PCM models for more complicated systems such as ligand-catalyst-target reactions, which could provide help to identify biochemical reactions more accurately [37]. The field of PCM was also reviewed by van Westen *et al.* and Cortés-Ciriano *et al.* [36, 38].

Structure-based VS methods can only be applied when the 3D structure of both targets and the candidate compounds are available, which are either experimentally determined by X-ray crystallography or Nuclear magnetic resonance (NMR), or predicted by computational approaches such as the homology modelling. Once the 3D structural information is obtained, docking can be applied to find interactions between a compound and a target, which predicts compound conformations in the binding site of the target using search algorithms and ranks them via scoring functions representing estimated binding affinities [23, 27]. Some of the most commonly used docking tools are AutoDock [42], DOCK [43], Glide [44], GOLD [45], FlexX [46] and Fred [47]. These methods rely on the conformations of atoms in 3D space; as a result, they are computationally intensive since the number of possible conformations of proteins and compounds increase exponentially with the increasing number of rotatable bonds. Moreover, the calculation of binding energies is a problematic issue [17]. In addition to these traditional methods, there are also similarity-based docking approaches such as HomDock [48], *e*SimDock [49] and *fkcombu* [50] that use structural similarities of compounds to predict their protein-bound states by aligning them on the experimentally determined 3D structure of a reference compound that is in complex with a target protein or evolutionarily related structures of that target protein [49]. Therefore, they do not require searching for low energy conformations of compounds contrary to conventional methods, which reduces the computational cost and makes them faster than traditional docking methods [48]. Both approaches can achieve high performance in estimating the interactions; however, their applicability is limited since the structural information is not available for the majority of the proteins and compounds, and the experimental identification of the 3D structures is challenging [8]. Although homology models of proteins can be used as templates for docking, it is not possible to obtain a reliable model for all proteins because of the lack of a reference protein structure that is evolutionarily close to the target protein to be modelled. Even if similarity-based docking approaches are less sensitive to weakly homologous protein models [49], they are not feasible in the absence of similar compounds to the reference compound. Therefore, non-structure-based VS methods are more preferable if a reliable target structure is not available [51]. It was reported in the literature that the non-structure-based methods have a similar potential to detect drug targets as the structure-based methods [52]. In addition, several studies showed that structure and non-structure-based methods often provide complementary results [28, 53–55]. There are also hybrid-type methods that combine 3D structure information along with the ligand-based information in the literature [51]. Structure-based VS methods are out of the scope of this study, and information about this field can be obtained from the literature [31, 32, 52, 56, 57].

## Descriptors and features for VS

Compounds and biological target molecules are required to be quantized to be used in VS models. Molecular representations and descriptors are employed for this purpose. A descriptor should reflect the intrinsic physical and chemical properties of the corresponding molecule, so that the statistical model can learn and generalize the shared properties among the molecules that lead to the interaction between compounds and targets. After the models are constructed using the descriptors of known ligand-receptor pairs, interaction predictions are produced for the unknown ligand-receptor couples, by providing query descriptors as input to the model.

There are various types of descriptors both for small molecule compounds and target proteins, each have strengths and weaknesses in terms of the power of representation of molecular properties. The descriptors, which are highly used in the literature, are explained in this chapter, which is further divided into two subsections: compound and target descriptors. In the first subsection, we first describe the line representations that are used to store and search compounds in data repositories. Subsequently, several types of numerical descriptors for compounds are explained. Finally, target descriptors are investigated.

### Compound descriptors

Line notations have been proposed to express the 2D structures of compounds as a string of characters [58–60], to be able to computationally store and search them in chemical databases. Line notations are also used by cheminformatics libraries and toolkits to generate molecular descriptors. Each line notation uses a distinct algorithm to represent structures and chemical properties (i.e. atoms, bonds and aromaticity) of compounds. The most popular line notations are SMILES [58] and InChI [59] notations (for detailed information, please refer to the supplementary material). Graphical representations are drawings of compounds to display the positions of its atoms and bonds in 2D- or 3D. Most chemical databases (e.g. PubChem and ChEMBL) provide both line and graphical representations for the recorded compounds. Table 1 includes example graph and line notation representations for a sample compound.

**Table 1.** Chemical formula, 2D/3D graphical representation, SMILES and InChI notations of aspirin

Category	Representation
**Compound name**	Aspirin	
**Chemical formula**	C_9_H_8_O_4_
**3D/2D structure**	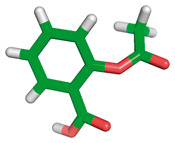	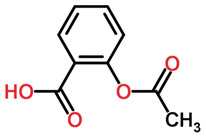
**SMILES**	CC(=O)OC1 = CC=CC=C1C(=O)O	
**InChI**	InChI = 1S/C9H8O4/c1-6(10)13-8-5-3-2-4-7 (8)9(11)12/h2-5H, 1H3, (H, 11, 12)	

Molecular descriptors are representative numerical vectors (i.e. feature vectors) for compounds that are generated by algorithms based on the geometrical, structural and physiochemical properties. There are more than a thousand different types of molecular descriptors in the literature [61]. Molecular descriptors are categorized based on the dimensionality of the included information. A popular sub-group of molecular descriptors are fingerprints (i.e. binary vectors), where each dimension of the vector represents presence (1) or absence (0) of a particular property. Fingerprints are used to represent compounds by their chemical bonds, structural fragments, functional groups and connectivity pathways. Several studies have been performed to investigate the effects of choice of fingerprints on prediction performance in VS [62–66]. These studies showed that each fingerprint type represents different aspects of compounds; therefore, selection of fingerprints is crucial for VS [62]. Sawada *et al.* trained several models using 18 different compound descriptors to compare prediction performance of these descriptors [67]. They showed that KEGG chemical function and substructures (KCF-S) fingerprints performed best among 18 different individual fingerprints based on multiple criteria. However, the dimensionality of the KCF-S vector is considerably higher compared with the conventional compound fingerprints [i.e. 63 891 as opposed to 1024 for extended connectivity fingerprints (ECFP4)], which significantly increases the computational complexity, and it is debatable if the obtained performance increase worth the significant increase in computational requirements. The authors also showed that integrating multiple descriptors usually improve the predictive performance; nevertheless, the performance gain was not significant in most cases. In another study, Cano *et al.* used several compound descriptors with random forest algorithm for automatic selection and ranking of molecular descriptors based on relevancy [68]. Their report indicated that automatically selected and combined features significantly enhanced the prediction accuracy. Duan *et al.* reported that no fingerprint method could outperform the others considering all targets and that different types of fingerprints are effective on different targets [65]. Bender *et al.* compared 37 different fingerprints that belong to four classes of molecular descriptors (i.e. circular fingerprints, circular fingerprints considering counts, path-based and keyed fingerprints and pharmacophoric descriptors) [69]. They reported that different fingerprints retrieved different active compounds, and combination of multiple fingerprints provided the best performance. Their evaluation results showed that ECFP4 performed best, when the fingerprints were evaluated individually. Soufan *et al.* created several types of compound features and used a wrapper method (please see supplementary material for details regarding feature selection methods) to create the most representative features for training [70, 71]. The authors showed that combining several features with a classifier performance aware system enhanced the prediction results.

To sum up, it can be said that each conventional molecular descriptor is capable of representing different properties of compounds. For example, substructure keys-based fingerprints are created based on the presence or absence of predefined substructures in compounds (e.g. MACCs [72]); circular fingerprints can be used to represent structural properties of compounds, independent of a pre-defined key set (e.g. ECFPs [73]). On the other hand, pharmacophore descriptors can represent complex physico-chemical properties of compounds. Therefore, combining different molecular descriptors is frequently preferred in the literature. Compound descriptors and their properties are listed in Table 2. Citations column in this table references the studies from the literature that used the corresponding descriptors in their methods.

**Table 2.** Compound descriptors: categories, properties and fingerprints

Descriptor category	Properties	Fingerprints	Citations
0D descriptors	Molecular weight Atom number Atom-type count Other basic descriptors such as number of heavy atoms		[76]

1D descriptors	Functional groups List of structural fragments Substituent atoms		[61]

2D descriptors	Topological descriptors Graph invariants Graph-based substructures Connectivity bonds	Substructure keys based (e.g. MACCS) Path based (e.g. DayLight and FP2) Circular (e.g. ECFPs)	[65, 67, 73]

3D descriptors	Steric properties Geometrical molecular descriptors Surface area Volume Binding site properties 3D-based graph invariants	Geometrical (e.g. triangular descriptors) Pharmacophore (e.g. hydrogen bond, hydrophobicity, charge and aromacity)	[61, 77]

Non-structure-based molecular descriptors	Substring occurrence in SMILES Text-based molecular fingerprints ATC code annotations	LINGO descriptors	[78, 79]

Calculation of pairwise similarities of compounds based on fingerprints is another important issue in VS. Various types of measures have been proposed for this purpose such as the Dice coefficient [74] or the Tanimoto coefficient, which currently is the most popular similarity measure for compounds [62, 75]. Bajusz *et al.* performed statistical analysis and ranking of eight different similarity metrics using the sum of ranking differences and the analysis of variance methods [62]. They used ECFP4 and Chemaxon Chemical Fingerprints to represent compounds. The authors first showed that all the similarity metrics had significantly better performance compared with the random selection. They reported that Cosine, Dice, Tanimoto and Soergel metrics performed better than the others. For more details about fingerprints and similarity measures, please refer to the first section in the supplementary material.

### Target protein descriptors

In proteochemometrics, both ligand and target spaces are modelled to accurately predict DTIs in a large scale. Hence, target protein descriptors are employed along with compound molecular descriptors in PCM [35, 38]. Considering the type of protein properties used for the feature generation, target descriptors are mainly categorized as sequence- and structure-based descriptors. While sequence-based target descriptors use the amino acid sequence of proteins, which can be retrieved from UniProt Knowledgebase (http://www.uniprot.org) [80], structure-based descriptors use 3D atomic coordinates of proteins retrieved from Protein Databank–PDB– (http://www.rcsb.org) [81]. In terms of the properties they describe, target descriptors can roughly be divided into six groups, as briefly explained below and shown in Table 3. Citations column in this table references the studies from the literature, which employed the corresponding descriptors in their methods.

**Table 3.** Categories of target descriptors based on the properties they describe

Descriptor category	Descriptor type	Citations
Sequence composition	Amino acid composition (AAC) Dipeptide composition (DC) Tripeptide composition (TC)	[82–85]

Physico-chemical properties	Autocorrelation (normalized Moreau-Broto, Moran and Geary) Composition, transition and distribution (CTD) Conjoint triad (CTriad) Sequence-order-coupling number (SOCN) Quasi-sequence-order descriptors (QSO) Pseudo amino acid composition (Pse-AAC) Amphiphilic pseudo amino acid composition (Am-Pse-AAC) Z-scales MSWHIM Vectors of hydrophobic, steric and electronic properties (VHSE) FASGAI ProtFP	[36, 82–84, 86]

Similarity measures	Sequence based: BLOSUM/PAM substitution matrix Needleman–Wunsch Score Normalized Smith–Waterman Score Position-specific scoring matrix (PSSM) Substitution matrix representation (SMR) Structure based: Global alignment scores RMSD score GDT score MaxSub score TM ccore Local alignment scores GA score Match score eMatchSite score PS score Ontological annotation semantic similarity	[36, 86–91]

Topological properties	T-scales ST-scales	[36, 84]

Geometrical characteristics	Residue–residue contacts Local descriptors of protein structure (LDPS) Bond lengths, bond angles and dihedral angles Secondary structure prediction B-factor and disordered residues Solvent accessible surface area	[86, 88, 92, 93]

Functional sites	Protein domain profiles Binding pockets and cavities FuzCav FLAP	[83, 84, 94, 95]

Descriptors based on sequence composition reflect the occurrence frequencies of different amino acid combinations on a protein sequence [96]. Descriptors based on physico-chemical properties describe protein sequences in terms of a combination of physical and chemical properties of amino acids such as hydrophobicity, van der Waals volume, polarity, polarizability, charge, secondary structure and solvent accessibility [36, 82, 96–109]. Descriptors based on similarity measures use similarities between proteins via sequence or structural alignments, based on the idea that similar targets may interact with similar compounds [110–118]. Descriptors based on topological properties characterize amino acids according to atom-connectivity indices generated from molecular graphs [119, 120]. Descriptors based on geometrical characteristics reflect structural characteristics of proteins related to shape, size, atomic positions in space, etc., mainly including residue–residue contacts, bond lengths, bond angles and torsion angles between atoms of residues, secondary structures, flexibility and solvent accessibility of proteins [92, 121–124]. Descriptors based on functional sites describe certain functional characteristics of proteins that can be responsible for the interactions with other molecules such as proteins, small molecules and nucleic acids [38, 94, 95, 125, 126]. For detailed information about different types of target descriptors, please refer to the supplementary material.

The selection of descriptor sets is important to be able to generate high-performance predictive models using PCM. There are a few studies on benchmarking of target descriptors. In 2007, Ong *et al.* evaluated the effectiveness of 10 commonly used descriptor sets (i.e. amino acid composition (AAC); dipeptide composition (DC); three types of autocorrelation; Composition, transition and distribution (CTD); Quasi-sequence-order descriptors (QSO); Pse-AAC; combination of AAC and DC; and combination of the first eight descriptors) for the prediction of protein functional families using support vector machines. The authors reported that the selected descriptors were effective in general, and their performance did not significantly differ from each other although combined sets of descriptors provided better results [82]. In another study, van Westen and colleagues [36] compared the performances of 13 different types of amino acid descriptors (i.e. three variants of z-scales, BLOSUM, FASGAI, MSWHIM, T-scales, ST-scales, VHSE and four variants of ProtFP as a novel descriptor set) and their combined versions, for bioactivity modelling using random forest classifiers. According to their findings, z-scale descriptors and combined sets were consistently better than the others while ProtFP and ST-scales descriptors consistently performed worse. Furthermore, they showed that the generated PCM models outperformed QSAR model that uses only compound descriptors. Shaikh *et al.* also developed PCM models for the prediction of DTIs using sequence- and structure-based descriptors, employing different machine learning techniques. The authors reported that, while models generated using random forests and support vector machines outperformed the others, there was no significant difference between the two types of descriptor sets in terms of the model performance. As a result, the authors stated that using sequence-based descriptors was more advantageous as it comprised a larger set of proteins [83]. Apart from these studies, Sun *et al.* performed an analysis for the prediction of RNA-binding protein residues using the random forest algorithm. They developed different predictive models based on five types of protein features, including similarity measures, geometrical characteristics and physico-chemical properties of amino acids as well as two newly developed structural features. Among all models generated using these features separately, and in different combinations, they found that the model with the highest performance was the one combining all these five features [86]. Based on these studies, it can be inferred that there is no outstanding descriptor type that represents the proteins to achieve a significantly higher predictive performance. Therefore, we suggest researchers to select protein descriptors specific to the problem at hand by carrying out performance comparison tests. Also, combinations of different protein features should be considered in these tests to be able to capture distinct aspects of proteins in one model.

## Libraries and toolkits for VS

One issue in VS field is finding a convenient resource (i.e. a computational tool or a programming library) to accomplish specific tasks such as the construction of molecular descriptors, interconversion between two different representations, calculation of pairwise molecular similarities or the applications of various statistical and machine learning algorithms for DTI prediction. Several libraries and toolkits have been developed for these purposes, each supported by different operating system(s) and programming language(s). In this chapter, we describe these libraries and toolkits.

Table 4 provides information on tools, their features and computational dependencies for compounds. Further information about compound-specific toolkits and libraries can be found in the supplementary material. Target descriptors are valuable sources to be used in predictive models not only for DTI prediction but also for protein structure and function prediction and estimation of protein-protein interactions. To facilitate the retrieval of protein data and calculation of protein features, a vast number tools and data services have been constructed. Some of currently available tools and libraries are shown in Table 5 and explained in detail in the supplementary material.

**Table 4.** Libraries and toolkits for cheminformatics

Tools and libraries	Basic properties and included descriptors	Operating systems	Programming languages
RDKit [127]	Descriptor and fingerprint generation for machine learning; molecular database cartridge for PostgreSQL; supporting substructure and similarity searches as well as various descriptor calculators; automatic feature perception (i.e. rings, bonds, hybridization and aromaticity) Supported file formats: SMILES, SMARTS and InChI	Microsoft Windows, Linux, Mac OSX	Python; wrappers are available for Java and C#

OpenBabel [128]	Filtering and searching molecular files; converting files, molecular searching, chirality detection and superimposing molecules; Gasteiger–Marsili partial charge calculation; support for molecular mechanics; hydrogen addition and deletion; isotope support, calculation of average and exact masses; automatic feature perception (rings, bonds, hybridization and aromaticity) Supported file formats: mol2, PDB and SMILES	Microsoft Windows, Linux, Mac OSX	C++, Perl, Python interfaces

Dragon [129]	Calculation of molecular descriptors; graphical interface for selection of structures; providing graphics and statistics tools; preliminary descriptor analysis such as the analysis of molecule distribution in the descriptor space, as well as a preliminary correlation analysis; a molecule viewer to display the molecular structures; principal component analysis implementation for the selected sets of descriptors Supported file formats: SMILES, MDL, Sybyl	Microsoft Windows, Linux	Stand-alone application

DayLight Tookit [130]	Subgraph pattern matching; analyzing and manipulate 2D and 3D data; creating new fingerprints; specifying size and folding parameters for a fingerprint; manipulating fingerprints in a bitwise fashion; creating new similarity metrics with mathematical expressions Supported file formats: SMILES and SMARTS	Microsoft Windows, Linux, Solaris	C, Fortran; Wrappers are available for Java and C++

Chemistry Development Tookit [131]	Interconversion between different types of representations; similarity calculation between two compounds; searching substructures using SMARTS; rendering chemical structures; algorithms for chemical graph theory; 3D conformer generation; various types of fingerprint calculation; generation of QSAR descriptors Supported file formats: SMILES, SMARTS, InChI, etc.	Microsoft Windows, Linux, MacOSX	Java

Open Eye Toolkit [132]	Real-time shape similarity for VS, lead hopping and shape clustering; molecule rendering and depiction; 2D molecular similarity calculation based on fingerprints; molecular property calculation and filtering; molecular docking and scoring; 3D conformer generation and superimposition. Supported file formats: SMILES, InChI, RDF, etc.	Microsoft Windows, Linux, MacOSX	C++; Wrappers are available for Python, Java, and.NET

ChemmineR [133]	Format Interconversions; similarity searching using various criteria such as atom pairs, PubChem fingerprints etc.; rendering chemical structure images; providing various types of clustering algorithms; searching PubChem database using various criteria such as Id, SMILES, etc.; and visualization functions for compound clustering Supported file formats: InChI, SMILES, SDF	Microsoft Windows, Linux, MacOSX	R

Indigo [134]	Exact matching, substructure matching, SMARTS matching; molecule fingerprinting, molecule similarity computation; molecular weight and molecular formula computation; combinatorial chemistry scripts; renderer plugin distributed together with the Indigo API Supported file formats: SMILES, SMARTS, RDF, etc.	Microsoft Windows, Linux, MacOSX	C++; Java, Python, Wrapper is available for .NET

**Table 5.** Libraries and toolkits for protein analysis (including VS)

Tools and libraries	Basic properties and included descriptors^a^	Operating systems	Programming languages
PROFEAT [95], ProPy [135], PyDPI [136]	AAC, DC, TC (ProPy and PyDPI), autocorrelation, CTD, CTriad (only PyDPI), SOCN, QSO, Pse-AAC, Am-Pse-AAC, topological descriptors (only PROFEAT), total amino acid properties (only PROFEAT)	Microsoft Windows, Linux	PROFEAT: Web server ProPy: Python PyDPI: Python

protr/ProtrWeb^b^ [137], Rcpi [138]	AAC, DC, TC, autocorrelation, CTD, CTriad, SOCN, QSO, Pse-AAC, Am-Pse-AAC, scales-based descriptors derived by PCA, factor analysis, and multidimensional scaling, BLOSUM/PAM matrix derived descriptors, PSSM profiles, similarity measures based on sequence alignment and GO annotation semantic similarity	Microsoft Windows, Linux, MacOSX	protr/ProtrWeb: Web server, R

camb [139]	AAC, DC, TC, autocorrelation, CTD, CTriad, SOCN, QSO, Pse-AAC, Am-Pse-AAC, Z-scales, T-scales, ST-scales, VHSE, MSWHIM, FASGAI, ProtFP8, BLOSUM62	Linux, Mac OS	C++, Java, Python, R

ProFET [140]	Various features based on biophysical quantitative properties, letter-based features, local potential features, information-based statistics, AA scale-based features, and transformed CTD features	Linux	Python

BLAST [141], ClustalW^c^ [142]	Heuristic pairwise sequence alignments/database search (BLAST), multiple sequence alignments (ClustalW)	Microsoft Windows, Linux, MacOSX	Web server, C, C++

DALI [143], MultiProt [144], TM-align [145], RCSB PDB Comparison Tool [146]	Protein global structure alignments	MultiProt:Linux, TM-align: Linux	All: Web server,TM-align:Fortran, C++

SiteEngine [147], APoc [148], eMatchSite [149], G-LosA [150]	Protein local structure alignments^d^	Linux	SiteEngine/eMatchSite: Web server G-LosA/eMatchSite: C++

POSSUM [151]	PSSM profile-based feature descriptors	Microsoft Windows, Linux, MacOSX	Web server, Perl, Python

GOSemSim [152]	Gene Ontology annotation semantic similarity	Microsoft Windows, Linux, MacOSX	R

FragHMMent [153]	Prediction of residue-residue contacts	Linux	Java

PSIPRED [154]	Secondary structure prediction	Linux	Web server, C

Naccess [155], POPS [156]	Solvent accessible surface area	Naccess: Linux, POPS: Microsoft Windows, Linux, MacOSX	Naccess: Fortran, POPS: Java

PocketPicker [157]	Prediction of protein binding pockets	Linux	PyMol plugin

SCREEN^c^ [158], trj_cavity [159]	Identification of protein cavities	trj_cavity: Linux	SCREEN: Web server, trj_cavity: C++

a Descriptor names are abbreviated according to information in Section 2.B Target Protein Descriptors.

b ProtrWeb only provides AAC, DC, TC, Autocorrelation, CTD, CTriad, SOCN, QSO, Pse-AAC and Am-Pse-AAC descriptors.

c ClustalW has been retired and replaced with Clustal Omega. The original SCREEN tool is also replaced with SCREEN2.

d These tools can also be included in ‘prediction of protein binding pockets’ part, which are mainly used for this purpose.

Open access web applications, online tools, data sets and source codes for VS, provided in the websites or in the supplementary material of the reviewed studies, are given in Table 6. Most of the VS studies in the literature describe methodologies and test them on various data sets, without providing open access web-services or tools that researchers can use to carry out their own analysis. The underlying reason is that, successful VS tools have potential to be employed in the pharmaceutical industry; as a result, the researchers often choose to develop commercial products with their methods. There are several commercial VS services and tools on the market. We did not provide any information regarding these commercial products, as they are out of scope of this study.

**Table 6.** Open access web services, online tools and data sets provided in the reviewed VS studies

Article	Method/tool name	Website	Resource type
Gfeller *et al.*[160]	SwissTargetPrediction	http://www.swisstargetprediction.ch	Web service

Shi *et al.* [161]	–	http://www.bmlnwpu.org/us/tools/PredictingDTI_S2 /METHODS.html	Source Code/Data set

Yabuuchi *et al.* [162]	–	http://msb.embopress.org/content/7/1/472	Data set (Supplementary)

Iwata *et al.* [11]	–	https://pubs.acs.org/doi/abs/10.1021/acs.jcim.5b00444	Data set (Supplementary)

Liu *et al.* [12]	SPACE	http://www.bprc.ac.cn/space	Web tool

Ma *et al.* [163]	–	https://pubs.acs.org/doi/abs/10.1021/ci500747n	Data set (Supplementary)

Koutsoukas *et al.* [164]	–	https://jcheminf.springeropen.com/articles/10.1186/s13321 -017-0226-y	Source Code/Data set (Supplementary)

Wen *et al.* [85]	DeepDTIs	https://github.com/Bjoux2/DeepDTIs_DBN	Source Code/Data set

Wallach *et al.* [165]	AtomNet	–	Commercial

Altae-Tran *et al.* [166]	DeepChem	https://github.com/deepchem/deepchem	Source Code/Data set

Soufan *et al.* [70]	DRABAL	https://figshare.com/articles/ DRABAL/3309562	Source Code/Data set

## Databases and gold-standard data sets

The aim of this chapter is to provide a brief overview of the open access chemical and biological data repositories and the available gold-standard data sets that are widely used in VS. Compound and target databases, together with the tools that they provide, are crucial for the development of novel VS methods. The databases for compounds, bioactivities and proteins and their statistics are given in Table 7.

**Table 7.** Databases of chemicals/compounds, bioactivities and target proteins, statistics and links

Compound and bioactivity databases	Statistics^a^			Website	Version
	Compounds	Targets	Interactions		
PubChem [1]	93 977 773 (C) 235 653 627 (S)	10 341 (P)	233 799 255 (I) 1 252 820 (E)	https://pubchem.ncbi.nlm.nih.gov	03.12.2017
ChEMBL [2]	1 735 442 (C)	11 538 (P)	14 675 320 (I) 1 302 147 (E)	https://www.ebi.ac.uk/chembl	v23
DrugBank [5]	9591 (D)	4270 (P)	16 748 (I)	http://www.drugbank.ca	v5.0
STITCH [167]	∼500 000 (C)	9 643 763 (P)	∼1.6 billion (I)	http://stitch-beta.embl.de	v5.0
BindingDB [168]	635 301 (C)	7000 (P)	1 419 347 (I)	http://www.bindingdb.org	03.12.2017
BindingMoad [169]	12 440 (C)	7599 (F)	25769 (I)	http://bindingmoad.org	Rel. 2014
KEGG [170]	18 211 (C) 10 484 (D)	976 (P)	6502 (I)	http://www.kegg.jp	Rel. 84.1
DCDB [171]	904 (D) 1363 (DC)	805 (P)	–	http://www.cls.zju.edu.cn/dcdb/index.jsf	v2.0
T3DB [172]	3673 (T)	2087 (P)	42471 (I)	http://www.t3db.ca/	v2.0

Side effect databases	Statistics^a^			Website	Version

SIDER [173]	1430 (D), 5868 (SE), 139 756 (A)			http://sideeffects.embl.de	v4.1

Metabolome databases	Statistics^a^			Website	Version

HMDB [174]	114 089 (M)			http://www.hmdb.ca/	v4.0

Chemical databases	Compounds			Website	Version
ChemSpider [3]	∼62 000 000 (C)			http://www.chemspider.com	03.12.2017
ChEBI [4]	53 495 (C)			https://www.ebi.ac.uk/chebi	Rel. 158
ZINC [175]	∼100 000 000 (C)			http://zinc15.docking.org/	ZINC 15

Target databases	Statistics^a^			Website	Version

AAindex [176]	AAindex1:566 indices, AAindex2:94 matrices, AAindex3:47 contact potential matrices			http://www.genome.jp/aaindex	Rel. 9.2
UniProtKB [80]	Swiss-Prot: 556 196 (P), TrEMBL: 98 705 220 (P)			http://www.uniprot.org	v2017_11
InterPro [177]	2128 (SF), 20 410 (F), 8840 (DM)			https://www.ebi.ac.uk/interpro/	v66
Pfam [178]	16 712 (F)			http://pfam.xfam.org/	v31.0
RCSB PDB [81]	125 799 (P)			https://www.rcsb.org/	28.11.2017
sc-PDB [179]	6326 (C), 4782 (P), 16 034 (I)			http://bioinfo-pharma.u-strasbg.fr/scPDB/	Rel. 2017
CATH [180]	6119 (SF), 434 857 (DM)			http://www.cathdb.info	v4.2
SCOPe [181]	2008 (SF), 4851 (F), 244 326 (DM)			https://scop.berkeley.edu	v2.06

a Abbreviations in the statistic column: compound (C), substance (S), drug (D) protein (P), protein family (F), interaction (I), experiments (E), associations (A), toxin (T), side effects (SE), drug combination (DC), metabolite (M), domain (DM), superfamily (SF).

### Compound, bioactivity and target protein databases

With the improvements in the drug screening technologies and VS methods, the amount of both the experimental bioassay data and computationally produced DTI data are increasing. Therefore, researchers require structured chemical and biological databases to store and publish this vast amount of data in a well-organized way. A chemical database of bioactive molecules (i.e. a compound database) is a resource that contains several properties of chemical substances such as 2D and 3D structures, physical and chemical attributes, molecular descriptors, side effects and clinical information, as well as targets and activity measurements. The public release of large-scale experimental bioactivity data, mostly from high throughput screening (HTS) assays, has started a new era in computational biomedical research. Research groups from all around the world have started to access and analyse the data, which boost the field of computational drug discovery (specifically VS) in the past decade. In this sense, the prominent bioactivity and compound data resources can be listed as PubChem [1], ChEMBL [2], DrugBank [5], STITCH [167], BindingDB [168], BindingMoad [169], KEGG [170], SIDER [173], DCDB [171], HMDB [174] and T3DB [172]. Although the discussed databases have common properties, they also complement each other by providing different features. For example, PubChem contains the largest bioactivity data for compounds—mainly retrieved from HTS experiments—and the other databases generally import data from PubChem. ChEMBL is also a large-scale compound and bioactivity database. However, one of the most significant differences of ChEMBL from the other large-scale sources is that the provided data are manually curated by experts from the literature in a comprehensive manner, making ChEMBL a more reliable resource, whereas the PubChem data are non-curated. ChEMBL also categorizes targets as ‘Single Protein’, ‘Protein Family’ and ‘Protein Complex’ and assigns a confidence score to state the specificity of compound activity. The main advantage of using PubChem over the other resources is its unmatched high volume (i.e. in terms of the number of bioassays, bioactivities, compounds and targets). Another bioactivity database BindingDB contains only experimentally validated bioactivity values of compound-target complexes without considering other functional assay results. BindingDB directly provides validation data sets for computational drug design studies. In contrary to PubChem, ChEMBL and BindingDB, BindingMoad is a small-scale bioactivity database, which includes high-resolution 3D structures of proteins and their ligand annotations for related protein-ligand interactions. In this sense, BindingMoad is especially convenient to be employed for the structure-based VS approaches. As an extensive network of biological systems, KEGG is a valuable resource for understanding functional hierarchies of biological events involving molecular interactions, pathways and disease mechanisms from molecular-level information of genes and genomes extracted from large-scale data sets of genome sequencing or other high-throughput experimental techniques. DrugBank database includes information regarding the approved and experimental drugs along with their target associations; hence, it is a small-scale database. However, DrugBank covers almost all aspects of drugs as a manually curated biomedical resource with high-quality standards. The data obtained from DrugBank is often used in test sets for novel large-scale VS methods. SIDER and STITCH are sister projects, where the former focuses on side effect information, and the latter focuses on the compound-target interactions under biological networks point of view. Therefore, it is quite common to combine complementary features from these databases, when applicable. In addition to the abovementioned resources, there are also useful databases such as DCDB, HMDB and T3DB, which focus on drug combinations, human metabolites and toxic substances, respectively. Considering these bioactivity databases, PubChem, ChEMBL, Binding MOAD and BindingDB represent activity data with quantitative measurements such as the IC_50_, EC50, Ki and potency, while DrugBank, STITCH, KEGG, DCDB, HMDB and T3DB only provide the information regarding presence of an activity/interaction between the corresponding drug-target pairs.

A protein information database includes protein sequences as well as their physico-chemical and biochemical properties, together with detailed functional annotation and structural information to provide data that can be used for various purposes, including function prediction and drug discovery. Many compound and target databases were constructed with the manual curation of the literature by expert scientists. Most of the databases also incorporate data from third-party resources and provide cross-references. UniProt is the main resource of protein sequence and annotation [80]. It presents a comprehensive protein repository, a central hub, importing and organizing vast amount of information from third-party protein resources as well. The PDB includes experimental protein structure information [81], which is crucial for structure-based VS studies, as well as for PCM. AAindex is a database of physico-chemical and biochemical properties of amino acids and a highly used resource for VS [176]. InterPro, Pfam, CATH and SCOP resources classify proteins in structural and functional groups/families, using pre-defined curated sequence motifs and structural domains [177, 178, 180, 181]. Further information about these data resources can be obtained from the supplementary material.

### Gold-standard data sets for VS

In machine learning, the term ‘gold-standard data sets’ refers to reliable sets of information created to address a particular problem, which can be used for the following purposes:

- development (i.e. training and testing) of computational methods;
- adjustment of the parameters of computational methods;
- evaluation of the performance of trained models; and
- benchmarking to compare the performances of various prediction models.

In VS, gold-standard data sets generally comprise manually curated compound-target pairs and their bioactivity values. The abovementioned data repositories provide data that can be used for model training and benchmarking; however, it is not easy to understand which database to employ at which step, to obtain the required data. Therefore, data set construction is one of the critical steps in VS studies. Although these databases provide cross-references to each other to some extent, the data are mostly disconnected, and it is often non-trivial to carry out data integration operations on different resources, which requires expert-level knowledge. As a result, expert curated gold-standard data sets are extremely valuable for the community.

Because of the lack of adequate experimental data and publicly available data repositories, it was a significant problem to define a suitable gold-standard data set for benchmark studies until 10 years ago. The early data sets were either too small or proprietary. For example, a data set generated in 1988 for comparative molecular field analysis included only 21 varied steroid structures for the analysis of their binding affinities to human corticosteroid- and testosterone-binding globulins [182]. In 2001, Hert *et al.* generated a data set for the comparison of different types of 2D fingerprints used in similarity-based VS with a total of 11 activity class, each of which was involving active compounds in a range of approximately 300–1200. However, this data set was derived from MDL Drug Data Report database, which is licensed and not publicly available [183].

As one of the first gold-standard data sets that is large enough and freely accessible, Yamanishi *et al.* created a data set with four classes (i.e. families) of targets that are enzymes, ion channels, G-protein coupled receptors (GPCRs) and nuclear receptors [90]. These target families are explained in the supplementary material. The data set by Yamanishi *et al.* involves only human proteins and was constructed using KEGG BRITE, BRENDA, SuperTarget and DrugBank databases and generated mainly for evaluating and training of their own VS method. This data set can be reached via: http://web.kuicr.kyoto-u.ac.jp/supp/yoshi/drugtarget/. The numbers of targets in these data sets are 664, 204, 95 and 26, whereas the numbers of DTIs are 2926, 1476, 635 and 90, respectively, for each class. An updated version of the data set was later created again by Yamanishi *et al.*, including the same target classes [184]; this time using the JAPIC database (http://www.japic.or.jp/). The numbers of the targets in the updated set are the same as previous data set, and the numbers of the interactions are 1515, 776, 314 and 44, respectively, for each class. Yamanishi’s sets were generated to train and test the performances of network/graph-based DTI prediction methods; thus, they are among the most used benchmarking data sets for network/graph-based approaches. However, they usually are not suitable for machine learning approaches, which require large training data sets. Yamanishi’s gold-standard sets can be downloaded from http://cbio.mines-paristech.fr/∼yyamanishi/pharmaco/.

Huang, Irwin and Shoichet have generated a benchmarking data set called directory of useful decoys (DUD) for testing VS methods, by curating challenging decoys that have a low probability of interacting with the selected targets. The DUD data set contained active compounds for the selected targets together with 50 decoys for each active compound, which have similar physico-chemical properties but different topology [185]. As an updated and enhanced version of DUD (DUD-E) with more diverse target classes such as GPCRs and ion channels (along with enzymes and nuclear receptors), DUD-E contains 22 886 ligands and their affinities against 102 targets retrieved from the ChEMBL database, together with property-matched decoys obtained from the ZINC database. The data set is freely available at http://dude.docking.org [186].

Another benchmark data set designed for VS is maximum unbiased validation (MUV), which was generated from PubChem bioactivity data by topological optimization based on a refined nearest neighbour analysis. MUV provides randomly distributed sets of active compounds—selected from potential actives—and inactive compounds—selected from potential decoys—that minimizes the influence of data set bias on validation results. The workflow used for the generation of optimized MUV data set is also freely available as a software package that can be applied on other activity data sets for optimization. The data set and the software package can be accessed via https://www.tu-braunschweig.de/pharmchem/forschung/baumann/muv [187].

In 2012, Merck sponsored a drug target interaction challenge over Kaggle data competition service (https://www.kaggle.com/c/MerckActivity). They provided 164 024 compounds for 15 biologically relevant targets. For each activity, they provided a list of chemicals along with their molecular descriptors and bioactivity measurement values. The participating teams tried to predict the experimentally known held-out interactions among the overall data set. The evaluation mechanism and the performance results of the teams are available in the competition page. Following the end of the competition, the held-out evaluation sets were released, which can now be used as benchmarking data sets for different VS approaches. The data sets are explained in the publication by Ma *et al.* [163] and available at https://www.kaggle.com/c/MerckActivity/data.

Another data set called Tox21 is also commonly used in machine learning-based computational drug discovery applications. This data set has been generated by The Tox21 Data Challenge community in 2014 to evaluate the performances of different computational methods in terms of toxicity prediction. The data set comprises approximately 12 000 environmental chemicals and approved drugs screened in 12 different bioassays related to nuclear receptor signalling and stress response pathways to reveal their toxic effects based on the disruption of these processes [188].

There are also novel approaches for generating gold-standard data sets, especially for deep learning applications in DTI prediction. Wu *et al.* developed a platform, MoleculeNet, as a benchmark collection for machine learning methods used in molecular systems. The curated data set of MoleculeNet contains nearly 700 000 compounds retrieved from publicly available databases such as QM7/QM7b, QM8, QM9, ESOL, FreeSolv, Lipophilicity and PDBbind for regression data sets and PCBA, MUV, HIV, BACE, BBBP, Tox21, ToxCast, ClinTox and SIDER for classification data sets. The data were split into training/validation/test subsets and tested on a range of categories, such as quantum mechanics, physical chemistry, biophysics and physiology. Furthermore, MoleculeNet provided evaluation metrics and open-source implementations of several well-known molecular featurization methods and machine learning algorithms. All parts of MoleculeNet have also been integrated into DeepChem open-source framework (https://github.com/deepchem/deepchem) [189]. Apart from these gold-standard sets, there has also been efforts to generate purpose specific data sets [190], often using the ZINC database [175] as their resource. With the increased volume of open access experimental data in repositories such as PubChem, ChEMBL and ZINC the data resources for VS studies has been significantly changed, compared with 10 years ago. Novel data sets derived from these resources such as the DUD and MUV, together with the new algorithmic approaches, are highly promising in terms of developing the field of computational drug discovery. The field of generating and utilizing gold-standard/benchmarking data sets for VS has been extensively discussed in the recent works by Lagarde *et al.* and Xia *et al.* [190, 191].

## Machine learning applications in VS

The field of machine learning has been extensively reviewed and discussed in several books [192]. There are two main approaches in machine learning literature in terms of how the learning process is carried out, supervised learning and unsupervised learning. In supervised learning, the objective is to infer a function that maps the input data to the output class labels [193], whereas the aim in unsupervised learning is to learn the hidden structure of input data without having class labels. Unsupervised learning algorithms employ techniques to discover relationships among the non-labeled input samples. The most popular applications of unsupervised learning are clustering and dimensionality reduction. Once the groups and clusters are obtained with the application of an unsupervised learning method, each group can be inspected to assign semantic meanings by experts [194]. Both supervised and unsupervised machine learning techniques are used in cheminformatics on a wide range of topics, including VS [195–206], yet most of the methods so far assumed the supervised approach. The subject covered in this chapter is mostly the supervised learning applications in VS. A plethora of methods has been proposed for VS purposes in the past decade. These VS methods use experimentally validated compound-target pairs and their features along with the bioactivity information to create predictive models for future predictions of activities.

In terms of the methodological utilization of the input properties, VS methods can be divided into similarity-based and feature-based methods, although there is no such technical classification in the machine learning literature [192, 193, 201, 207]. In the following sections, similarity-based and feature-based VS methods are investigated, which is followed by the recently popularized deep learning-based applications in VS. For this, we have mostly focused on the studies published in the past 3 years, some of which have aims beyond DTI prediction (e.g. estimation of beneficial drug-drug combinations or ATC code prediction). There are numerous examples of especially ligand-based DTI prediction methods that are highly cited in the literature. We chose to leave these articles out of this review because of they were published more than 5 years ago and were the subject of previous VS field review papers.

### Similarity-based approach

Similarity-based methods rely on the assumption that biologically, topologically and chemically similar compounds have similar functions and bioactivities and, therefore, they have similar targets [160, 161, 197, 208]. In the similarity-based approach, the target associations of similar compounds (or the compound associations of similar target proteins) are transferred between each other. Therefore, transfer approach is a term used interchangeably to define similarity-based methods. In chemical space, similarities are calculated by searching molecular substructure and isomorphism based on the representations of molecules such as SMILES and InChI. In target space, similarities are mainly calculated by sequence alignment methods. The methods under this approach construct similarity matrices either for compounds or targets, or for both of them [207]. Subsequently, constructed similarity matrices are used by the machine learning models. Below, we provided reviews for three similarity-based VS methods, which were published in the past few years.

With the aim of identifying biologically and structurally similar clusters of compounds, weighted clustering was proposed by integrating multiple similarity matrices [197]. Two data sets were used: the epidermal growth factor receptor (EGFR) and the fibroblast growth factor receptor (FGFR) data sets. EGFR data set contained bioactivity assay readouts and gene expression profiles for 35 compounds and 3595 genes. In FGFR data set, the chemical structure information, gene expression data and bioactivity assay readouts were available for 94 compounds and 1056 genes. Two similarity matrices were generated based on the structural and the phenotypic properties. Structural properties of compounds were represented by ECFP6 fingerprints, and similarities of compounds were calculated using the Tanimoto coefficient. For the phenotypic similarity matrix calculation, bioactivity readouts were used. The Euclidean distance was employed to calculate the phenotypic similarities between two compounds based on their bioactivity results on the same assays. Subsequently, generated similarity matrices were used to perform clustering using a weighted clustering algorithm. The weighted clustering technique was shown to be more efficient in terms of identifying structurally and biologically similar compounds compared with the individual clustering methods.

A supervised similarity-based PCM method was described for the detection of: (i) interactions between new drug candidates and known targets and (ii) interactions between new drug candidates and new targets [161]. The similarity between two compounds was measured by a combination of non-structure-based score (ATC-based semantic similarity score) and 2D graph structure-based score. ATC-based semantic similarity score was calculated by counting the common subgroups between ATC code annotations of two compounds. 2D structure-based similarity calculation was performed by aligning graph structures of compounds. The similarity score for a pair of targets was computed using a combination of a functional-similarity-based (using Enzyme Commission -EC- numbers) score and a sequence-based similarity score. Functional similarity-based score was calculated by counting the number of common EC number annotations. For sequence-based similarity score calculation, subsequences in the ligand-binding domains were extracted, and they aligned the extracted subsequences to calculate similarity scores between targets. The data sets constructed by Yamanishi *et al.* [90] for four classes of targets (i.e. GPCRs, ion channels, enzymes and nuclear receptors) were employed for the tests. A concept called ‘super-target’ was proposed to overcome the problem of the scarcity of training instances in terms of targets. Similar targets were clustered, and it was assumed that if the drug interacted with a target, it would also interact with the other targets in the same super-target cluster. For the prediction of new drug candidates for a known target, the following methodology was pursued: When a new compound was given as input to the system, for each known target *t_x_*, a confidence score was calculated between the query compound and the super-target cluster that *t_x_* belonged to, based on the drug associations of the targets in that super-target cluster. Subsequently, another confidence score was calculated between query compound and *t_x_* based only on the drug associations of *t_x_*. Finally, these two scores were combined as a single prediction score. For the prediction of new drug candidates for a new target, a similar procedure was followed. In this case, the new target was considered as a member of most similar super-target cluster based on its functional and sequence similarities.

SwissTargetPrediction is a supervised similarity-based method that combines 2D similarity and 3D similarity of compounds with the aim of identifying new targets for query compounds [160]. ChEMBL database was employed to obtain known compound–target pairs. The training data set consisted of 280 381 small compounds for 2686 targets. When a compound was given as input to the system, a combination of 2D and 3D similarity scores were calculated between the query compound and all compounds with known targets. To obtain 2D similarity score, a compound was represented by FP2 fingerprints and the 2D similarity scores between the query compound and other compounds were calculated by the Tanimoto coefficient. For the 3D similarity score, 20 different conformations of compounds were generated, and the Manhattan distance was used to calculate distances among all conformations of two compounds. The smallest distance was then chosen among the 20×20 distance scores, and it was converted into a 3D similarity score. 2D and 3D similarity scores were combined as a single prediction score for targets. Finally, the system outputs a ranked list of targets based on the combined similarity scores. Users can get predictions for a compound using SMILES string of the query compound or by drawing 2D structure of compounds using the web tool provided. SwissTargetPrediction is available at http://www.swisstargetprediction.ch.

### Feature-based approach

**Figure 4.**
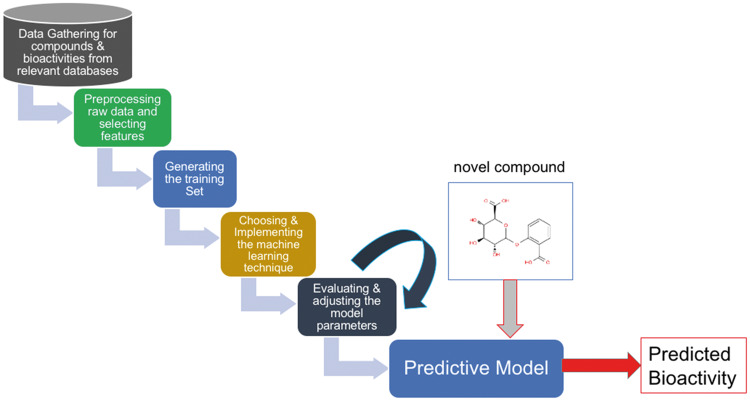
The steps of a typical feature-based virtual screening method for training a predictive model.

In Feature-based VS methods, each instance (i.e. compound and/or target) is represented by a numerical feature vector, which reflects various types of physico-chemical and molecular properties of the corresponding molecules. Targets are usually modelled using their physical and chemical properties and subsequence distributions or functional attributes, whereas the compounds are usually modelled using structural properties. In a typical feature-based VS application, a set of compounds that is known to interact with a specific target is extracted from compound and bioactivity databases. Subsequently, feature vectors are generated for each compound. Finally, the constructed feature vectors are fed to a machine learning algorithm to create a predictive model for the interaction with the corresponding target. When a new query compound’s feature vector is given to the trained model as input, the output of the predictive model is either active or inactive against the corresponding target protein (Figure 4). This is the so-called ligand-based approach in terms of the incorporated input feature types (i.e. compound features). PCM methods also assume a similar methodology, but they jointly model the target properties at the input level along with the compounds, so that the query can be a compound–protein pair, and the model predicts the presence of that specific interaction. Examples of feature-based VS methods are given below.

A supervised machine learning methodology was proposed by Liu *et al.* [12] using a combination of both similarity and feature-based approaches to predict drug–ATC code associations. DrugBank database was employed to create their positive and negative training data sets. The total set was composed of 1333 small molecule drugs and their ATC codes at various levels. ATC code prediction problem was described as a binary classification problem. Therefore, for each ATC code, a positive training data set and a negative training data set were constructed. Known drug-ATC code pairs were retrieved to construct the positive training data sets. To construct a negative training data set for each ATC code, they first removed the positive drug–ATC code pairs from all possible drug–ATC code pairs and randomly selected samples from the remaining set. Then six scores were defined to calculate drug–drug similarities, which are based on chemical structures, functional groups, target proteins, drug-induced gene expression profiles, side-effects and chemical–chemical associations. Each drug was represented as a six-dimensional feature vector. The value of a certain feature was determined by taking the largest similarity score between the input drug and the drugs associated with the corresponding ATC code. Once the drugs were converted into feature vectors, the logistic regression method was used to train predictive models for each ATC code. When a new query compound is given to the system, first, it is converted to the feature vector based on the similarity values; then, it is given to the predictive models as input to predict the candidate ATC codes. The method, SPACE, is available at http://www.bprc.ac.cn/space.

In the work by Cano *et al.* [68], the main objective was the inherent selection/ranking of features (see wrappers in feature selection section of supplementary material) and training a DTI prediction classifier using random forest. Directory of Useful Decoys (DUD) was used to create their training data set, which was composed of kinases, nuclear hormone receptors and other proteins. The constitutional, charged partial surface area and fingerprint-based descriptors were the input to the system. The performance of the model was compared with support vector machine (SVM) and neural network classifier-based models, and the random forest classifier was successful to select and rank most representative features, given a large set of input features. In this setting, it was also observed that a reduced number of features drastically decreased the computational complexity of DTI prediction models.

For drug repurposing, a combination of similarity and feature-based supervised method was proposed by integrating drug/compound, target protein, phenotypic effect and disease association data from several sources [54]. The chemical structures of drugs and compounds were retrieved from the ChEMBL database. Three different molecular descriptors were used to represent compounds, which are ECFP4), Chemistry Development Kit (CDK) Fingerprints, and KEGG Chemical FunKCF-S. The compounds were, thus, represented by 1024, 1024 and 475 692 dimensional fingerprints. The obtained feature vectors were referred to as the ‘chemical profile’ of the compounds. Phenotypic effects of drugs were obtained from FDA Adverse Event Reporting System, and each of the 2594 drugs were represented as a 16 075-dimensional feature vector, where each dimension represents the presence or absence of a phenotypic effect. This data set was named as the ‘phenotypic profile’ of a drug. Compound–target interactions and the bioactivity values were obtained from seven different databases. Their total activity set comprised 1 287 404 interactions involving 519 061 compounds and 3736 targets. This data set was referred as the ‘chemical protein interactome data set’. Molecular features of diseases were obtained from the International Classification of Diseases (ICD10) and the KEGG DISEASE database. The diseases were represented as 6342-dimensional binary feature vectors, where each dimension represents presence or absence of a molecular feature. Drug–disease associations were obtained from medical books and from the KEGG DRUG database. This data set comprised 5830 drug-disease associations involving 2271 drugs and 463 diseases. Disease–target associations were obtained from the KEGG DRUG database. They created a data set consisting of 2062 disease-target associations for 250 diseases and 462 therapeutic target proteins, and this data set was named as the ‘disease-target association template’. Their prediction method was composed of three parts, which were called as the Target Estimation with Similarity Search (TESS), Indication Prediction by Template Matching (IPTM) and Indication Prediction by Supervised Classification (IPSC). In TESS, the aim was to predict potential targets of a given drug based on similarity search. Each compound was represented by a 3736-dimensional target interaction profile. The similarity search was performed against the compounds in the chemical–protein interactome data set based on the chemical and phenotypic profiles of the compounds. Subsequently, for each target, the compounds that were associated with the corresponding target were retrieved, and the drug-target similarity score was assigned using the similarity score between query drug and the most similar compound that were associated with the corresponding target. In IPTM, the aim was to predict novel drug indications for the query drugs. First, target proteins of the query drug were retrieved. For each target, the diseases that were associated with the corresponding target were obtained from the disease target association template. This way, the query drug was linked to the diseases based on their target associations. In IPSC, the aim was to predict novel drug indications using a supervised classification method. In this method, target proteins of the query drug and molecular features of diseases were used. Each drug–disease pair was represented by a feature vector, and drug indication prediction was formulized as a binary classification problem, where the output of the regression-based classifier shows if the drug could be applicable to the paired disease. The cross-validation results showed that IPTM and IPSC methods outperformed the previous methods from the literature.

A supervised feature-based PCM method was proposed for GPCR and protein kinase targets [162]. The positive training data set was generated using the GLIDA database by extracting experimental compound-target interactions, containing 5207 interactions for 317 targets and 866 compounds [209]. Negative training samples were generated among the unknown compounds-target pairs. Compounds were converted into 929-dimensional molecular descriptors. Descriptors for targets were generated using a string kernel, resulting in 400-dimensional feature vectors. Two vectors, that is, compound and target descriptors, were then concatenated for each positive and negative interaction. Finally, the generated feature vectors were fed to an SVM classifier to train predictive models for each target family. Selected novel drug predictions were also experimentally validated for both GPCR and protein kinase families.

A supervised feature-based PCM method for the identification of novel drug combinations was described by Iwata *et al.* [11]. Orange Book and the KEGG databases were proposed to extract beneficial drug–drug combinations [170, 210]. Interacting drug–target pairs were collected from seven different databases. Furthermore, 4007 DTIs were incorporated for 588 drugs and 930 targets. Each drug was represented by a 1078-dimensional binary feature vector, where 930 of them represent the presence or absence of each target, and 148 of them represent the presence or absence of ATC code annotations. Subsequently, each drug–drug pair was represented as a binary feature vector by combining individual feature vectors of the corresponding drug pairs. Finally, the obtained feature vectors were fed to a logistic regression classifier. When a new drug–drug pair is given as a query to the system, the output was calculated as potentially beneficial or not.

Another supervised PCM method was proposed for DTI prediction [211]. In this approach, compounds were represented using fingerprints, and targets were expressed as sequence alignment-based profiles. First, the position-specific scoring matrices were generated for all target protein sequences. Subsequently, a local binary pattern method was adapted to extract features from position-specific scoring matrices. In the end, targets and compounds were represented by 256- and 615-dimensional feature vectors. Next, principal component analysis was applied for both target and compound feature vectors to obtain an uncorrelated and a reduced number of features. Four different data sets were employed: enzymes, GPCRs, ion channels and nuclear receptors. The positive samples were interacting compounds-target pairs, and same number of negative samples were selected randomly from remaining interaction sets. Finally, obtained features were fed into discriminative vector machine classifier which was proposed by the same group. Support vector machine classifier based on the same features was trained, and the performance of two classification methods was compared. The results were compared with three conventional methods, and this method had a better performance.

In terms of the methodological approach used in modelling the pairwise relationships, a highly studied topic is the development of network or graph analysis-based DTI prediction methods. In these methods, compounds and targets are represented as nodes on a graph, where the edges connecting these nodes indicate interactions. Modelled this way, estimation of unknown DTIs becomes a link prediction task. Various techniques, borrowed from the fields of graph theory and social and biological network analysis, are employed to solve the problem at hand. Frequently, the relationships in-between the compounds (i.e. in terms of molecular/structural similarities) and in-between the targets (i.e. in terms of homology or protein-protein interactions) have been incorporated in the generated networks to enrich the input information. An advantage of the network/graph-based approach is that the system can work well even when the number of training instances is low. Network/graph-based DTI prediction methods can be similarity-based, feature-based or a combination of both. One seminal work on this subject is by Yamanishi *et al.* [184], where the authors integrated both the similarities within the genomic space (using pairwise sequence alignment) and within the chemical space (using molecular and pharmacological effect similarities) in their network, for the first time. In this study, chemical, pharmacological and genomic spaces are unified and used together with the known DTIs to generate predictions for target families of enzymes, ion channels, GPCRs and nuclear receptors. In another study, Gönen [212] incorporated target protein sequence similarities and compound molecular structure similarities in a kernelized Bayesian matrix factorization framework to predict unknown DTIs. Other examples for network/graph-based methods can be given as Shi *et al.* [161], Sawada *et al.* [54] and Li *et al.* [211], which are reviewed in this study. It is also important to note that the gold-standard data set generated by Yamanishi *et al.* (explained in the section entitled: ‘Gold-Standard Data Sets for VS’) is suitable for testing network/graph-based DTI prediction methods.

In a review study by Chen *et al.*, the available resources for DTI prediction were presented, including databases, web servers and computational methods [213]. Methodological approaches were categorized as graph/network-based, machine learning-based and other methods, and the advantages and disadvantages of each approach were discussed. For graph/network-based drug discovery, the integration of different network models and sequencing technologies has been indicated to provide significant improvements for personalized medicine. As a suggestion to further improve the DTI prediction performance, the employment of heterogeneous training data by combining different data sources was recommended. The graph/network-based approach (excluding artificial neural networks), which was highly employed in the DTI studies, especially between 2006 and 2013 [67, 90, 184, 212, 214–218], was mostly left out of this study to focus on novel DTI prediction approaches.

Both the similarity and the feature-based approaches are used extensively in the literature. One of the main advantages of similarity-based approach is that when the problem involves heterogeneous data, different types of similarity matrices can be combined in the same model. Another advantage of similarity-based methods is that, sophisticated kernel methods can be applied [207]. They are also relatively simple and easy to model. However, they are computationally not practical to apply on large data sets, as they require extremely high number of similarity calculation operations. Considering the feature-based methods, one advantage is that, they can reveal intrinsic properties of compounds and targets that play critical roles in DTIs, which leads to more interpretable results. Another advantage is that, a problem-specific feature selection can be performed to obtain relatively more accurate predictions. One of the challenges about the feature-based methods is the selection of negative samples for the construction of negative training sets. Although chemical databases include experimentally validated DTIs, they do not provide sufficient number of experimentally validated non-interacting compound–target pairs. When this is the case, the frequently employed approach for negative sample selection is to randomly select pairs from the set remained after excluding the positive training samples. However, this approach is problematic since the randomly selected pairs may also include pairs that are interacting, which is unknown (and therefore not recorded in the source database) so far. Negative sample selection is not only a problem for the VS field but also a problem for cheminformatics and bioinformatics in general [219–221]. There are alternative algorithmic methods to construct more reliable negative training data sets [207, 213, 222–224]. The lack of sufficient negative training data sets also leads to the class imbalance problem, which highly affects the prediction performances of computational systems. The class imbalance problem may produce a bias towards the class having most training samples, causing the model to give excessive number predictions for this class, resulting in a high number of false positive (FP) predictions. In their recent studies, Soufan *et al.* focused on the class imbalance and FP prediction problems. Models using five different solutions were trained to overcome class imbalance problem and the performances of these systems were compared. Classifier performance aware methods were also used along with several evaluation metrics to reduce the FP rates [70, 71]. Another challenge for the feature-based methods is the high-dimensionality of feature vectors, which can reach the order of millions [67, 225]. Extremely high-dimensional vectors create computational overhead, and they often lower the accuracy of predictions. Usually combining different types of informative features increases the performance of classifiers; however, after a certain point, adding more features to the system starts to decrease the performance, which is known as *curse of dimensionality* [226]. Therefore, feature-based methods may require the application of feature selection techniques to reduce dimensions and keep only the most relevant and distinctive features in the model. Various studies have been performed to analyse and compare feature reduction and selection techniques in the literature [227–232], which also have been discussed in the supplementary material.

### Deep learning applications in VS

**Figure 5.**
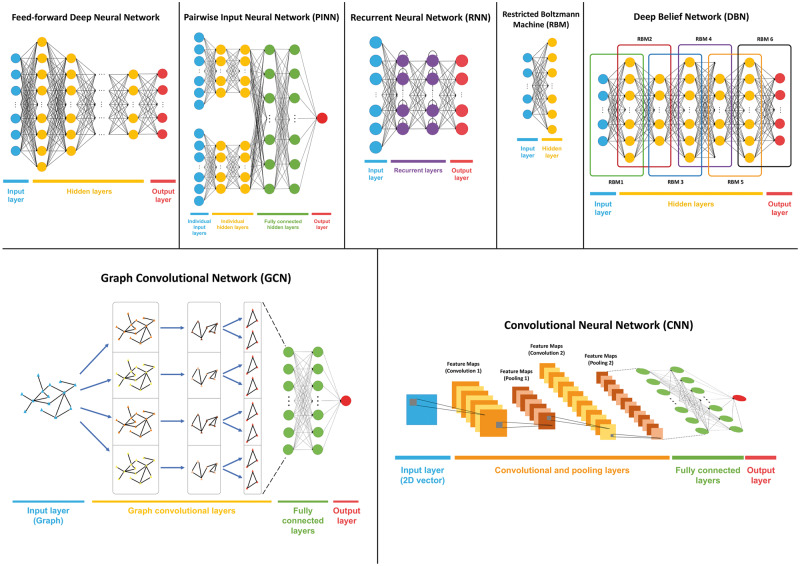
Schematic representations of different DNN architectures frequently used in the literature.

Deep learning algorithms have been extensively used in recent years because of their successful results in computer vision, speech recognition and bioinformatics [233–236]. The term *deep learning* represents a group of machine learning approaches, which contain multiple data processing layers. Deep learning algorithms yield successful learning of the representations of the input data through multiple levels of abstraction [237]. Deep neural networks (DNNs) are artificial neural network methods that have multiple hidden layers. In this sense, DNNs are considered as a group of deep learning algorithms. DNNs convert the low-level features obtained from the input into more and more complex features in each subsequent layer. An example of a basic feedforward DNN (i.e. a multilayer perceptron—MLP) architecture is given in Figure 5, along with other popular DNN architectures. In this figure, nodes correspond to neurons and the edges between nodes correspond to neural connections, where the signal is transmitted. According to the model choice, neurons at different layers can be fully connected to each other or not. At each neuron, a non-linear activation function, whose coefficients are determined during the training procedure, takes the input signal from multiple connected neurons at the preceding layer and modifies it before transmitting it to the next neuron. A standard feedforward artificial DNN has three different types of layers: the input layer, hidden layers and the output layer, each of which are composed of multiple parallel-connected neurons. A neural network with two or more hidden layers is considered as a DNN [233]. The input features are directly fed to the input layer and after a number of non-linear transformations using hidden layers, the predictions are generated at the output layer. Each output node corresponds to a task (i.e. class) to be predicted. If there is only one node in the output layer, then the corresponding network is referred as a single-task DNN. Otherwise, it is called a multi-task DNN.

A deep learning algorithm won the Kaggle Virtual Screening Challenge, which was sponsored by Merck, and it drew considerable attention to employing deep learning techniques for VS purposes [163, 238]. Recently, it was shown that deep learning algorithms outperformed the state-of-the-art methods in numerous VS studies [164, 165, 195, 238–243]. Several advantages of deep learning architectures have been reported for VS:

- deep learning algorithms inherently build relationships between multiple targets; therefore, they are suitable for multi-task learning;
- they provide higher-level abstractions by building complex features from raw input data in a hierarchical manner and are able to identify the unknown structure in the data, and the observed high performance of DNNs is usually attributed to this ability;
- shared hidden units among the targets enhance the prediction results of the targets having less training samples.

There are several DNN techniques (or architectures), and each has advantages and disadvantages according to the nature of the data being analysed and the types of features employed. The most commonly used ones can be listed as feedforward DNNs with multiple hidden layers [163] which can be considered as the standard application, deep convolutional neural networks–CNNs–(highly used in computer vision), where each of the several convolutional layers will capture a specific feature from the multi-structured input data [165, 239], and pairwise input neural networks (PINNs), where the features belonging to compounds and proteins can be fed to the model together [245]. DNN-based techniques are also divided into two according to the number of prediction tasks in a model, such as the single-task and multi-task DNNs. Single-task networks are modelled in such a way that one model can only produce answer for one specific question (e.g. is there an interaction between this compound–protein pair) [163], whereas multitask networks are modelled to infer multiple unknowns in one model (e.g. which of the 20 potential target proteins can interact with the input compound) [238]. All of these DNN architectures can be considered under the title of feature-based machine learning methods. Below we review a large collection of studies of deep learning applications in computational drug discovery with an emphasis on DTI prediction. Table 8 summarizes frequently used DNN architectures in the field of VS and groups the reviewed studies in terms of the employed DNN architectures. Figure 5 shows the schematic representations of those DNN architectures explained in Table 8.

**Table 8.** Deep learning architectures together with the VS studies that use each architecture

Architecture name	Description	DNN-based VS studies		
		Citation	Input protein features	Input compound features
Feedforward DNN – FFDNN (an interchangeably used term in some of the resources: multilayer perceptron–MLP)	A feedforward DNN can be considered as the most basic DNN architecture, which has multiple hidden layers that are usually fully connected to each other (Figure 5). These networks are mostly structured to predict multiple number of tasks (usually targets in DTI prediction) in a single model (i.e. multi-task networks)	Dahl *et al.* [238]	–	Several different molecular descriptors
		Ma *et al.* [163]	–	Atom pairs and donor-acceptor pair descriptors
		Unterthiner *et al.* [243]	–	ECFP12
		Ramsundar *et al.* [242]	–	ECFP4
		Koutsoukas *et al.* [164]	–	ECFP4

Pairwise input neural network (PINN)	PINNs are feedforward neural networks that take two different feature vectors as input and predicts their relation as output. In some of the PINN applications, the two individual input vectors are processed by separate groups of neurons before they are merged at a subsequent fully connected layer. PINNs are especially suitable for the prediction of pairwise relations such as DTIs	Wang *et al.* [244]	Binding sites	2D structural fingerprints
		Wan *et al.* [245]	Amino acid triplets in protein sequences	2D structural fingerprints
		Lenselink *et al.* [246]	Physicochemical properties	Morgan fingerprints

Recurrent neural network (RNN)	RNNs are specialized artificial neural networks that contain feedback loops to extract patterns using not only the current input but also the previously perceived inputs. RNNs successfully extract patterns from sequential data such as texts, protein sequences, audio signals and time series data. RNNs mainly have applications in speech recognition	Goh *et al.* [247]	–	SMILES strings

Restricted Boltzmann machine (RBM)/Deep belief network (DBN)	RBMs are single layer generative artificial neural networks, which can learn probability distributions given the training data. DBNs are constructed by stacking RBMs to solve more complex problems. Different from FFDNNs, DBNs are trained stack-by-stack. DBNs are used in several applications such as clustering and generating objects such as images.	Wen *et al.* [85]	Sequence composition descriptors	2D structural fingerprints
		Wang *et al.* [248]	Direct (e.g. compound-target binding) and indirect (e.g. compound changes the level of expression of the target) interactions on the multidimensional DTI network	

Convolutional neural network (CNN)	CNNs inherently extract the features hidden in the input samples by applying sequential layers of convolutions and pooling modules. The convolution layers extract local patterns (sub-features) by moving a window over the sample and the pooling layers are used to sub-sample and reduce the features. CNNs are mainly used in image processing applications.	Wallach *et al.* [165]	3D binding sites	3D structures of compounds
		Gonczarek *et al.* [249]	Binding pockets	3D structural fingerprints
		Goh *et al.* [250]	–	2D structure images of compounds

Graph convolutional neural network (GCN)	GCNs are created by applying convoluting operations on graph encodings. GCNs can be used to model any entity that is expressed as a graph such as social networks and chemical compounds.	Kearnes *et al.* [251]	–	2D graphs of compounds
		Altae-Tran *et al.* [167]	–	2D graphs of compounds

One of the early studies employed multi-task feedforward DNNs for the prediction of activities of compounds against 19 target assays from the PubChem database [238]. Active and inactive labels of compounds were used against each of the 19 targets, and the training data set comprised 69 396 active and 70 331 inactive compounds. The problem was stated as a classification problem, where inputs were the compound descriptors, and outputs were the presence of interaction against the modelled targets. Furthermore, 3764 dimensional molecular descriptors were generated to represent the compounds. The performance of a multi-task neural network was compared with random forests, gradient boosted decision tree ensembles and logistic regression methods. The results showed that multi-task neural networks performed best in most of the cases. The performance of single-task and multi-task neural networks was compared as well, and multi-task neural networks achieved better performance in the test cases. Feature selection was further performed. However, no significant performance gain because of feature selection was observed.

To select hyper-parameters and compare single-task and multi-task DNNs, Ma *et al.* [163] made use of Merck's Kaggle challenge data set along with the Merck's in-house data sets. Each compound was represented as molecular descriptors based on atom pairs and donor–acceptor pair descriptors. In total, there was 30 data sets, which included 129 295 unique compounds. Several models were created using different hyper-parameters, and it was reported that the use of a single set of hyper-parameters can perform better than using optimized parameters for different data sets. The performance was compared with the performances of models trained with random forest classifier and DNNs achieved higher performance. Furthermore, on the average, multi-task DNNs obtained better prediction performance than the single-task DNNs. The performance of the single-task DNNs was reported to increase with the increasing size of training data sets.

In another early study, Unterthiner *et al.* [243] used multi-task DNNs for the prediction of activities of compounds for targets. ChEMBL database was used to obtain known compound–target interactions and the corresponding bioactivity values, which were discretized as active, weakly active, weakly inactive and inactive based on pre-defined bioactivity thresholds. This way, a data set was generated that comprised 2 103 018 (972 268 active—1 130 750 inactive) bioactivity measurements distributed across 5069 targets and 743 336 compounds. In the models, each compound was represented as about 13 million dimensional fingerprints using ECFP12 features and then the number of features were reduced to 43 340 dimensions by discarding the features that were absent in the majority of compounds. Finally, multi-task DNNs were trained where the inputs were compound feature vectors and the outputs were target activity values. The performance of their multi-task neural network was compared with support vector machine, binary kernel discrimination, logistic regression, *k*-nearest neighbour and Parzen-Rosenblatt methods. Multi-task neural network outperformed all other algorithms.

A particular type of DNNs, pyramidal multi-task DNNs was described and applied for VS [242]. In this pyramidal architecture, layers are organized such that each layer has less number of neurons than its previous layer. Training data sets were collected from four different publicly available data sources, which consisted of nearly 37.8 million experimental compound–protein interactions for 1.6 million compounds and 259 targets. The compounds were represented by ECFP4 fingerprints. Several experiments were conducted by changing the number of tasks and training samples in their models. The performance of pyramidal multi-task neural networks was compared with logistic regression, random forest, single-task neural network, pyramidal single-task neural network and one-hidden layer multi-task neural network. Pyramidal multi-task neural network performed best among the other methods. The following important observations were reported:

- the multi-task deep architecture achieved significant improvement over standard machine learning algorithms;
- the performance of multi-task networks increased as more tasks and data points were added;
- shared bioactive compounds among targets had a significant positive impact on performance.

The main difference between the study by Ramsundar *et al.* and the study by Unterthiner *et al.* is that, the number of known ligands for each target was much higher in this study (i.e. ∼2 million samples for 1230 targets versus ∼40 million samples for 259 targets). In addition, the main concern of the study by Ramsundar *et al.* was to discover the causes of performance changes based on parameter selections (i.e. number of tasks, training data sizes and layer organizations), whereas in Unterthiner *et al.*, the main aim was to demonstrate the performance gain of multi-task DNNs over other baseline methods.

An investigative study was performed for virtual screening by Koutsoukas *et al.* [164], using single-task feedforward DNNs. Their study was composed of two major parts: first, the effects of the hyper-parameter choices on the performance were investigated. In the second part, the aim was to compare the DNNs with other types of classifiers in terms of performance. ChEMBL database was used to create training data sets for seven different targets from diverse protein families and an individual prediction model was constructed for each target. Furthermore, 7218 active compounds were tested against these targets, and the compounds were represented as 1024-dimensional molecular fingerprints. The rectified linear unit activation function performed better than the other activation functions during the experiments. It was also reported that the number of neurons at each layer that give the best performance was highly dependent on the data set and should be determined separately for each model. The drop-out regularization helped to gain better performances around 50% drop-out rate. In the second part of the study, the performance of DNNs was compared with Bernoulli Naive Bayes, *k*-nearest neighbour, random forest and SVM classifiers, and DNNs outperformed all of them.

PINNs where inputs represented pairs of target–ligand feature vectors are also a popular type of DNNs. Pursuing a PCM approach, Wang *et al.* considered target–ligand interaction as a binary classification problem, where inputs represented pairs of target-ligand feature vectors, and the binary output represented the interaction prediction for the corresponding pair [244]. The training data set was obtained from sc-PDB database and comprised 836 targets, 2710 ligands and 6830 target-ligand pairs [179]. Binding sites of proteins were used as target features, which were represented as 199-dimensional vectors. The compounds were represented as 413 dimensional fingerprints. Subsequently, each known interacting target and ligand pair was labelled as a positive example, and the remaining pairs were considered as the negative examples. This information was used then to train a four-layered pairwise neural network model. The method achieved better performance than the conventional methods from the literature in terms of several criteria.

Wan *et al.* [245] proposed a DNN for DTI prediction. Their framework also included an unsupervised representation learning for feature generation by identifying low-dimensional representations of the initial input features. The initial input features were composed of Morgan fingerprints for compounds and protein sequences for targets, which were embedded to a fixed low dimensional space (i.e. 200 dimensions for compounds and 100 for proteins) using natural language processing (NLP) techniques (i.e. latent semantic analysis and Word2vec). Sub-structures in compounds and amino acid triplets in proteins were treated as words for the embeddings. The system was trained on large-scale ChEMBL bioactivity data by generating training set sizes of 360 835 positive and 93 903 negative examples. These examples were selected using activity measurement values (i.e. IC_50_/Ki values ≤ 1 μM for positive and ≥ 30 μM for negatives). The performance was measured using *k*-fold cross-validation in different settings, and it was compared against random forest as the baseline classifier, where the proposed approach significantly surpassed on the difficult-to-predict setting. The prediction performance was also measured on a test set composed of DUD-E gold-standard data set interactions and compared with another deep learning-based DTI prediction method AtomNet [165]. The elevated performance has indicated effectiveness of the word-embedding approach.

Lenselink *et al.* [246] proposed a PCM deep learning solution to DTI prediction. The training data set was generated using verified bioactivities in the ChEMBL database. Target protein sequences were represented as 169-dimensional feature vectors based on their physico-chemical properties. Compounds were represented by varying lengths of Morgan fingerprints (e.g. 4096, 2048, 512 and 256-dimensional). The interacting target-compound pairs were fed to multi-task DNN to create the predictive models. The performance change was investigated based on multiple criteria such as the length of the fingerprints, input feature utilization approach (i.e. ligand-based against PCM), the depth and the architecture of the DNNs. The performance was compared with the models trained by naive Bayes, random forest, support vector machines and logistic regression classifiers for both ligand-based and PCM approaches, whereever possible. As a result, the DNN models outperformed the models generated using conventional techniques, and the average performance of PCM-based models was slightly higher compared with the ligand-based ones.

SMILES2vec is a recurrent neural network (RNN) deep learning solution to predict the same physical properties of compounds directly using the SMILES representations as the input [247]. The aim here was also similar to their previous study in terms of performing minimal amount of feature engineering and pre-processing for model construction. Recurrent DNNs were used to train the predictive models and Bayesian optimization technique was used to select the best hyperparameters. The performance results of SMILES2vec were compared with the performances of DNNs trained using engineered features. According to the results, SMILES2vec outperformed other methods on regression tasks and underperformed on classification tasks. The results of these two studies indicated the potential of deep learning in extracting relevant properties from the training data even without carefully constructed features, which may render feature extraction and selection applications unnecessary in the future.

In one of the earliest applications of DNNs for DTI prediction, restricted Boltzmann machines (RBMs), which is a two-layer undirected graphical model was employed [248]. An RBM is not considered as a deep architecture since it only contains one hidden layer. However, an individual RBM was generated for each target, and a large network composed of multiple RBMs was implemented as the final model. The main aim in this study was to construct a multidimensional DTI network model by incorporating DTIs from diverse set of compounds and targets with different types of interactions. The interaction types were divided between ligands and receptors into two groups as direct and indirect interactions. The physical binding of small molecule drugs to target proteins was referred to as direct interaction. The indirect interactions corresponded to the effects of the compounds on proteins by means other than direct binding (e.g. changing the expression level of the gene that encodes the target). The interaction type information was incorporated by adding edge properties to their network. Besides, additional models were constructed for predicting drug modes of action (e.g. activation and inhibition). DTI information in the MATADOR and STITCH databases were used for the training and testing of their method, and it was found that the method was able to predict different types of DTIs and drug modes of action with high accuracy. The proposed method was compared with a simple logic-based approach, and it performed better. Finally, new DTI predictions were produced using the proposed method and verified through literature evidence.

DeepDTIs were developed for the prediction of DTIs using deep belief network (DBN), which is constructed by stacking multiple RBMs [85]. In DeepDTIs, targets are not separated into classes according to protein families to train individual models, instead all targets in the training data are pooled to train one predictive model. The training data was composed of DTIs from the DrugBank database (i.e. 6262 DTIs between 1412 approved drugs and 1520 targets). To generate input features, ECFP fingerprints were employed for compounds, and sequence composition descriptors were used for target proteins, and they were all merged to represent drug-target pairs (i.e. a 14 564-dimensional vector for each pair). Experimental drug-target pairs from DrugBank was used to assess the performance of DeepDTIs and to compare it with other ML methods (i.e. Bernoulli naive Bayesian model, decision trees and random forests). The method was also applied to predict the unknown DTIs between all combinations of drug and targets in their training set and the most probable predictions were manually verified through literature-based evidence. Finally, to test the ability of DBN in abstracting the input and generating a more informative representation of the data in each successive hidden layer, the transformed data generated at each layer was used to train a simple logistic regression classification model for the prediction of DTIs. The performance of the LR model increased with the increasing hidden layer depth, which indicated the effectiveness of the approach.

The method ‘AtomNet’ by Wallach *et al.* [165] is one of the earliest applications of CNNs for structure-based VS. The proposed method incorporated both the compound and target features for training by using the 3D structural information of ligand–receptor (i.e. compound–target) complexes. 3D grids placed over the atomic coordinates in the ligand–receptor complexes were used as input to their CNN, where each grid contained numerical structural features such as atom type enumerations and structural protein–ligand interaction fingerprints. Three data sets (i.e. the DUD-E set and two generated data sets: a DUD-E like benchmark set composed of 78 904 active compounds, 2 367 120 inactive compounds and 290 targets and another data set with experimentally verified inactive molecules composed of 78 904 active compounds, 363 187 inactive compounds for 290 targets, both constructed using ChEMBL) were employed to train and validate their method. For the training of the system, targets that have at least one annotated binding site in sc-PDB database were used. The prediction results were compared with two state-of-the-art structure-based VS (i.e. docking) methods using abovementioned data sets, and the described method outperformed the other algorithms with a large margin. This study is significant in terms of indicating that CNNs can be used to model the structural properties of ligand–receptor complexes with a performance better than conventional docking-based approaches.

A CNN architecture with a mixture of PCM and structure-based DTI prediction approach was also proposed [249]. The method takes protein 3D structure information (i.e. the specific binding pocket of the target) along with compound descriptors (i.e. fixed-size 3D structural fingerprints based on learnable atom convolution operations generated from ECFPs) in a pairwise-input format. The insufficiency of current benchmarking data sets for testing structure-based methods was discussed and instead, a new data set generated from DUD-E, PDBBind and MUV data sets was described. The method was trained and tested by this described data set. The method was compared with the state-of-the-art methods (i.e. docking methods and AtomNet: another DNN-based approach), and the models trained with learnt compound features resulted in better performance compared with the models trained with simple ECFPs.

Another CNN-based method for the prediction of chemical properties of compounds such as binding, toxicity and free energy solvation was described by Goh *et al.* [250]. CNN-based techniques are highly used in computer vision with high performance. The focus of this study was constructing predictive models with minimal amount of feature engineering and chemical knowledge. In this method, each compound was represented as an 80×80 pixel sized image based on their 2D drawings, as shown in chemical databases. These images were then fed to the CNN for classification. Three different data sets were obtained from MoleculeNet benchmark database. The first data set was Tox21, which was composed of 8014 compounds labelled as ‘toxic’ or ‘non-toxic’. The second data set was freeSolv data set, including 643 compounds with measured hydration free energies of small molecules. Finally, HIV data set included bioactivity measurements of 41 913 compounds against the inhibition of HIV replication. Two classification models were separately trained using HIV and Tox21 data sets, and a regression model was trained using the freeSolv data set. The results were compared with the results of the models that were trained with conventional ECFP4 fingerprints using multi-task DNNs. The described method slightly outperformed the conventional feature utilization method in HIV and freeSolv data sets and slightly underperformed in Tox21 data set.

A graph convolution deep learning method was described to extract learnable features from the graph representations of compounds (the vertices in the graphs correspond to atoms, and edges correspond to bonds between atoms) and to perform learning using the extracted features for DTI prediction [251]. Several data sets coming from PubChem, Tox21, MUV and DUD-E were combined to achieve 38 million data points. The graph structures of compounds were generated using SMILES representations, and the extracted graphs were fed to the proposed DNN to train the system. The described models were compared with the models trained with multi-task DNN, random forest and logistic regression methods, which were trained using ECFP4 fingerprints. The described method could not outperform the other methods but achieved a comparable performance. Nevertheless, this work stands as a proof of concept that indicates graph convolutions can be a good alternative for employing deep learning for VS with a simple compound feature encoding.

A novel deep-learning architecture ‘iterative refinement long short-term memory’ was developed using graph CNNs, especially for protein targets with low number of training instances [167]. The method allows the learning of sophisticated small molecule features using one-shot learning methodology and yield more reliable predictions when the training data set is small. Training data sets were generated using assay results from three different sources, which were Tox21 challenge data set, SIDER database and MUV data set [173, 187, 241]. Drug-target prediction problem was designed again as a binary classification problem, and multiple models were trained for each target, where inputs were 2 D graph structures of compounds, and outputs were binary variables as active or inactive. One-shot deep learning architecture was combined with iterative refinement long short-term memories and graph convolutions. Graph convolutional features of compounds were used as feature vectors to train neural network models. This novel method was compared with random forest as a baseline classifier. The proposed method obtained significant performance improvement on data sets having low number of training samples compared with the baseline classifier. The models were released as a part of the open-source DeepChem framework (https://github.com/deepchem/deepchem).

According to the ‘deep learning for virtual screening’ studies published so far, DNNs are especially convenient for analysing the relationship between the compounds and targets since the data is high dimensional, and the attributes contributing to molecular interactions are not clearly known [238]. In these studies, the deep models have exhibited elevated DTI prediction performance even with minimal data pre-processing and minimal parameter optimization. In these works, the authors mostly focus on discussing the applicability of deep learning techniques on DTI prediction problem over the architecture and hyper-parameter selections [167, 242, 243], concluding that deep learning has a substantial potential to advance the field of computational drug discovery [163, 239].

Apart from DTI prediction, deep learning techniques are also employed for other drug discovery-related purposes. For instance, Mayr *et al.* developed DeepTox, an ensemble deep learning-based compound toxicity prediction method and won the Tox21 data challenge [241]. Related to this, Maltarollo *et al.* reviewed the applications of various machine learning approaches including DNNs for ADME-Tox (i.e. absorption, distribution, metabolism, excretion and toxicity) prediction [252]. Aliper *et al.* proposed a DNN-based therapeutic effect predictor for compounds, using only the drug-induced transcriptomic profiles in different cell lines as input [253]. In one of the earliest applications of deep learning in drug discovery Lusci *et al.* proposed an ensemble of recursive neural networks to predict the molecular properties of compounds such as the aqueous solubility. The authors developed a web-based tool ‘AquaSol’ for the prediction of the aqueous solubility of compounds, which takes SMILES representations as input [254].

There are several review articles on deep learning applications on the biomedical data [203, 235, 236, 239, 240, 250, 255, 256]. In some of these studies, the authors explained several DNN architectures that has been successfully applied on non-biomedical fields and discussed the current and potential applications on biomedicine [203, 235, 236, 239, 240]. In a few of these review studies, specific applications of DNNs in VS have been discussed as well [239, 256, 257]; however, most of the original research articles on this topic came out just recently (in late 2016 and in 2017), which were not included in these reviews. Apart from the machine learning-based prediction methodologies, some review studies focused on available toolkits, frameworks, databases and representations/descriptors for computational drug discovery [37, 41, 213, 257].

### Evaluation metrics and performance comparison of VS methods

Evaluating the performance of machine learning methods is crucial to be able to assess how well a method performs and to fairly compare the performances of different methods. Here, we demonstrate the most widely used evaluation metrics in the literature, which are precision, recall, F1-score, F0.5-score, accuracy and Matthews correlation coefficient (MCC) (formulations are given below together with quantitative ranges).

(1) $$Precision=\;\frac{TP}{TP+FP}\;Range\;[0,\;1]$$

(2) $$Recall=\;\frac{TP}{TP+FN}\;Range\;[0,\;1]$$

(3) $$F1\;score=\;\frac{2\;\times \;Precision\;\times \;Recall}{Precision+Recall}\;Range\;[0,\;1]$$

(4) $$F0.5\;score=\;\frac{1.25\;\times \;Precision\;\times \;Recall}{0.25\;\times \;Precision+Recall}\;Range\;[0,\;1]$$

(5) $$Accuracy=\;\frac{TP+TN}{TP+TN+FP+FN}\;Range\;[0,\;1]$$

(6) $$MCC=\;\frac{TP\;\times \;TN-FP\;\times \;FN}{\sqrt{\left(TP+FP\right)\;\times \;\left(TP+FN\right)\;\times \;\left(TN+FP\right)\;\times \;(TN+FN)}}\;Range\;[-1,\;1]$$

(7) $$False\;positive\;rate\;(FPR)=\;\frac{FP}{FP+TN}\;Range\;[0,\;1]$$

(8) AUROC=Area under the receiver operating characteristic curve Range [0, 1]

In the equations above, TP, FP, TN and FN represent the number of true positives, false positives, true negatives and false negatives, respectively. Each of these metrics has different properties. For example, precision refers to fraction of the correctly predicted samples (TP) among all positively predicted targets, whereas recall (i.e. TPs rate) denotes the fraction of correctly predicted samples among all truly positive samples. Evaluating the performance of methods using only precision or only recall may result in unrealistic conclusions. For example, if using only precision as the evaluation metric would results in overlooking the high number of FN predictions, since precision does not take FNs into account. The same case is applied for the recall and the FPs. To overcome this issue, F1-score is employed, which is a harmonic mean of precision and recall, to consider both the FPs and FNs. F1-score gives equal weights to precision and recall; therefore, both metrics are treated same. However, in some VS studies, reducing the number of FPs is considered to be an important issue to provide more reliable predictions [70, 71]. For this, F0.5-score is used, where twice the weight is given to precision compared with recall, to minimize number of FP predictions, in other words, to increase the probability of a positive prediction to be a TP. Accuracy measure can be defined as the fraction of correctly predicted samples among all samples in the training data set. Evaluating the system performance based on accuracy may result in high bias, especially when the positive and the negative classes are imbalanced. Considering the VS data, the number of negative samples are usually significantly higher than number of positive samples. For a failing predictive model which classifies all instances as negative (i.e. inactive or non-interacting), the accuracy measure would result in overestimated performance. MCC is another measure which also is a balanced performance calculation metric similar to the F1-score. It was reported that MCC can very well be used for performance evaluation when classes are imbalanced [258]. The main difference between MCC and F1-score is that F1-score does not take TNs into account, whereas MCC does. Therefore, using MCC for performance evaluation can be more convenient, especially when one has a reliable negative training data set. All of the metrics explained above are used to measure the performance of a predictive model at one point (i.e. at a selected prediction score threshold, above which the corresponding compound-target pair is predicted to be interacting/active, and below which they are estimated to be non-interacting/inactive). However, the generalization of the performance over the whole threshold spectrum is also required, especially to fairly compare the performance of multiple methods. The area under the receiver operating characteristic (AUROC) curve (i.e. a 2D plot where the horizontal and the vertical axes correspond to FPs rate and the TPs rate, respectively, drawn considering the performance measures at different arbitrarily selected score thresholds) or the area under the precision versus recall curve (AUPR; i.e. a similar plot where the precision and recall values are used as the two dimensions) are employed for this purpose. It is also important to note that the discriminative power of AUROC diminishes at low FPs rates; as a result, AUROC is usually considered inferior to AUPR. Considering the range of values that can be obtained using these metrics, 1 usually indicates a perfect classifier, and the classifier performance decreases with the resulting measure getting closer to 0. As for MCC where the range is between −1 and 1, the measure of 0 indicates a random classifier and −1 indicates a perfect negative correlation. As a conclusion, the evaluation metrics should be selected based on the nature of the problem at hand. Calculating the performance of different systems using multiple evolution metrics is generally preferred to be able to observe the system behaviour from different perspectives.

In most of the VS studies where a new predictive methodology is developed, the performance of the proposed models is measured using the abovementioned evaluation metrics and compared with the performance of the state-of-the-art methods from the literature. This process provides a general idea about both the biological relevance of the results of the proposed method and its added value over the previously published approaches. Here, we combined the selected performance results from the reviewed deep learning studies and presented them in Table 9. Nearly all of the included works employed a different test data set and used different evaluation metrics; as a result, it is not possible to make a cross performance comparison between the methods mentioned in different studies. However, we included the results of the performance comparison provided in each individual study (i.e. the proposed method is usually compared against a few other methods) in Table 9, which indicates the effectiveness of each approach in a broad way. In other words, performance values given in each row of Table 9 are comparable with each other. According to the reported performance comparison results in Table 9, deep learning-based models usually performed better when compared with shallow (i.e. non-deep learning) machine learning methods; however, in some cases the performance gain is only minor. Random forest classifier is generally the best performing shallow method, and its performance is close to the deep learning-based methods in many cases. Convolutional DNNs, which employ target structure features, perform better than the conventional structure-based VS methods (i.e. docking). There is no consensus regarding the performance comparison between different deep learning-based architectures; nevertheless, multi-task architectures generally perform better than their single-task counterparts. In terms of the input feature utilization, methods that employ both the compound and target features together at the input level (i.e. PCM-based approaches) perform better compared with the ones that employ only compound features.

**Table 9.** Predictive performance results and comparison reported in various deep learning-based VS studies

Article	Evaluation metric	Source of the test data sets and Data set statistics (where available)	Predictive performance results								
			Proposed DNN method^a^	Compared methods							
Dahl *et al.* [238]			**Multi-task DNN^b^**	**Single-task DNN**	**RF^b^**	**Decision Tree Ensembles**					
	AUC^b^	PubChem B: 19 AC: 69 396 IC: 70 331	0.825	0.793	0.783	0.795					

Ma *et al.* [164]			**Multi-task DNN**	**RF**							
	Pearson correlation coefficient	Merck in house set and other data sets T: 15 (Merck) C: 164 024 (Merck) T: 15 (Other) C: 974 795 (Other)	0.496	0.423							

Unterthiner *et al.* [243]			**Multi-task DNN**	**SVM^b^**	**BKD^b^**	**LR^b^**	***k*-nearest neighbour**	**Parzen-Rosenblatt**	**Bayesian Classifier**	**Similarity Ensemble**	
	AUC	ChEMBL T: 5069 C: 743 336 I: 2 103 018	0.83	0.816	0.803	0.796	0.775	0.73	0.755	0.699	

Ramsundar *et al.* [242]			**Pyramidal multi-task NN^b^** **(PMTNN)**	**LR**	**RF**	**Single-Task NN (STNN)**	**Pyramidal Single-Task NN (PSTNN)**	**Max{LR, RF, STNN, PSTNN}**	**Multi-task NN (MTNN)**		
	AUC	PubChem Bioassay B: 128 I: ∼282 000	0.873	0.801	0.800	0.795	0.809	0.824	0.842		
		MUV B: 17 I: ∼15 000	0.841	0.752	0.774	0.732	0.745	0.781	0.797		
		Tox21 T: 12 I: ∼6000	0.818	0.738	0.790	0.714	0.74	0.79	0.785		

Koutsoukas *et al.* [164]			**DNN**	**SVM (rbf kernel)**	**SVM (linear kernel)**	**RF**	***k*-nearest neighbour**	**NB^b^**			
	Mean MCC	ChEMBL T: 7 AC: 7218 IC: 72 082	0.912	0.904	0.861	0.892	0.821	0.764	
Wang *et al.* [244]			**PINNs**	**Bipartite Local Model**	**CS and PD^b^**						
	AUC	sc-PDB T: 836 C: 2710 I: 6830	0.959	0.799	0.858						

Wan *et al.* [245]			**DN** **N**	**RF**							
	AUC	DrugBank D: 2868 T: 3314 I: 9349	0.792	0.686							
	AUC	ChEMBL AI: 156 083 II: 39 857	0.880	0.879							
	AUC	Binding DB AI: 418 577 II: 117 210	0.875	0.855							
	AUC	PDB-Bind AI: 2188 II: 578	0.880	0.763							

Lenselink *et al.* [246]			**DNN PCM^b^**	**DNN QSAR**	**DNN Multi Class**	**LR QSAR**	**SVM QSAR**	**NB QSAR**	**RF QSAR**	**RF Multi Class**	**RF PCM**
	AUC	ChEMBL T: 1, 227 C: 204, 085 I: 314, 767	0.894	0.879	0.89	0.858	0.858	0.679	0.868	0.502	0.845
	MCC		0.610	0.600	0.63	0.572	0.572	0.380	0.630	0.010	0.670

Wen *et al.* [85]			**Deep Belief Network**	**Bernoulli NB**	**Decision Trees**	**RF**					
	AUC	DrugBank D: 1412 T: 1520 AI: 6262 II: 6262	0.916	0.754	0.768	0.910					

Wang *et al.* [248]			**RBMs**	**Logic-based approach**							
	AUC	MATADOR and STITCH D: 784 (MATADOR) T: 2431 (MATADOR) I: 13 064 (MATADOR) D: 598 (STITCH) T: 671 (STITCH) I: 3296 (STITCH)	0.987	0.921							
	AUC (precision vs. recall)		0.896	0.816							
Wallach *et al.* [165]			**Conv. DNN (AtomNet)**	**Smina**							
	AUC	ChEMBL-20 PMD	0.781	0.552							
		ChEMBL-20 in-actives A subset of: T: 290 AC: 78, 904 IC: 2, 367, 120	0.745	0.607							
		DUD-E-30	0.855	0.700							
		DUD-E-102 A subset of: T: 102 AC: 22 886	0.895	0.696							

Gonczarek *et al.* [249]			**Graph Conv. DNN**	**Neural fingerprints**	**AutoDock Vina**	**Smina**					
	AUC	DUD-E T: 102 AC: 22 886 IC: ∼1 million	0.567	0.704	0.633	0.642					
		MUV B: 17	0.474	0.575	0.503	0.503					

Kearnes *et al.* [251]			**Graph Conv. DNN**	**MaxSim**	**LR**	**RF**	**Pyramidal multi-task NN**				
	AUC	PubChem Bioassay B: 128 I: ∼282 000	0.908	0.754	0.838	0.804	0.905				
		MUV B: 17 I: ∼15 000	0.858	0.638	0.736	0.655	0.869				
		Tox21 T: 12 I: ∼6000	0.867	0.728	0.789	0.802	0.854				

Altae-Tran *et al.* [166]			**Iterative refinement LSTM^b^**	**Graph Conv. DNN**	**Siamese one-shotlearning**	**Attention LSTM**	**RF**				
	AUC	Tox21 B: 12	0.823	0.648	0.820	0.801	0.586				
		SIDER 27 side effects	0.669	0.483	0.687	0.553	0.535				
		MUV B: 17	0.499	0.568	0.601	0.504	0.754				

a In the case of multiple DNN methods proposed, one of them is shown under the proposed method column and the rest are given under the group of compared methods.

b Abbreviations: T: target, D: drug, C: compound, AC: active compound, IC: inactive compound, I: interaction, B: bioassay, AI: active interaction, II: inactive interaction, DNN: deep neural network, RF: random forest, AUC: Area under the ROC curve, SVM: support vector machine, BKD: binary kernel discrimination, LR: logistic regression, NB: naive Bayes, CS & PD: chemical substructures and protein domains, PCM: proteochemometric modelling, NN: neural net, LSTM: long short-term memory.

## Discussion and conclusion

In this survey, we focused on the recent machine learning applications in VS with methods, tools, databases and the resources that are used to construct models. First, we defined the terms relevant to the field of VS and the importance of this field for the drug discovery process. We presented examples of VS studies that led to discoveries of novel bioactive compounds and drugs. In the following parts of the study, we described several types of features that are used in VS studies, especially for machine learning applications. We investigated various cheminformatics toolkits and libraries, which can handle different representations of compounds, generate molecular descriptors and carry out basic analyses. We examined currently available chemical structure and bioactivity databases that are employed for data set generation. We also discussed gold-standard data sets that are frequently used to train and test VS models. Subsequently, the two main machine learning approaches, namely, supervised and unsupervised, were discussed with several applications. We examined several VS methods based on the input feature representation (i.e. similarity-based and the feature-based). Along with this, we discussed novel deep learning applications, which outperformed the conventional methods in terms of predictive performance. Our observations and comments about recent VS studies are given below together with future perspectives.

- Today, the prediction performance of even the best conventional VS methods is low. FP hits constitute the main problem in these methods. The precision measure should be considered during the method optimization procedure, since using only accuracy may result in over optimistic evaluations. However, it is also important to state that, FP hits are not considered as a problem in some of the practical applications. Nonetheless, there is room for a great improvement, and the application of novel machine learning methods for VS may remain as a non-trivial task for longer periods of time.
- A significant issue in predictive model development in VS is the training data set construction. In DTI prediction, as in all machine learning applications, training sets should contain reliably labelled data. The labelling is usually a binary procedure, e.g. a certain compound is either interacting with the corresponding target (Label 1) or not (Label 0). However, in reality the interaction is experimentally measured in a continuous scale (e.g. IC_50_ values measured in terms of molarity), and it is not clear what should be the threshold activity value to assume interaction. In most computational research studies, the IC_50_ values of 10 μM or lower are accepted as active. However, in drug development pipelines most candidate drugs that pass the lead discovery and optimization steps have activity values below micromolar concentrations. The reason behind relaxing this threshold to 10 µM in computational studies is that, with more stringent values, the number of data points to be used in training is scarce. Naturally, using relaxed thresholds comes with the cost of noisy training data (e.g. labelling the cases, where the activity is not sufficient, as active). This issue is even more complicated during the selection of negative training data set instances (i.e. the drug-target pairs that are labelled as non-interacting). There is no consensus on what constitutes a sufficient threshold, over which one can assume non-interaction. In different studies, values such as greater than 10, 20, 30, 40 or 100 μM are used. Data point scarcity is even more pronounced in negative training data set selection. Since high IC_50_ values are not desirable, experimentally observed high activity values are often not reported in the literature and in the bioactivity databases. In many cases experimentalists do not even measure the activity after the accepted near border active concentrations such as the 10 μM. In the end, there are few instances to be used as negative training instances. This issue is generally known as the class imbalance problem in machine learning. The widely accepted solution to this problem in the field of DTI prediction is removing the positive instances from all possible combinations of drugs and targets and randomly selecting cases from the remaining set. It is assumed that the ratio of truly active to inactive pairs is so low that random selection would yield a good quality negatives set. However, this is not always guaranteed as knowledge regarding the truly active to inactive ratio is not known. There are also alternative solutions to this problem such as the advanced sampling techniques [259].
- A similar issue is also reflected during the predictive performance calculations in model testing. In most applications, when the tested model predicts an active drug-target pair that was marked as inactive in the validation set, the model is penalized with an FP count. However, there is always a chance that the predicted activity would be true, but not experimentally proven yet (especially when the random selection process is employed in the generation of negative training sets). From a general perspective, the aim of constructing predictive models in the first place is identifying those unknown pairs that are probably interacting. Penalizing models in this sense directs them to predict only those drugs that are structurally similar to the known ones, and this is a common issue associated with a large portion of the conventional VS methods. Various performance metrics have been proposed to tackle this issue by evaluating the performance of predictive models from different angles (this topic is explained and discussed in the section entitled: ‘Evaluation Metrics and Performance Comparison of VS Methods’). One of the solutions proposed for negative test instance selection problem is employing decoys, which are compounds that have similar physico-chemical properties but different topologies compared with the known active compounds for the selected targets. These decoy molecules are inactive against the corresponding targets; as a result, they can be used in negative test sets to accurately assess the performance of the models regarding the FPs. The issue with decoy sets is that they are available for just a few targets. Decoy data sets are explained in the section entitled ‘Gold Standard Data sets for VS’.
- The ATC Classification System provides valuable information for the classification of drugs in terms of their therapeutic effect and their pharmacological and physico-chemical properties. Assigning an ATC code to a compound requires curation efforts, as a result, only approved and experimental drugs have ATC code annotations. Large-scale prediction of ATC codes for all compounds recorded in chemical databases can help to identify the roles for these compounds. In addition, predicting new ATC codes for known drugs can be used to aid drug repositioning. Currently, there are only a few ATC code prediction studies in the literature, most of which have been proposed in the past few years. We expect to see more studies of this kind in the future.
- Deep learning techniques have shown significantly better performance for DTI prediction compared with the conventional machine learning methods. As a result, we expect a significant shift, not only in VS but also in the drug discovery field in general, towards utilizing novel deep learning-based architectures in the near future. Besides, the flexibility of deep learning architectures allows researchers to model DTIs in various ways, each of which may have specific advantages.
- Most of the deep learning-based studies so far emphasized the potential and applicability of DNNs for the development of efficient VS methods; however, there are no public production pipelines to predict and publish large-scale DTIs. Considering the current availability of the chemical structures and bioactivity information in public databases, which is required for constructing such pipelines, we expect to see DNN-based large-scale analyses and novel web-services presenting their results in the near future.
- Considering the problem of noise in the training data (especially in the negative training sets), which was discussed above, one interesting point is that, deep learning methods have been reported to be robust against the noise in the training data, not only for negatives but also for positives. It would be interesting to observe the situation in DTI data. If DNN models can be stable against errors in the negative training data, the process of training data preparation may become trivial. Nevertheless, we expect to see new approaches in the literature to generate more reliable negative training data sets, especially for conventional machine learning techniques. One approach can be utilizing the hierarchical structure of ATC classification system, as similar ATC codes indicate similar functions and targets.
- In the literature, it was indicated that the prediction performance of computational methods was highly dependent on the targets. Therefore, target-specific machine learning and feature selection methods can be investigated more to enhance accuracy of predictions. To the best of our knowledge, there is no study in the literature that employs target-specific feature selection. Conversely, it has been stated in the literature that deep learning techniques do not require hand-crafted features generated with the application of feature extraction and selection methods, and simple encodings of the raw input data is sufficient for the models to produce high-quality predictions. This is because of the ability of capturing the structures hidden in the data by building complex features in a hierarchical manner. We expect to see additional investigative studies to identify the status in the DTI data.
- A trend in DTI prediction that we expect to become more popular in the near future is integrating large-scale omic data (e.g. transcriptomics, interactomics, epigenomics, metabolomics and functional genomics) at the input level, to increase both the quality and the coverage of DTI predictions. Conventionally, known bioactivities are used along with the structural attributes of compounds and/or target proteins to model the DTIs. However, the recent accumulation of the omic data presents opportunities for the identification of the unknown parts of the DTI space. The expected contribution of the omic approach mainly comes from integrating different types of features in an ensemble/hybrid setting, where different features complement each other to produce a more complete picture. Since components of the omic data have different structures (e.g. interactomic data mostly define the pairwise relations between proteins, transcriptomic data displays quantitative measurements in terms of how the expression of genes change under different conditions), generation of the feature vectors with the standardization of the information have critical importance.
- A significant factor affecting the performance of conventional machine learning models (i.e. non-deep learning-based techniques) is the quality of the input feature representation. The constructed feature vectors should accurately reflect various properties of compounds and/or targets that play roles in their interaction. Usually, manual feature engineering is performed for this purpose, where the aim is to generate or select the most representative features for the DTI. Generating and manually testing these features is a tedious and an intensive process, and automated feature selection methods are employed for this purpose. Feature selection is especially important for methods that integrate large-scale omic data, since the raw feature vectors produced by ensemble methods are usually quite large, which increase the computational complexity and hinders the optimal training of the systems. With the increasing interest in incorporating omic data for DTI prediction, we expect the feature selection procedures to gain even more importance. For details regarding the feature selection process, please refer to the section entitled ‘Feature Selection’ in the supplementary material.
- It is reported in the deep learning literature that, as long as the models are trained successfully, DNNs are capable of extracting the information hidden in the data even without sophisticated input features. The so called end-to-end learning approach states that the multiple steps in a predictive procedure, such as the data pre-processing, representative feature vector generation and the prediction post-processing, can be automatically accomplished by the predictive network model itself. For this purpose, usually a task-specific complex architecture is required to be constructed by an expert. However, once the system is accurately constructed, it is easy to accomplish prediction tasks. The features fed to an end-to-end learning system can be as simple as one-hot-encodings of biomolecular sequences (i.e. *n* by 20 matrices filled with 0s and 1s, where *n* represents the sequence length, each of the 20 columns represent a unique amino acid and 1s in the matrix show the presence of the corresponding amino acid at that position). DNNs are especially suitable for end-to-end learning approaches because of their modularity and complex nature. End-to-end learning-based DNN models are gaining popularity lately, and we expect to see successful applications in VS in the near future.
- A computational drug discovery topic that is rapidly gaining popularity is the machine learning-based *de novo* drug design, proposed as a solution to the problem of reduced diversity of drug candidate compounds offered by the conventional predictive models. The aim behind the *de novo* drug design is identifying novel drug candidates that are structurally significantly different compared with the ones already in the market (or the ones in the development phase). Classical *de novo* drug design methods follow a rather manual procedure, where the researcher carries out a series of intensive computational processes such as docking and/or molecular dynamics simulations. The desired molecular properties are extracted and combined with a fragment-based approach, to computationally generate novel molecules. Various types of molecular properties can be used for this process such as the physico-chemical properties of compounds and the 3D structures of targets, including the binding site information. At the end of a *de novo* drug design process, the finalized computationally generated ‘non-existent’ compounds are chemically synthesized and employed in bioassays to verify the predicted interactions against the corresponding targets. The directed approach used in classical *de novo* drug design produces reliable results, but the experiments are time consuming, and the output is small scale. In machine learning-based *de novo* drug design, first, the desired structural properties of molecules (i.e. the constraints) are identified (e.g. molecular attributes of a hypothetical compound that would yield a high affinity to bind to a specific target, and the properties that are required for the chemical synthesis and stability) using ordinary labelled interaction data, similar to the classical *de novo* drug design. After that, different varieties of these molecules, which harbour the identified properties, are computationally generated with a randomization factor to increase diversity. This job is accomplished by generative models. This is followed by constructing the feature vectors for the computationally generated molecules and feeding them to an interaction prediction model, as the input. The output score obtained for each computationally generated molecule, which indicates the probability of interaction, is fed back to the generative model to create additional varieties, and the process continues in an iterative manner until the optimal point (i.e. the maximum prediction probability) is achieved. Lately, DNNs are employed to construct both the generative and the testing models in a fully automated manner [260]. It is also possible to construct just one model for both the generation and the testing jobs, where the produced signals are transmitted back to the initial (i.e. generative) layers using the backpropagation algorithm. We expect that, with the employment of DNN-based models, the field of *de novo* drug design will start to produce truly novel drug candidates in the near future.
- For some of the traditional ML methods, such as the SVM, low amount of training instances is often sufficient; however, the training data should be error-free to generate a high-performance predictive model. It is generally the opposite for DNNs, as successful applications of DNN models are usually trained with a large number of instances even though they contain high error rates in some cases. Although finding labelled data in this scale is not a problem in computer vision and NLP, it usually is a difficult task considering the biological data. Employment of the large-scale bioactivity data from public bioassay databases (e.g. ChEMBL, PubChem and BindingDB) is an option that has already found applications in the literature. Apart from that, we expect to observe training data set enrichment implementations for the deep learning applications on biomedical data.
- One of the most important challenges regarding the development of novel deep learning-based methods is still the computational complexity. Especially, system training processes using large-scale data requires extreme amounts of computational power. There is a growing field of research on novel algorithmic approaches to reduce the complexity of DNN-based techniques without compromising the prediction performance. Apart from that, GPU-based technologies are emerging lately to provide affordable high-performance computational equipment to scale to the level of big data. The big-tech companies such as Google, IBM, Microsoft and Nvidia started experimenting with deep learning libraries, frameworks and related tools to provide open-access data analysis instruments to the public (e.g. TensorFlow, Caffe, Theano, Torch, cuDNN and Apache Spark). However, there is still some time before these systems (in terms of both hardware and software) become easily affordable, fully functional and available to the non-specialist.

## Supplementary Material

bby061_Supp

## Notes

**Ahmet Sureyya Rifaioglu** is a PhD student in Computer Engineering program. He is a computer scientist by training and is working on machine learning-based predictive method development in the fields of bioinformatics and computer science.

## Notes

**Heval Atas** is a PhD student in Medical Informatics program. She is a molecular biologist by training and has previous experience on *in vitro* bioassays for drug discovery. She is currently working in the fields of bioinformatics and computational biology.

## Notes

**Maria Jesus Martin** is a PhD and a group leader of Protein Function Development Team at EMBL–EBI. Martin’s team comprises software engineers and bioinformaticians and provides the bioinformatics infrastructure for the databases and services of the Universal Protein Resource (UniProt) and study novel methods for protein annotation.

## Notes

**Rengul Cetin-Atalay** is an MD PhD and a group leader of CanSyL and faculty member of the Department of Health Informatics. She is a medical doctor by training and is working on cancer research and drug discovery. Her team comprises both wet-lab and dry-lab researchers to study the systems perspective of liver cancer and potential novel treatments via experimental drug discovery.

## Notes

**Volkan Atalay** is a PhD and a faculty member of the Department of Computer Engineering and is a computer scientist by training. He is working on machine learning method development and applications on biological data, in the field of bioinformatics, specifically on the functional annotation of proteins.

## Notes

**Tunca Doğan** is a PhD and an adjunct faculty member at the Department of Health Informatics, a member of CanSyL, research associate at the Protein Function Development Team at EMBL–EBI, working on biological data analysis, machine learning and data mining-based predictive method development, in the fields of bioinformatics and computational biology, specifically on protein sequence analysis and computational drug discovery.
