# Supplementary material for: Recent applications of deep learning and machine intelligence on in silico drug discovery: methods, tools and databases
Source: Brief Bioinform. 2018 Jul 31;20(5):1878–912. doi: 10.1093/bib/bby061 (PMC6917215; doi:10.1093/bib/bby061)
Supplement: bby061_Supp [file bby061_supp.docx]

**SUPPLEMENTARY MATERIAL**

# DESCRIPTORS AND FEATURES FOR VIRTUAL SCREENING

## **Compound Descriptors and similarity calculation**

***Simplified molecular-input line-entry system (SMILES)*** is a line notation that represents compounds using ASCII characters [1]. SMILES can be used to encode 2D structures of compounds into short-length string values known as SMILES strings. Atoms, bonds, aromaticity as well as branching properties are represented by special characters (for example, charges (“+”, “-”), single bond (“-”), double bond (“=”)) based on definitive rules. It is possible to generate 2D structures from SMILES and vice versa. SMILES strings are structured as human-readable notations and is one of the most popular line notation systems.

***SMILES Arbitrary Target Specification (SMARTS)*** is a line notation to represent substructures and patterns in compounds with the aim of searching substructures quickly in SMILES databases [2]. SMARTS is an extension of SMILES therefore; SMILES rules also apply to SMARTS. The SMARTS notation has additional logical operators and more specialized characters (for example, wild card searching) that allows to search substructures based on more flexible criteria. For example, hydroxyl substructures can be searched by entering a [OX2H] SMARTS string against a SMILES database.

***IUPAC International Chemical Identifier (InChI)*** is another line notation solution to represent chemical structures [3]. Each compound is represented by a unique InChI string using a well-defined algorithmic procedure. InChI strings can also be converted into InChIKey, which is the 27 character long hashed version of InChI strings. The aim of generating InChIKey is to enable quick search in InChI databases with low collision rates.

The key features that differentiate InChI notations from SMILES are that;

- in SMILES, the same compound may have different SMILES notations whereas each InChI string represents a unique compound,
- SMILES is structured as human-readable notation, however InChI string is only machine readable and
- SMILES is a proprietary project whereas InChI is an open source project.

We can divide fingerprints into 2 main categories such as *2D* and *3D structure-based fingerprints* [31,32].

***2D structure-based fingerprints*** constitute three main categories:

- *Substructure keys-based fingerprints* are created based on the presence or absence of predefined substructures in compounds. MACCS fingerprints is a popular example of substructure keys-based fingerprints [4].
- *Path-based fingerprints* are created by extracting linear paths from the 2D structure of compounds. The length of the path can be defined by users. DayLight fingerprints and FP2 fingerprints are two examples of path-based fingerprints [5,6].
- *Circular fingerprints* are created by extracting patterns around atoms of the input compound, which is based on a specific bond diameter/length. Extended Connectivity Fingerprints (ECFP) are the most widely used circular fingerprints [7]. For example, ECFP4 are used to extract patterns of molecules for bond diameter of 4.

***3D structure-based fingerprints*** constitute two main categories:

- *Geometrical fingerprints* are calculated from the 3D coordinates of atoms of compounds to describe structural and geometrical properties such as the shape-based or polar/apolar surface properties.
- *Pharmacophore fingerprints* represent pharmacological and binding properties of compounds such as hydrophobicity or aromaticity.

In general, similarity and distance calculation for real valued feature vectors are also one of the mostly used operations in machine learning studies. Some of the available similarity metrics are given in Table S.1. Other types of similarity measures that are used in virtual screening and machine learning can be found in [5,8,9].

**Table S.1.** Commonly used similarity measures and distance metrics.

| **Metric** | **Binary/Real Valued** | **Formula** | **Range** | **Proximity** |
| --- | --- | --- | --- | --- |
| Tanimoto (Jaccard) | Binary | $\frac{c}{a+b+c}$ | 0.0 – 1.0 | Similarity measure |
| Manhattan | Binary | $\frac{a+b}{a+b+c+d}$ | 0.0 – 1.0 | Distance metric |
| Dice | Binary | $\frac{2.0*c}{a+b+2c}$ | 0.0 – 1.0 | Similarity measure |
| Cosine | Binary | $\frac{c}{\sqrt{\left( a+c \right)*(b+c)}}$ | 0.0 – 1.0 | Similarity measure |
| Euclidean | Binary | $\sqrt{\frac{c+d}{a+b+c+d}}$ | 0.0 – 1.0 | Distance metric |
| Pearson | Binary | $\frac{\left( c*d \right)-(a*b)}{\sqrt{\left( a+c \right)*\left( b+c \right)*\left( a+d \right)*(b+d)}}$ | -1.0 – 1.0 | Similarity measure |
| Hamman | Binary | $\frac{\left( c+d \right)-(a+b)}{a+b+c+d}$ | -1.0 – 1.0 | Similarity measure |
| Manhattan | Real Valued | $\sum_{k=1}^{l} \vert x_{il}- x_{jl}\vert$ | 0.0 – ∞ | Distance metric |
| Euclidean | Real Valued | $\sum_{k=1}^{l} {(x_{il}- x_{jl})}^{2}$ | 0.0 – ∞ | Distance metric |
| Cosine | Real Valued | $\frac{\sum_{k=1}^{l} x_{il}*x_{jl}}{\sqrt{\sum_{k=1}^{l} x_{il}^{2}}*\sqrt{\sum_{k=1}^{l} x_{jl}^{2}}}$ | -1.0 – 1.0 | Similarity measure |
| Mahalonobis | Real Valued | $\sqrt{{(\vec{x_{i}}-\vec{x_{j}})}^{T}*S^{-1}*(\vec{x_{i}}-\vec{x_{j}})}$ | 0.0 – ∞ | Distance metric |

Given two compounds, compound A and compound B, and their fingerprints D_A_ and D_B_: a is the number of dimensions set to 1 in D_A_ but not in D_B_. b is a is the number of dimensions set to 1 in D_B_ but not in D_A_. c is the number of dimensions set to 1 in both D_A_ and D_B_. d is the number of dimensions set to 0 in both D_A_ and D_B_. For real valued metrics, x_ik_ stands for i^th^ examples k^th^ dimension. l is the number of dimensions. S stands for covariance matrix.

## **Target Protein Descriptors**

1. ***Descriptors based on sequence composition*** reflect occurrence frequencies of different amino acid combinations in a protein sequence [10].

- *Amino Acid Composition(AAC)* describes the frequency of each amino acid type in a protein sequence, which computes 20 descriptor values in total.
- *Dipeptide Composition (DC)* describes the frequency of each two amino acid combinations in a protein sequence, which computes 20x20 = 400 descriptor values in total.
- *Tripeptide Composition (TC)* describes the frequency of each three amino acid combinations in a protein sequence, which computes 20x20x20 = 8000 descriptor values in total.

1. ***Descriptors based on physicochemical properties*** describe protein sequences in terms of a combination of physical and chemical properties of amino acids such as hydrophobicity, van der Waals volume, polarity, polarizability, charge, secondary structure, and solvent accessibility.

- *Autocorrelation* describes the correlation levels between protein sequences based on the distribution of structural and physicochemical properties of amino acids including hydrophobicity scale, average flexibility index, polarizability parameter, free energy of amino acid solution in water, residue accessible surface areas, amino acid residue volumes, steric parameters and relative mutability, which are derived from AAindex database. There are three types of autocorrelation based descriptors each of which have 240 descriptor values; *Normalized Moreau-Broto Autocorrelation*, *Moran Autocorrelation*, and *Geary Autocorrelation.* They differ in terms of the variable used for measurement. While the property values themselves are used in normalized Moreau-Broto autocorrelation as the basis for measuring correlations, their deviations from the average values are used in Moran autocorrelation, and their square-differences are used in Geary autocorrelation [10,11].
- *Composition(C)* gives the percent frequency of each class of physicochemical properties of amino acids for a given protein sequence, where each of properties are represented with three classes. Hence, it computes 3x7 = 21 descriptor values as total for seven physicochemical properties [12].
- *Transition(T)* gives the percent frequency of successive changes of a particular physicochemical property from one class to another class within a protein sequence. Since there are three classes for each property, possible transition number is also three (Let’s assume classes for a property are 1,2,3. Therefore, possible transitions in the sequence are “(1 to 2) or (2 to 1)”, “(1 to 3) or (3 to 1)”, and “(2 to 3) or (3 to 2)”). Hence, it computes 3x7 = 21 descriptor values as total for seven physicochemical properties [12].
- *Distribution(D)* describes five distribution patterns for each class of a particular property based on chain lengths where the first, 25%, 50%, 75%, and 100% of amino acids for that value are located. Hence, it computes 5x3x7 = 105 descriptor values as total for seven physicochemical properties [12]. (For a detailed example about calculation of C-T-D descriptors, see paper [12]).
- *Conjoint Triad (CTriad)* describes the frequency of each three amino acid combinations in a protein sequence based on a 7-letter reduced alphabet format of 20 amino acids ({A,G,V}, {I,L,F,P}, {Y,M,T,S}, {H,N,Q,W}, {R,K}, {D,E}, {C}) according to their dipoles and volumes of the side chains, which computes 7x7x7 = 343 descriptor values in total [13].
- *Sequence-Order-Coupling Number (SOCN)* reflects the indirect effect of the protein sequence order by calculating the coupling factor according to the physicochemical distance between coupled residues based on the Schneider–Wrede distance matrix [14] that is derived from hydrophobicity, hydrophilicity, polarity, and side-chain volume properties of amino acids. *n*th-rank SOCN represents the coupling mode between all the *n*th most contiguous residues that express sequence order effect in a considerable extent especially when *n* reaches a certain amount -generally set as 30- [15]. In addition to the Schneider–Wrede distance matrix, the Grantham chemical distance matrix [16] can be also used that is computed from composition, polarity, and molecular volume properties of amino acids [17].
- *Quasi-Sequence-Order Descriptors (QSO)* also reflect the indirect effect of the protein sequence (quasi-sequence-order effect). The first 20 QSO descriptors are generated for each amino acid type based on the normalized occurrence of each amino acid type and SOCNs multiplied with a weighting factor. Additional 30 QSO descriptors are also generated using same parameters with a modified formulation [10].
- *Pseudo Amino Acid Composition (Pse-AAC)* describes amino acid composition without losing the sequence-order information in a protein sequence by combining 20 components of conventional amino acid composition with a set of discrete sequence correlation factors computed from average values of three amino acid properties, which are hydrophobicity value, hydrophilicity value and side chain mass. It is also called “type 1 pseudo amino acid composition” [18,19].
- *Amphiphilic Pseudo Amino Acid Composition (Am-Pse-AAC)* also describes amino acid composition without losing the effect of the sequence-order of a protein like *Pse-AAC*. In *Am-Pse-AAC*, the sequence-order correlated factors are computed from hydrophobicity and hydrophilicity indices of amino acids which provides to keep the distribution of amphiphilic amino acids along the protein chain. It is also called “type 2 pseudo amino acid composition” [19,20].
- *Z-scale descriptors* describe amino acid variations in a protein sequence by five scales that are determined based on traditional principal component analysis (PCA) of 26 physicochemical properties of 87 amino acids, which represent hydrophobicity/hydrophilicity (z1), steric/bulk properties and polarizability (z2), polarity (z3), and electronic effects (z4 and z5). Amino acid variations at certain positions are determined from multiple sequence alignment of specific regions (i.e. ligand-binding sites) of the interested protein(s). This descriptor set is suitable for same or close protein families where homology level is high [21].
- *Molecular Surface-Weighted Holistic Invariant Molecular (MS-WHIM)* reflects steric and electrostatic 3D properties of 20 natural amino acids -based on point coordinates of their water accessible surface calculated either in extended conformation or as a weighted average among members of Schrauber’s rotamer library- that are derived from PCA applied on the MS-WHIM description matrices of 36 statistical MS-WHIM indices. Based on results of PCA, only the scores associated with the first three principal components are considered as MSW-scores in the final descriptor set and represented as 3D-descriptors [22].
- *Vectors of Hydrophobic, Steric and Electronic properties (VHSE)* reflects two hydrophobic, two steric and four electronic properties of amino acids that are reduced from independent families of 18 hydrophobic, 17 steric and 15 electronic properties of 20 natural amino acids as a result of PCA. Applying PCA separately for different categories of physicochemical properties instead of computing a data matrix in the form of their linear combinations as in z-scales allow to keep definite physicochemical meanings of amino acids [23].
- *Factor Analysis Scales of Generalized AA Information (FASGAI)* reflects 335 physicochemical properties of 20 natural amino acids -derived from AAindex database- that are processed by a factor analysis to produce a set of factors describing correlated variables in each set which will summarize the complete set of 335 properties. Generated factor sets are then reduced into 6 factors by PCA accounting for an 83.5% variance as total, which mainly represent hydrophobicity, alpha and turn propensities, bulky properties, compositional characteristics, local flexibility, and electronic properties of protein sequences [24].
- *Protein Fingerprint (ProtFP)* reflects 58 physicochemical properties of 20 natural amino acids -derived from AAindex database- that are processed by recursive elimination of the most co-varying properties from a large set of indices. Two types of ProtFP descriptor sets are generated at the final. The first set (ProtFP (PCA3), ProtFP (PCA5) and ProtFP (PCA8)) is based on three principal components (3,5,8) of the remaining indices as a result of PCA that allows to quantitative comparison of amino acids. The second set (ProtFP (Feature)) is based on a hashing approach of all indices per amino acid that results in a single feature for each amino acid [25].

1. ***Descriptors based on similarity measures*** utilize similarities between proteins via sequence or structural alignments, based the idea that similar targets may interact with similar compounds.

- *BLOck SUbstitution Matrix (BLOSUM)* is a substitution matrix used to align protein sequences and score their similarities. It is based on percent clustering of aligned segments in blocks that are obtained from conserved regions of proteins and it is independent from evolutionary distance [26].
- *Point Accepted Mutation (PAM)* is also a substitution matrix used to align protein sequences and score their similarities. Contrary to BLOSUM, it is based on the estimation of mutation rates in closely related proteins that is derived from point accepted mutations [27].
- *The Needleman-Wunsch Score* is a dynamic programming based similarity measure that computes sequence identities between two proteins. As a global alignment technique, it attempts to align all residues between two sequences. Hence, it is useful to catch homology between evolutionarily closely related sequences [28].
- *Normalized Smith-Waterman Score* is another similarity measure as a normalized version of Smith-Waterman algorithm that computes similarities between two proteins. Smith-Waterman algorithm is also a dynamic programming based algorithm, which is used for local alignment of two sequences that considers local similarities between them instead of whole sequence similarity like global alignment. Therefore, it is mainly used to search common sequence motifs between dissimilar sequences [29].
- *Position-Specific Scoring Matrix (PSSM)* is a widely used scoring matrix that represents evolutionary conservation of amino acids in a protein sequence. It is derived from a set of protein sequences that are previously aligned. It can be generated using PSI-BLAST (Position Specific Iterated BLAST) program [30].
- *Substitution Matrix Representation (SMR)* allows to represent proteins as an Nx20 matrix based on a substitution matrix such as BLOSUM, where N is the length of a given protein sequence [31].
- *Root Mean Square Deviation (RMSD) Score* is the most popular but least representative score used to measure structural similarity between two proteins based on the superimposition of atomic coordinates. One of the main disadvantages of RMSD is that it cannot differentiate distinct parts of structures and evaluate them in the same manner, i.e. positional variations in flexible regions such as loops cause a high RMSD score even if other parts between two protein structure is similar. Another disadvantage is that RMSD distribution depends on the protein length, which reduces reliability of the method when comparing proteins with different sizes Generally, RMSD values less than 2.3 Å implies high similarity. However, RMSD measurements are not indicative enough since they are largely affected from flexible regions [32].
- *Global Distance Test (GDT) Score* identifies the largest sets of superimposable residues, which do not have to be contiguous, by calculating distance between Cα pairs of model and reference structure within a defined distance threshold (1 Å, 2 Å, 4 Å, and 8 Å). It prevents being affected from local variations since it evaluates superimposed substructures only. Moreover, it is used in Critical Assessment of Techniques for Protein Structure Prediction (CASP) experiments for the quality assessment of predicted structures. It calculates the percentage of maximized set of residues for each distance cutoff and the average of these percentages becomes the final score, which is called GDT_TS (total score). The score is between 0-100 and higher scores mean more accurate prediction of structure [33]. However, the accuracy of the results is affected by protein size [34].
- *MaxSub Score* is similar to GDT in terms of that it also aims to find the largest subset of superimposed residues by calculating distance between Cα atoms of template and model structure within a given threshold. Differently from GDT, MaxSub uses a single cutoff value for distance, which is 3.5 Å. It normalizes the size of the largest set of residues for this threshold and generates a score -derived from Levitt and Gerstein formula- between 0 and 1, where the model accuracy increases with the increase of this score [35]. However, it is also size dependent, i.e., while a score indicates a significant alignment for a large protein in size, the same score may correspond to a random alignment for a smaller protein [34].
- *Template Modeling Score (TM-score)* is also a structural similarity measure as an alternative tomethods mentioned above. Unlikethem, it is not dependent on the size of the protein. Therefore, it is more reliable for the comparison of protein structures that vary in length. Moreover, it measures global fold similarity that makes it less sensitive to local variations in protein structures. It ranges between 0 and 1, where higher scores mean higher similarities. Scores less than or equal 0.17 correspond to random structure pairs [32,36].
- *Match Score* is a structural similarity measurement implemented in SiteEngine tool that represents the percentage of the query binding site features matched on the interested protein structure. It provides a normalization of the total score of the predicted binding site (called Search_Score) with respect to the total score of this binding site when it is searched in its native protein (called Native_Score). This score is used for the ranking of the predicted binding site matches. If it is used to cluster binding sites, threshold values should be in the range of 28-30 [37].
- *PS-score* is a pocket similarity measurement introduced in APoc. It is determined according to positions of Cα and Cβ atoms, as well as chemical similarity of aligned residues. It can handle different sizes of pockets between 10-100 residues. Moreover, PS-score is less sensitive to conformational changes when compared with a scoring function that considers all heavy atoms. It is also possible to detect similar pockets in proteins which are not similar in terms of sequence. It ranges from 0 to 1, where higher scores correspond to more similar pockets. For comparison of pockets with similar lengths, scores higher than 0.4 are statistically significant with *P* < 1x10^-3^ [38].
- *eMatchSite Score* is a protein pocket similarity measurement implemented in eMatchSite that represents the probability of two pockets bind similar ligands. It is calculated using support vector machines algorithm based on following features; pocket RMSD, average residue-level scores, chemical correlation, physicochemical properties, and geometric hashing. It is in the range of 0-1, where higher scores correspond to higher similarities [39].
- *G-LosA Alignment Score (GA-Score)* is a size-independent local structural similarity measurement based on seven different chemical features (CFs) of amino acids. These CFs are hydrogen bond donor (HD), hydrogen bond acceptor (HA), hydroxyl group (OH), positively charged atom (PC), negatively charged atom (NC), aromatic ring (AR), and aliphatic hydrophobic group (AL). It is between the range of 0-1, where the average score for random local structure pairs is 0.49 and scores above 0.59 are statistically significant with *P* < 5x10^-2^ [40].
- *Ontological Annotation Semantic Similarity* represents similarities between proteins based on semantic similarity scores calculated according to their overlapped ontological annotations. As an ontological annotation, Gene Ontology (GO) terms describe biological roles of genes and proteins in a controlled way at three different levels: molecular function, biological process and cellular component. Hence, GO semantic similarity is measured according to common activities or functions shared between proteins [41].

1. ***Descriptors based on topological properties*** describe amino acids according to atom-connectivity indices generated from molecular graphs [42].

- *Topological Scales (T-scales)* reflect topological features of amino acids based on five most representative principal components derived from PCA of 67 common topological descriptors of 135 amino acids. They are only generated from atom-connectivity related manners in molecular 2D topological map and do not require molecular 3D conformations [42].
- *Structural Topology Scales (ST-scales)* mainly reflect constitutional, topological, geometrical, hydrophobic, electronic, and steric properties of the amino acids based on eight most representative principal components derived from PCA of 827 structural variables of 167 amino acids, which require molecular 3D conformations as opposed to T-scales [43].

1. ***Descriptors based on g******eometrical characteristics*** describe structural characteristics of proteins related to shape, size, atomic positions in space etc., mainly including residue-residue contacts, bond lengths, bond angles and torsion angles between atoms of residues, secondary structures, flexibility, and solvent accessibility of proteins.

- *Descriptors based on distances between protein residues* consider residue-residue contacts of a protein that are close enough to each other based on a specified threshold. As a kind of residue distance based descriptors, *Local Descriptors of Protein Structure (LDPS)* are a collection of residue contacts of proteins that are centered around a particular amino acid in a protein structure. They are determined based on their closeness to the central amino acid. If the distance between C_α_atoms of a residue and the central residue is less than 6.5 A^o^, this residue and its four neighbor residues are added to the descriptor set. Each generated descriptor is labelled with an identification code that represents PDB id, chain id, domain information based on ASTRAL nomenclature and the position of central residue of a protein (e.g. 1e43a2#231 is the descriptor generated from protein with PDB id “1e43”, chain “a”, domain “2” and central residue at position “231”) [44].
- *Backbone Geometry and Conformation* represents measurements of bond lengths and bond angles (backbone geometry), and torsion angles -also known as φ(phi)/ψ(psi), dihedral, or rotation angles- according to x,y,z atomic coordinates of proteins. Torsion angles determine whether a protein is in *cis* form or *trans* form (backbone conformation) [45]. It is possible to identify secondary structure elements of a protein from the distribution of torsion angles in the protein structure by generating a Ramachandran plot [46].
- *Flexibility* represents the fluctuation ability of residues in a protein that is determined according to the displacement of atomic positions of residues. Flexible nature of proteins makes them dynamic entities that facilitates the binding process by affecting their molecular interactions within/among cells. Flexibility of a protein is mainly determined based on two approaches including B-factors and the annotations of the disordered residues (residues in highly flexible, unstable regions) [47,48].
- *Solvent Accessibility* represents the surface area of a protein that is accessible to a solvent which provides to determine whether a residue in a protein is buried or exposed to the solvent. The accessible surface area is typically traced out by rolling the center of a solvent molecule as a probe sphere over the protein surface that is first implemented by Lee and Richards in 1971 [47].

1. ***Descriptors based on functional sites*** describe certain functional characteristics of proteins that can be responsible for the interactions with other molecules such as proteins, small molecules and nucleic acids.

- *Protein Domain Profiles* represent conserved regions annotated on the protein sequence or structure that have a structural or functional importance. Descriptors reflecting protein domain profiles are generally represented as binary vectors that define a protein based on the presence (1) or absence (0) of domains retrieved from specific databases such as Pfam [49].
- *Binding Pockets and Cavities* represent voids, tunnels and channels on the surface or in the interior of a protein that are considered as potential binding sites for ligands and other molecules [50]. The identification of these sites is crucial for molecular docking studies and high-throughput virtual screening to increase predictive power for identification of drug candidates. It is also important for the alignment-dependent descriptor sets that are applied to binding site residues instead of whole protein sequences due to feasibility problems [51].

*- Fuzcav* is a cavity descriptor representing druggable protein-ligand binding sites as a vector of 4833 integers that includes counts of pharmacophoric feature (H-bond acceptor / donor, positive / negative ionizable, aromatic, aliphatic) triplets from the Cα atomic coordinates of binding-site-lining residues which are retrieved from sc-PDB. It can be used to measure local binding site similarities among different targets without aligning them [52].

*- Fingerprints for Ligands and Proteins (FLAP)* also describes binding site similarities based on alignment-free quantification of binding sites by identifying specific target locations where molecular interactions are energetically favorable (energy minimum points) from GRID molecular interaction fields and converting them into four-point pharmacophore fingerprints [53].

# LIBRARIES AND TOOLKITS FOR VIRTUAL SCREENING

## **Tools & Libraries for Compounds**

***RDkit*** is an open source cheminformatics toolkit that provides various implementations for cheminformatics studies such as algorithms for generating the molecular fingerprints, molecular structure searching, and 2D to 3D structure constructions [54]. RDKit can handle several types of representations of compounds such as SMILES and InChI. It also accepts different file types for targets such as PDB and FASTA formats. RDKit can be used with Python, Java, C++ and C# programming languages.

***Open Babel*** is another open source toolkit which provides several functionalities such as the generation of several types of molecular fingerprints, conversion between various chemical formats, structure and substructure searching based on graph isomorphism, organic chemistry tools [55,56]. OpenBabel can be used through Perl, Python, C++ and Mono.

***Dragon*** is a commercial product for the calculation of various types of molecular descriptors and chemical structure analysis [57,58]. Dragon also includes implementations of basic statistical analysis methods such as principal component analysis, pair-wise correlation calculations to analyze produced molecular descriptors.

***Daylight Toolkit*** is a proprietary solution to chemical information processing and searching [59]. It provides several utilities such as the generation of SMILES strings, graph-based substructure search, analyzing 2D and 3D structures of compounds and generation different types of fingerprints. Users can integrate their code using Java or C++.

***The Chemistry Development Kit (CDK)*** is an open source Java library that provides functions for generation and manipulation of various chemical formats such as SMILES and InChI, substructure searching using SMARTS, implementations of graph theory algorithms for chemical structure search and 3D structure generation [60].

***OpenEye Toolkit*** includes several options such as handling representations of several chemical formats, 2D structure-based shape similarity and clustering methods and 2D molecular structure rendering [61]. OpenEye toolkit is a proprietary product, however it provides a free Public Domain Research License for non-commercial research projects whose results are planned to be released publicly. OpenEye toolkit provides libraries for Python, C++, Java and C#.

***ChemmineR*** is an open source compound mining framework for statistical computing language and environment R [62]. ChemmineR provides various functionalities such as implementations of algorithms for handling different types of compound representations, 2D structural similarity searching, clustering algorithms for compound libraries, classification algorithms and visualization methods.

***Indigo*** is a free and open-source cheminformatics toolkit providing several utilities such as manipulation of SMILES strings, structure and substructure searching by SMARTS, generation of various types of fingerprints [63]. Indigo can be used with C/C++, C#, Java and Python programming languages.

## **Tools & Libraries for Target Proteins**

***PROFEAT (Protein Feature Server)*** is a web server that computes protein descriptors from input protein sequences based on commonly used structural and physicochemical features of amino acids. It also computes descriptors for ligands, protein structures, biological networks, protein-protein interactions and protein-ligand interactions. In total, it generates 11 feature groups for proteins including features AAC, DC, Autocorrelation (Normalized Moreau-Broto, Moran, Geary), CTD, SOCN & QSO descriptors, Pse-AAC, Am-Pse-AAC, topological descriptors and total amino acid properties. It can also calculate autocorrelations based on user defined properties. Moreover, it is possible to download results in GIST and CSV format in addition to printer-friendly view option [19]. The server is accessible at http://bidd2.nus.edu.sg/cgi-bin/profeat2016/protein/profnew.cgi.

***ProPy (Protein in Python)*** is a freely available and open source python package that computes commonly used protein descriptors from input sequences based on structural and physichochemical properties of amino acids. It generates 13 features including Sequence Composition (AAC, DC, TC), Autocorrelation (Normalized Moreau-Broto, Moran, Geary), C, T, D, SOCNs, QSO descriptors, Pse-AAC, and Am-Pse-AAC. It is possible to compute these descriptors based on user defined properties, which can be directly retrieved from AAindex database using the AAIndex module. The package is available at https://code.google.com/archive/p/protpy/downloads and can be run on Linux and MS-Windows [64].

***PyDPI (Drug−Protein Interaction with Python)*** is also a python package that calculates commonly used protein descriptors including 6 feature groups (Sequence Composition, Autocorrelation, CTD, CTriad, SOCNs & QSO descriptors, Pse-AAC I-II), molecular descriptors including 12 feature groups (Constitution, Topology, Connectivity, E-state, Kappa, Burden, Information, Autocorrelation, charge, Property, MOE-type, and fingerprints - topological fingerprints, MACCS keys, FP4 keys, E-state fingerprints, topological torsions, Morgan fingerprints - ), and protein-protein & ligand-protein interaction descriptors by combining different types of protein and ligand descriptors. It can also compute descriptors based on user-defined properties that can be retrieved from AAindex database [65]. It is freely available at https://sourceforge.net/projects/pydpicao/.

***protr*** is a freely available and open source R package that calculates various commonly used structural and physicochemical descriptors from protein sequences for the numerical representations of proteins. It generates 8 descriptor groups including Sequence Composition, Autocorrelation (Normalized Moreau-Broto, Moran, Geary), CTD, CTriad, SOCN & QSO descriptors, two types of Pse-AAC, scales-based descriptors derived by various dimensionality reduction methods, and profile-based features derived from PSSM. It allows to compute descriptors based on user-defined properties that are retrieved from AAindex database. It also computes similarity scores based on protein sequence alignment and GO semantic similarity measures. As a user-friendly web server form of protr, ProtrWeb is developed [66]. The protr package is available at http://cran.r-project.org/package=protr, and ProtrWeb server is accessible at http://protrweb.scbdd.com/#.

***Rcpi (Compound–Protein Interaction with R)*** is also a freely available R/Bioconductor package that calculates commonly used protein descriptors (same descriptors with protr) from input sequences. Additionally, it can calculate molecular descriptors based on constitutional, topological, geometrical, electronic, hybrid, and molecular properties, fingerprints, maximum common substructures of small molecules and protein-protein & compound-protein interaction descriptors [67]. The package is available at https://www.bioconductor.org/packages/release/bioc/html/Rcpi.html.

***camb (Chemically Aware Model Builder)*** is another open source R package that can be used for standardization of compounds, calculation of molecular and protein descriptors including 905 1D & 14 fingerprint type descriptors for small molecules, 8 types of amino acid descriptors (Z-scales, T-scales, ST-scales, VHSE, MSWHIM, FASGAI, ProtFP8, BLOSUM62 substitution matrix) and 12 whole protein sequence descriptors (Sequence Composition (AAC, DC, TC), Autocorrelation (Normalized Moreau-Broto, Moran, Geary), CTD, CTriad, SOCN, QSO, Pse-AAC, Am-Pse-AAC), feature selection, model training, generation of predictive models for bioactivity studies, and visualization & validation of results. It is coded in R, C++, Python and Java, and is available at https://github.com/cambDI/camb [68].

***ProFET (Protein Feature Engineering Toolkit)*** is a bioinformatics toolkit that generates various features from protein sequences based on different feature categories including biophysical quantitative properties, letter-based features, local potential features, information-based statistics, AA scale-based features, and transformed CTD features. It also provides the efficiently extraction of features that show high biological interpretability [69]. The source code can be freely downloaded from https://github.com/ddofer/ProFET.

***BLAST (Basic Local Alignment Search Tool)*** is one of the most popular sequence alignment tools that finds local similarities between nucleotide or protein sequences by comparing query sequence against user-defined sequences or against sequence databases. BLAST also offers a more sensitive algorithm for sequence similarity searches of proteins, which is PSI-BLAST [70]. As an iterative program for detection of similarities between distant sequences, PSI-BLAST constructs a position-specific score matrix from the multiple sequence alignment generated from BLAST output, and uses the score matrix as query to search the database. This process may be iterated many times to find significant similarities [71]. BLAST is a part of National Center for Biotechnology Information (NCBI) resources, and accessible online at https://blast.ncbi.nlm.nih.gov/Blast.cgi. It can be also downloaded from the same web page.

***ClustalW*** is a commonly used multiple sequence alignment tool that matches sequence similarities between three or more sequences by using guide trees. It is a highly sensitive progressive method that uses sequence weighting and position-specific gap penalties, and can be used for divergent protein sequences [72]. As the new version of ClustalW, Clustal Omega uses seeded guide trees and hidden Markov model (HMM) profiles to generate multiple sequence alignments and allows users to use existing alignments for the alignment of new sequences or to add new sequences to existing alignments. It also generates phylogenetic tree to see evolutionary relationships between sequences [73]. Clustal Omega is available online at https://www.ebi.ac.uk/Tools/msa/clustalo/, or can be downloaded from http://www.clustal.org/omega/.

***DALI*** is an online server that compares 3D structures of proteins based on distance matrices. It is one of the most commonly used structural alignment tools that serves over 20 years. It achieves pairwise comparisons and all-against-all multiple comparisons as well as comparisons against structures in PDB. It measures RMSD scores, but evaluate similarities based on Dali Z-scores where scores higher than 2 shows significant structural similarity [74,75]. It is accessible at http://ekhidna2.biocenter.helsinki.fi/dali/.

***MultiProt*** is an online structural alignment tool that can be used for the simultaneous alignment of multiple protein structures. It does not require the alignment of all the input molecules, instead it finds the common geometrical cores between them by detecting high scoring partial multiple alignments. The similarity measurement is based on RMSD scores, where lower scores mean higher similarities [76]. MultiProt is accessible at http://bioinfo3d.cs.tau.ac.il/MultiProt/, or can be downloaded from the same web page (for Linux only).

***TM-align*** is also a structural alignment tool that is used for the comparison of protein structures without using sequence order information. It combines dynamic programming iterations with TM-score rotation matrix. Higher TM-scores mean higher similarities [77]. It can be accessed and downloaded from https://zhanglab.ccmb.med.umich.edu/TM-align/.

***RCSB PDB Comparison Tool*** is a freely available online tool that calculates pairwise sequence alignments using blast2seq, Needleman-Wunsch, or Smith-Waterman algorithms and pairwise structure alignments using FATCAT, CE, Mammoth, TM-Align, or TopMatch algorithms. It involves pre-calculated structure comparisons of proteins retrieved from PDB, which is updated weekly [78]. It is available at http://www.rcsb.org/pdb/home/home.do#Category-analyze.

***SiteEngine*** is a structural similarity measurement tool that is developed for the prediction of potential binding sites of proteins based on the similarities of their geometrical and physicochemical properties with known binding sites. It is a quite fast tool that represents protein structures as physicochemically important surface points and uses a heuristic algorithm based on efficient geometric hashing and matching of triangles of physicochemical properties (including hydrogen-bond donor, hydrogen-bond acceptor, mixed donor/acceptor, hydrophobic aliphatic and aromatic(pi) contacts) between surface patches. It uses a hierarchical scoring scheme -including fast low-resolution scoring, overall surface scoring, and 1:1 correspondence score- to search for global and local similarities and generates a total score by combining these scores. It provides a Match score as a normalized version of total score to rank the prediction results and to determine the best superimposition. It also calculates RMSD values if overall fold of compared proteins are same [37]. It is freely available as a web server and downloadable as a stand-alone tool at <http://bioinfo3d.cs.tau.ac.il/SiteEngine/>.

***APoc (Alignment of Pockets)*** is a large-scale, sequence order-independent, structural alignment software package developed for the comparison of the experimentally-known or computationally predicted protein pockets. It uses an algorithm that generates initial alignments from gapless alignments and the alignments of secondary structures, fragments and local contact patterns. Then, the algorithm improves these initial alignments by iterative dynamic programming and iterative integer programming procedures to obtain an optimal alignment. The tool measures pocket similarity using PS-score and evaluates the statistical significance of this score. When compared with SiteEngine, APoc provides better performance [38]. APoc can be freely downloaded from <http://cssb.biology.gatech.edu/APoc>.

***eMatchSite*** is also a sequence order-independent local alignment tool used to align and compare ligand binding sites of computationally generated protein models. It extensively utilizes evolutionary information extracted from sequence profiles and entropy, and secondary structure profiles of weakly homologous templates in complex with ligands. It also captures physicochemical and structural properties of proteins and their interactions with ligands from hydrophobicity values of amino acids, 3D distributions of residues and binding probabilities of ligands. The tool generates a set of residue-level scores from this information and combines it with non-linear machine learning models for the estimation of distances between residues of aligned protein models. Local binding site alignments are constructed from these distances using Kuhn-Munkres algorithm. eMatchSite achieves high performance and it is highly tolerant to structural distortions in binding regions of protein models when compared with other tools such as SiteEngine [39]. It can be freely accessible for academic uses as an online server and downloadable as a software package at <http://brylinski.cct.lsu.edu/ematchsite>.

***G-LosA (Graph-based Local Structure Alignment)*** is a software package that is also used for the local alignment of protein structures independently from sequence order. It generates all possible alignments between two local structures by iterative maximum clique search and fragment superposition and determines the optimal alignment with the maximum GA-score (explained in Supp.1B). It is particularly successful in detecting local conserved regions on a protein surface regardless of the size and characteristics of the protein, which may be very helpful for computational drug discovery studies in terms of the prediction of ligand binding sites on proteins [40]. The package can be freely downloaded from <https://compbio.lehigh.edu/GLoSA/>.

***POSSUM (Position-specific scoring matrix-based feature generator for machine learning)*** is a freely available toolkit that generates 21 types of PSSM profile-based feature descriptors for proteins. It aims to facilitate the feature extraction for training and benchmarking of machine learning-based models [79]. It is accessible online at http://possum.erc.monash. edu/server.jsp, or can be downloaded from the same web page.

***GOSemSim*** is a freely available R package that provides quantitative comparison of semantic similarities for GO annotations of genes and gene products based on information content (IC) and graph based methods [80]. It is available at http://bioconductor.org/packages/2.6/bioc/html/GOSemSim.html.

***FragHMMent*** is a freely available bioinformatics tool that predicts residue-residue contacts in a protein sequence. It uses HMMs trained on homologous sequences, predicted secondary structure and local descriptors of protein structure [81]. The tool can be downloaded from http://predictioncenter.org/Services/FragHMMent/descr.html.

***PSIPRED*** is a web server that predicts secondary structures of proteins from their amino acid sequences. It incorporates two feed-forward neural networks that perform an analysis using the output generated from PSI-BLAST [82]. It is available at http://bioinf.cs.ucl.ac.uk/psipred/, or can be freely downloaded from the same web page.

***Naccess*** is a stand-alone program that calculates the accessible surface area of proteins and nucleic acids at atomic level based on Lee-Richards algorithm. It needs 3D atomic coordinates of molecules in PDB file format for calculation. The program is freely available for academy and non-profit institutions. It needs a Unix platform and a Fortran compiler to be installed and run [83]. It can be downloaded from http://wolf.bms.umist.ac.uk/naccess/.

***POPS (Parameter OPtimized Surfaces)*** is a freely available online tool that is used to calculate solvent accessible surface areas of proteins and nucleic acids at the atomic and residue level. It is based on a simple and easily derivable analytical formula, atomic and residue area parameters of which has been optimized against *Naccess* that is an accurate all-atom method. It can generate results from directly PDB ids of proteins or from their uploaded PDB files [84]. The server is available at https://mathbio.crick.ac.uk/wiki/POPS, and the source code can be downloaded from https://github.com/Fraternalilab/POPS.

***PocketPicker*** is a PyMOL plugin that predicts protein binding pockets with regard to buriedness of these potential binding sites through an automated grid-based method [85]. It can be downloaded from http://gecco.org.chemie.uni-frankfurt.de/pocketpicker/download.html.

***SCREEN (Surface Cavity REcognition and EvaluatioN)*** is an online tool that identifies and characterizes cavities of protein surfaces. It generates the set of cavities for each structure and measures the geometric and electrostatic attributes for each cavity [86]. The original Screen tool is replaced with Screen2, and it is available at https://bhapp.c2b2.columbia.edu/screen2/cgi-bin/screen2.cgi.

***trj_cavity*** is also a protein cavity analysis tool implemented within GROMACS framework. It is used to identify and characterize protein cavities detected within molecular dynamics (MD) trajectories [87]. It can be downloaded from https://sourceforge.net/projects/trjcavity/.

# DATABASES AND GOLD STANDARD DATASETS

## **Compound and Bioactivity Databases**

***PubChem*** (https://pubchem.ncbi.nlm.nih.gov) is a chemical structure knowledgebase to store information about compounds, their features and activities. PubChem was established in 2004 by the National Institute of Health (NIH). It is one of the largest chemical databases including ~230 million bioactivity data for ~320 million compounds and substances, which are mainly retrieved from high-throughput screening (HTS) experiments and updated daily. PubChem is a public database, which comprises of three inter-linked databases, namely, PubChem Substance, PubChem Compound, and PubChem BioAssay [88,89].

PubChem Substance (https://www.ncbi.nlm.nih.gov/pcsubstance) database includes the records for chemical substances, which is provided by individual contributors. Each deposited substance has a Substance ID under PubChem substance database. Different contributors may upload information about the same compound. Therefore, there could be more than one substance entry representing the same compounds in the Substance database.

PubChem Compound (https://www.ncbi.nlm.nih.gov/pccompound) database is created by combining all records representing the same substance into one unique compound entry. Compound database is constructed by applying an automated process (validation and normalization) on the substance database. Each compound entry has an associated compound ID and includes all the information provided by different contributors. It also provides cross-links to other databases and web servers for the related information about query compound.

PubChem BioAssay (https://www.ncbi.nlm.nih.gov/pcassay) database contains information about the bioactivities between compounds and targets, along with the descriptions of bioassay experiments. Bioassay entries are associated with a unique identifier called Assay ID.

PubChem also includes 3D structures of compounds (PubChem3D service) for the molecules that satisfy a list of criteria. PubChem offers various compound search options based on free text, identity, similarity, substructure, superstructure, molecular formula and 3D Conformer. The database includes their own molecular descriptors for the compounds (i.e. PubChem fingerprints).

***ChEMBL*** (https://www.ebi.ac.uk/chembl) is an open access large-scale compound and bioactivity database containing almost 15 million experimentally derived bioactivities, which was established as a resource of European Bioinformatics Institute -EMBL-EBI- [90]. ChEMBL includes compound records together with bio-activities in a structured form and it offers an advanced query interface for searching compounds based on various parameters. The most significant feature of ChEMBL is that the incorporated data is manually curated, meaning that experts are scanning the medicinal chemistry literature for relevant publications and record their findings to the database that covers not only binding affinity data, but also selectivity, efficacy, absorption, distribution, metabolism, excretion, toxicity (ADMET) properties and structure-activity relationship (SAR) data of compounds. This way, the data in ChEMBL is more reliable compared to other bioactivity resources. ChEMBL also incorporates the information available in other databases including deposited screening results from PubChem Bioassay and approved drugs from U.S. Food and Drug Administration (FDA) Orange Book [91] and DailyMed, and provides cross-references to many databases and web servers for the related information about the query compound or target. It also includes specialized databases for specific diseases such as Neglected Tropical Disease (NTD) archive and malaria.

In most of the other databases, the compounds are associated with single or multiple proteins as if the compound acted on these targets individually. However, this may not be the case always, since a compound may target a protein complex. Considering this issue, another feature that ChEMBL offers is that the targets are separated into groups as “Single Protein”, “Protein Family” and “Protein Complex” based on the curated assay results. Moreover, a confidence score ranging from 0-9 is assigned to activity data that reflects the specificity of both target and assay type. ChEMBL also offers several services such as similarity search tools, structure and substructure searches and a cross-reference tool for chemical identifiers (UniChem) for other EMBL-EBI resources.

***DrugBank*** database (http://www.drugbank.ca) is a comprehensive freely accessible resource of approved and experimental drugs along with their targets. It is manually curated by experts with high-quality standards [92]. Unlike PubChem and ChEMBL, DrugBank is a small-scale database including ~17,000 drug-target associations, but it covers almost all aspects of drugs containing information about their identification, pharmacology, interactions with foods and other drugs, clinical trials, pharmacoeconomics, experimental and predicted properties, spectra, and taxonomy as well as related targets, enzymes, and transporters. The information about target proteins includes amino acid sequence, structure and pathways. DrugBank also provides links to other chemical databases including KEGG, PubChem, ChEBI, PharmGKB, ChemSpider, BindingDB. Users can also find the UniProt and GenBank links for the targets. The drug entries in the DrugBank are called DrugCards. Each DrugCard contains detailed information about drug metabolism, ADMET properties and quantitative structure activity relationships (QSAR) information. As new information becomes available DrugCards are being updated and maintained by expert scientists.

***STITCH*** (http://stitch.embl.de/) is a database of experimental and predicted interactions between chemicals and target proteins [93]. It contains a huge amount of interactions (~1.6 billion) with the addition of predicted ones. The database incorporates experimentally validated interactions from ChEMBL, PDSP Ki Database and PDB, manually curated datasets of other databases, results from HTS experiments, and predicted interactions with confidence scores, which are obtained from text-mining of the literature. Users can query available interactions based on name of chemicals and proteins, chemical structures (SMILEs) and protein sequences. The webserver also provides graphical network representations of the interactions.

***BindingDB*** (www.bindingdb.org) is a freely accessible database that contains over 1 million experimentally measured binding affinities of protein-ligand interactions [94]. The primary resource of BindingDB database is scientific papers, patents and other biological and chemical databases. Users can search compounds or bioassay results based on different or combined search criteria such as chemical structures, target names and quantitative bioactivity values. Users can download 2D structures of compounds. BindingDB also provides three virtual screening methods. These virtual screening methods use Maximum Similarity, Binary Kernel Discrimination, and Support Vector Machines. Users can query their compounds to obtain predictions. One of the key features of BindingDB is that it also provides validation data sets for computational drug design studies (http://www.bindingdb.org/validation_sets/).

***Binding MOAD–Mother of All Databases*** (http://bindingmoad.org/) is a small-scale database of high-quality protein-ligand complexes and corresponding biological activity information, which contains around 25,000 protein-ligand interactions [95]. Differently from other bioactivity databases, Binding MOAD includes high-resolution 3D structures of proteins and their ligand annotations, which are obtained from PDB. It also classifies proteins according to family and enzymatic property information, and this classification is based on 90% sequence identity for removal of redundancy. The experimental bioactivity data (i.e. Kd, Ki and IC50 values) are obtained by performing literature search and it is updated in a semi-automated curation procedure every year.

***KEGG*** (Kyoto Encyclopedia of Genes and Genomes - http://www.kegg.jp/) is a knowledge base for understanding of biological systems from molecular-level information of genes and genomes that is extracted from large-scale datasets of genome sequencing or other high-throughput experimental techniques. KEGG comprises of several databases for molecular interaction networks, functional hierarchies of biological events, disease information and a compound-target gene database [96]. KEGG was first established in 1995 by the Human Genome Program of the Ministry of Education, Science, Sports and Culture in Japan. KEGG databases are divided into different categories, which are systems information, genomic information, chemical information and health information. A part of these resources is directly related to drug discovery and medicinal bioinformatics studies (i.e. DRUG, LIGAND and DISEASE databases).

The KEGG LIGAND database includes information about compounds within the cell, their functional information and enzymatic reactions. The KEGG LIGAND database is subdivided into five databases which are COMPOUND, GLYCAN, REACTION, RCLASS and ENZYME. KEGG BRITE database, which is under systems information category, consists of hierarchical text files (BRITE files), which hold information about biological events including drug-disease relationships and functional drug classification information. KEGG DRUG and DGROUP databases are under health information category. DRUG database can be used to obtain information for approved drugs, their chemical structure and the targets of associated drugs. DGROUP is a new database that is being developed with the aim to group drugs in terms of functional and structural relations. Information about diseases, their genetic and environmental relations and therapeutic effects of drugs on diseases are stored in the KEGG DISEASE database. KEGG also provides numerous tools for researchers; for example, SIMCOMP is used to carry out similarity searches between chemical compounds [97].

Considering the bioactivity databases, PubChem, ChEMBL, Binding MOAD and BindingDB include bioactivity information as well as activity measurements; while DrugBank, STITCH and KEGG includes only interaction information without any measurements.

***SIDER*** (Side Effect Resource - http://sideeffects.embl.de) is a drug database that mainly focuses on recorded side effects and indications of drugs. For a query drug, it outputs a table of side effects including their definitions, occurring frequencies and clickable labels for related chemicals and proteins as well as for selected side effects, and outputs a table of indications that also includes definitions and labels [98]. The originality of the database is about the retrieval of the content. It extracts the mentioned entities from public documents and prospectuses using a machine-readable system and Natural Language Processing step in addition to manual annotation. Chemical names are taken from the STICH database and targeted proteins are taken from the STRING database. Side effects can be downloaded from SIDER web service and the database provides reference to several chemical databases including PubChem.

***DCDB*** (Drug Combination Database - <http://www.cls.zju.edu.cn/dcdb/)> is a free database of drug combinations with the aim of helping the discovery of beneficial multi-component drugs for complex diseases [99]. DCDB includes over one thousand drug combinations comprising also the collections of unsuccessful ones, which are curated from more than 100,000 clinical studies and FDA Orange Book. It provides information about combined activities and indications, chemical, pharmacological properties and known targets of combined drugs.

***HMDB*** (The Human Metabolome Database - http://www.hmdb.ca/) is a manually curated, extensive database of human metabolites as a valuable source for metabolomics, biomarker discovery and clinical chemistry [100]. It currently contains over 110,000 metabolites and ~5,700 protein sequences associated to these metabolites. It has many different search options including text, sequence, structure search etc. One of the most useful aspects of the database is that it is also possible to search metabolites according to proteins they associated, reactions, pathways and diseases they involved. Each metabolite entry (“MetaboCard”) includes chemical, physical and biological properties, taxonomy, ontology, normal/abnormal concentrations, related enzymes, transporters, pathways, disease associations, SNP and mutation data as well as external links to other databases.

***T3DB*** (The Toxin and Toxin-Target Database - http://www.t3db.ca/) - current version is also known as “Toxic Exposome Database”- is a comprehensive toxin database including over 3,600 toxic substances, around 2,000 targets and more than 42,000 toxin-target associations [101]. With an interface similar to DrugBank and HMDB, it contains various valuable information for each toxin record (called “ToxCard”) involving their chemical, physical and biological properties, toxicity values, toxic effects, related targets, gene regulation, mechanisms, taxonomy, medical information etc. which are manually extracted from thousands of other sources. It has many search options such as advance search, text query, sequence, chemical structure, molecular weight searches and so on. It also provides external links to many other databases such as DrugBank, PubChem, KEGG, UniProt, STITCH etc. if available.

***ChemSpider*** (<http://www.chemspider.com/)> is an enormous resource of chemistry data that provides various types of properties and structural representations for compounds. It was originally developed as a hobby project for contribution to the chemistry community and first released in 2007. As acquired by Royal Society of Chemistry in 2009, it is now one of the most comprehensive chemical databases including over 60 million chemical structures from almost 500 data sources with a free and fast access [102]. ChemSpider describes their service as “Google for Chemistry” and “Wikipedia for chemists”. Users can search compounds by different types of chemical structure representations and textual format. ChemSpider provides various tools to convert a chemical structure from one representation to another (e.g. generation of a chemical structure from SMILES and vice versa). It aims to improve search and data quality by crowdsourced curation with expert review [3].

***ChEBI*** (Chemical Entities of Biological Interest - <https://www.ebi.ac.uk/chebi)> is a database of chemical entities that incorporates an ontological classification based on controlled vocabularies to describe properties of chemical structures and to specify relationships between them [103]. The database comprises ~55,000 entries, each of which is manually annotated and identified with a unique ChEBI identifier. In addition to chemical structures and ontological classifications, it also involves synonyms of entities such as their IUPAC (International Union of Pure and Applied Chemistry) names, chemical properties such as mass and charge, and provides cross-references to other chemical databases. ChEBI is a part of EMBL-EBI resources and linked with ChEMBL [4].

***ZINC*** (http://zinc15.docking.org/) is another free database of compounds, especially suitable for virtual screening studies [104]. It provides more than 100 million commercially-available compounds with 3D representations to be readily used for docking studies, which are modeled by using ChemAxon’ s JChem package and Omega application of OpenEye Scientific Software. ZINC provides special subsets of datasets such as worldwide commercially available approved drugs, or datasets of drug-like compounds. Various virtual screening benchmarking sets (i.e. gold-standard datasets) are constructed using data in ZINC. The ZINC web site provides search options based on name, structure, biological activity.

|  |  |
| --- | --- |

## **Target Protein Databases**

***AAindex*** is a database that represents various physicochemical and biochemical properties of amino acids in the form of numerical indices. It consists of three sections, which are AAindex1 for amino acid indices, AAindex2 for substitution matrices, and AAindex3 for amino acid pair-wise contact potentials. These indices are derived from published literature [105]. AAindex release 9.2 includes 566, 94, 47 entries for AAindex1, AAindex2, and AAindex3, respectively. The database can be accessed from http://www.genome.jp/aaindex/, or can be downloaded by anonymous FTP: ftp://ftp.genome.jp/pub/db/community/aaindex/.

***UniProt (Universal Protein Resource)*** is a comprehensive protein knowledgebase including sequence information and detailed functional annotation [106]. As a collaboration between the European Bioinformatics Institute (EBI), the Swiss Institute of Bioinformatics (SIB) and the Protein Information Resource (PIR), the UniProt consortium consists of four main data sources: UniProtKB, UniRef, UniParc, and Proteomes.

UniProtKB is the central resource that combines Swiss-Prot and TrEMBL. Swiss-Prot includes over 500,000 reviewed, non-redundant, high-quality protein sequences that have been manually curated and annotated by experts based on the experimental information extracted from the literature. Unlike Swiss-Prot, TrEMBL includes almost a hundred million computationally generated, unreviewed, redundant protein sequences that are mainly derived from high throughput sequencing data of DNA. TrEMBL uses rule based automatic annotation system to annotate these sequences.

UniRef enables over two hundred million clustered sets of sequences from UniProtKB and selected UniParc records by obtaining complete coverage of the sequence space at three levels (UniRef100, UniRef90, UniRef50) that helps to eliminate redundant sequences.

UniParc is a non-redundant protein sequence archive that provides a complete set of known sequences retrieved from publicly available sequence sources. It currently includes nearly two hundred million entries.

Proteomes provides expressed sets of proteins for each species whose genome is completely sequenced. It includes ~10,000 reference proteomes for well-studied model organisms and ~150,000 other proteomes.

In addition to these data sources, UniProt also provides four main tools: *BLAST* for sequence similarity search, *Align* for multiple sequence alignment, *Retrieve/ID mapping* for conversion of different identifiers into corresponding UniProt IDs and retrieval of them, and *Peptide search* to find exact matches against a query peptide sequence. Furthermore, UniProt provides cross-references to other data sources to supply more information with the related protein entry. UniProt is accessible at http://www.uniprot.org.

***InterPro*** is a comprehensive database and functional analysis tool that predicts protein domains and functional sites, and classifies them into families. This classification is achieved by the usage of predictive models (HMMs, profiles, PSSMs or regular expressions, collectively known as “signatures”) from several different databases that constitutes InterPro consortium as a powerful and integrated resource, which are *PROSITE, PRINTS, Pfam, ProDom, SMART, TIGRFAMs, PIRSF, SUPERFAMILY, PANTHER* and *Gene3D*. It also associates each entry with additional information such as description, consistent names and Gene Ontology (GO) terms [107]. As the most recent version (23.11.2017), InterPro 66.0 contains 32,568 entries. It is accessible at https://www.ebi.ac.uk/interpro/.

***Pfam*** is a database comprising a large collection of curated protein families, which are represented by multiple sequence alignments and HMMs as probabilistic models for the statistical inference of homology. By searching a protein sequence against the Pfam library, it is possible to identify its domain architecture and the family it belongs. It can be also used to analyze proteomes [108]. The most recent version (March 2017), Pfam 31.0 contains 16,712 protein families. The database is accessible at http://pfam.xfam.org/.

***PDB (The Protein Databank)*** is the single worldwide archive of the curated and annotated three-dimensional structural data of proteins, nucleic acids, and complex assemblies that is mainly obtained by X-Ray crystallography, but also includes NMR spectroscopy data and a small amount of electron microscopy data. It was founded in 1971 by Brookhaven National Laboratories (BNL) with seven crystal structures, and transferred to the Research Collaboratory for Structural Bioinformatics (RCSB) in 1998 [109]. The PDB archive is under responsible wwPDB (World Wide PDB - https://www.wwpdb.org/) organization that comprises PDBe (Europe - https://www.ebi.ac.uk/pdbe/), PDBj (Japan - https://pdbj.org/), and BMRB (Biological Magnetic Resonance Data Bank - http://www.bmrb.wisc.edu/) partners along with RCSB PDB (https://www.rcsb.org/). It is updated weekly and includes ~135,000 structures, currently.

***sc-PDB*** is an annotated protein structure database that comprises ligand binding sites of proteins and clusters these sites. These binding sites are defined from protein-ligand complexes in PDB. The database provides atom descriptions of the protein, its ligand, their binding site and binding mode [110]. Currently, it contains 16,034 binding sites from 4,782 proteins and 6,326 ligands. It is accessible at http://bioinfo-pharma.u-strasbg.fr/scPDB/.

***CATH (Class Architecture Topoloy Homologous superfamily)*** is a protein structure classification database based on an automated system that assigns domains retrieved from PDB to superfamilies using support vector machines. CATH hierarchy includes four levels for the assignment of domains related with the content, arrangement and connection of secondary structure elements, and evolutionary relationships; which are Class, Architecture, Topology, and Homologous superfamily, respectively [111]. The latest version (July 2017), CATH-Plus v4.2 contains over 95 million protein domains with predicted ones classified into 6,119 superfamilies. The database is accessible at http://www.cathdb.info/.

***SCOP (Structural Classification of Proteins)*** is a mainly manually curated database that also classifies protein structures based on a hierarchical level. The levels ‘fold’ and ‘class’ describe geometrical relationships related with certain packing arrangements of secondary structures and chain topologies while more specific levels including ‘family’ and ‘superfamily’ are determined in terms of evolutionary relationships of protein domains [112]. After SCOP 1.75 release, it was concluded and extended as SCOPe that includes automated curation methods and also incorporates the *ASTRAL* database [113]. As the latest version (February 2016), SCOPe 2.06 includes 244,326 domains classified into 2,008 superfamilies. The SCOP database is accessible at http://scop.mrc-lmb.cam.ac.uk/scop/, and SCOPe is accessible at https://scop.berkeley.edu/.

***ASTRAL (A Structural Alignment Library)*** compendium is a database that is used for the analysis of protein sequences and structures. It links protein sequences into related PDB structures and identifies domains within the sequence based on the classification in *SCOP(e)* database. It summarizes overall characteristics of protein structures according to Summary PDB ASTRAL Check Index (SPACI) scores and provides representative non-redundant subsets of classified protein structures and domains selected from high-quality structure files only [114]. It can be accessed from http://astral.berkeley.edu/. The new version of ASTRAL has been incorporated to SCOPe and its representative domain sequence subsets can be downloaded from https://scop.berkeley.edu/astral/ver=2.05.

## **Gold Standard Datasets for Virtual Screening**

Target protein families of most of the approved and experimental drugs, which also constitutes most of the gold-standard data sets:

- *G-protein coupled receptors (GPCRs)*, are one of the largest protein families including around 800 members in human that share common features in structure consisting an intracellular C-terminus and an extracellular N-terminus domain, and seven transmembrane alpha helices linked by three intracellular loops and three extracellular loops. They are involved in various signal transduction pathways, and targeted with the greater part of drugs in clinical usage [115].
- *Ion channels*, are membrane proteins with a porous structure that regulate the flow of ions across membranes. They are mainly divided into three groups as voltage-gated ion channels (e.g. Na, K, Ca), ligand-gated ion channels (e.g. glycine and IP3 receptors) and other ion channels such as aquaporins and connexins. They are the second largest group that is targeted with existing drugs after GPCRs [116].
- *Enzymes*, are protein catalysts that accelerate biochemical reactions for the conversion of substrates into products. They are classified into six main families based on the reactions they catalyze, which are oxidoreductases, transferases, hydrolases, lysases, isomerases and ligases, and identified with four level code enzyme commission (EC) numbers according to families they belong. Although the overall number of enzymes in the cell is much more than the receptors, the number of enzymes targeted by existing drugs is relatively small. The majority of enzyme targeting drugs show inhibitory effect on the target [117].
- *Nuclear receptors*, are a class of transcription factors that regulate the expression of genes by binding to related promoter regions in DNA. They have two main subclasses based on endogenous agonists; steroid and non-steroid hormone receptors [118].

# MACHINE LEARNING APPLICATIONS IN VIRTUAL SCREENING

## **Feature Selection**

One of the crucial steps in machine learning applications is selecting and reducing features to obtain best representative and compact feature set. Incorporating too many features for learning may decrease the performance due to the problems such as high dimensionality, correlation of features [119,120]. The number of candidate features to be used in virtual screening studies has been increasing as more information and experimental results about compounds and targets are available. In some of the computational drug discovery studies thousands; or even millions of features are employed [121,122]. Therefore, feature selection techniques became a necessity in drug discovery and bioinformatics studies. In this sense, the main objectives of feature selection are

- to increase classification performance by eliminating irrelevant and redundant features,
- to overcome the overfitting problem,
- to decrease the computational need and run time of algorithms,
- to provide better visualization and understanding of data and
- to reduce the data storage for large scale applications.

Various studies have been performed to analyze and compare feature reduction and selection techniques in the literature [119,123–127]. There are three main approaches of feature selection, which are filters, wrappers, and embedded approaches in machine learning area (Figure S.1).

***Filter methods*** select features by considering each feature individually and selecting more important and relevant features independent of a machine learning approach. In general, filter methods calculate a relevance score for each feature and decide the features to be selected. One of the main advantages of filter methods is that these methods are computationally efficient. Hence, filter methods can be applied to large-scale feature selection problems less computational power. In addition, filter methods are considered to be a preprocessing step for machine learning algorithms. These methods are run only once before training and the reduced feature set can then be used for the training. Filters are also used to solve overfitting problems. However, standard filter methods overlook the inter-feature dependencies, which may reduce prediction performance [128].

***Wrappers*** use subsets of an original feature set and determined feature subsets are used for training of the models [129]. There are different techniques that are used by the wrappers such as forward selection and backward elimination. In forward selection methods, features are added to an initially empty feature set one by one. Performance is evaluated after addition of new features and based on the results, the new feature is decided to be selected or not. Backward elimination is the opposite approach where the features are removed one by one and the same procedure applies for the rest. Both forward selection and backward elimination algorithms are greedy algorithms. One of the advantages of wrappers is that they consider inter-feature relationships by selecting different subsets of features each time. In addition, since the performance is evaluated after feature selection it is also possible to find best representative features specific to the applied machine learning method. One disadvantage of wrappers is the exponential increase in the number of subsets to be generated as the number of features increase. There are different heuristics that are used to overcome the search space problem of wrappers. Another disadvantage of wrappers is that they are computationally intensive methods and there is a risk of overfitting.


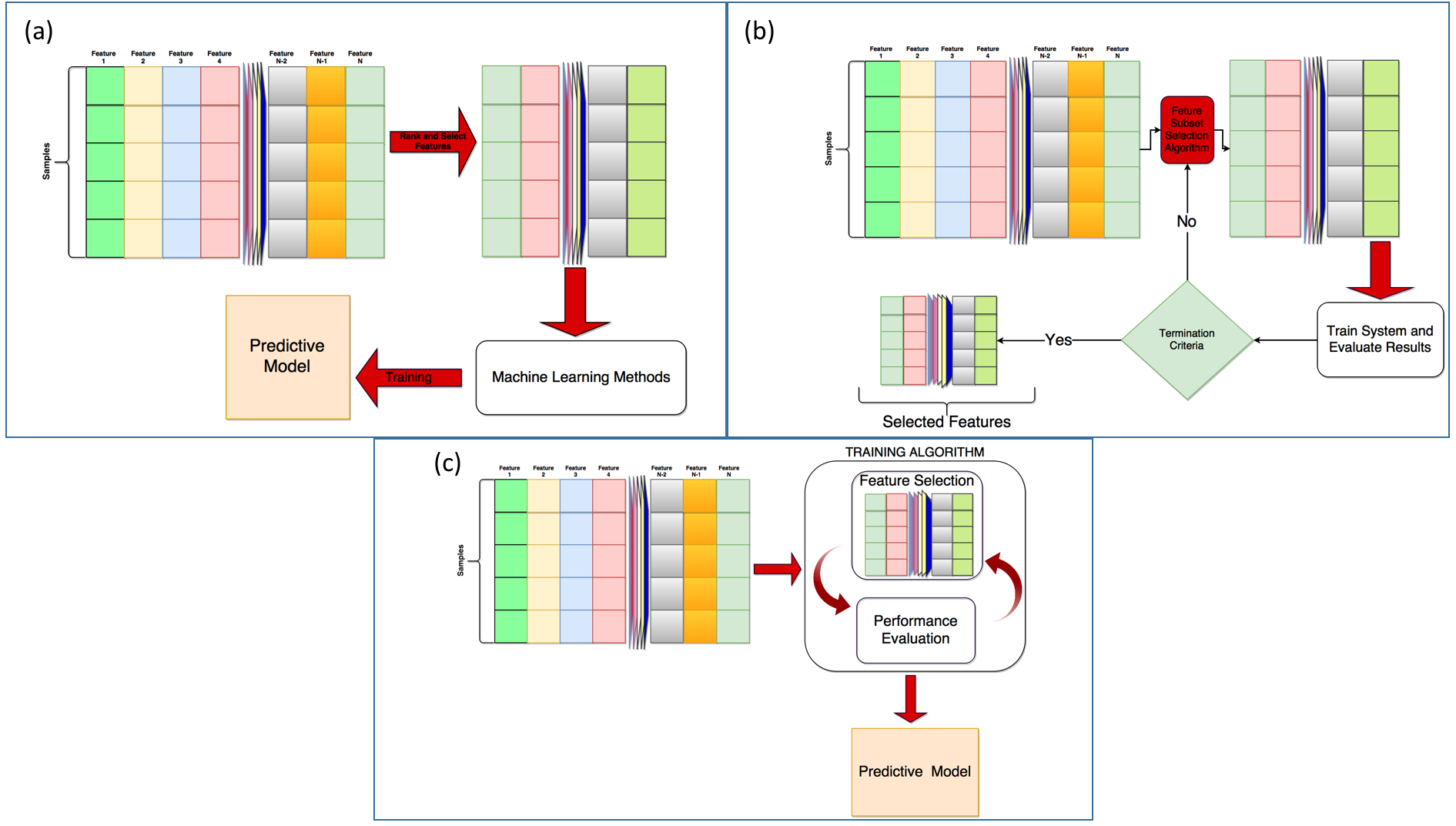


**Figure S.1.** Illustration of filter, wrapper and embedded models (a) Filter methods selects most representative features by evaluating them individually. (b) wrapper methods use different subsets of features and train the system with the selected features. This procedure continues until termination criteria is met. (c) Feature selection is the part of the machine learning methods in embedded methods.

***Embedded methods*** combine feature selection and learning steps [130]. Therefore, at the end not only the relevant features are selected but also a model is constructed. Embedded models are similar to the wrappers since they both select features specific to machine learning method to be used. One of the advantages of the embedded method is that it is not as computationally intensive as wrappers.

In recent years, the importance of feature selection methods has been emphasized in virtual screening studies and several feature selection methods have been applied [125,131–135]. Khan *et al.* investigated different types of molecular descriptors and virtual screening studies in terms of employed feature selection methods on molecular descriptors [134]. They showed, by several examples, that the applications of feature selection methods enhance the prediction results substantially in virtual screening studies. Filter methods, wrappers and embedded methods were investigated and applications of these feature selection methods in virtual screening were also demonstrated. Authors also stated that the generality and performance of the predictive models are highly dependent on the properties of the targets. Therefore, target-specific feature selection methods can be performed to enhance accuracy of prediction results. Saeys *et al*. reported feature selection is important for bioinformatics studies especially due to the following problems related to large input dimensionality and small sample sizes [125]. The authors stated ensemble future selection methods can be used to obtain more robust and representative features. Dahl *et al.* were also employed filter methods using information gain in their methods to compare the performance of their methods with using different feature sets [136].

##### **REFERENCES**

1. Weininger D. SMILES, a chemical language and information system. J. Chem. Inf. Comput. Sci. 1988; 28:31–36

2. SMARTS: A Language for Describing Molecular Patterns. http//www.daylight.com/dayhtml/doc/theory/theory.smarts.html, Accessed January 8, 2018

3. Heller SR, McNaught A, Pletnev I, et al. InChI, the IUPAC International Chemical Identifier. J. Cheminform. 2015; 7:1–34

4. Tutorial:Fingerprints -MACCS. https//openbabel.org/wiki/TutorialFingerprints, Accessed January 8, 2018.

5. Daylight Theory: Fingerprints. http//www.daylight.com/dayhtml/doc/theory/theory.finger.html, Accessed January 8, 2018

6. FP2 - Open Babel. https//openbabel.org/wiki/FP2, Accessed January 8, 2018

7. Rogers D, Hahn M. Extended-connectivity fingerprints. J. Chem. Inf. Model. 2010; 50:742–754

8. Salim N, Holliday J, Willett P. Combination of Fingerprint-Based Similarity Coefficients Using Data Fusion. J. Chem. Inf. Model. 2003; 43:435–442

9. Xu D, Tian Y. A Comprehensive Survey of Clustering Algorithms. Ann. Data Sci. 2015; 2:165–193

10. Li ZR, Lin HH, Han LY, et al. PROFEAT: a web server for computing structural and physicochemical features of proteins and peptides from amino acid sequence. Nucleic Acids Res. 2006; 34:W32–W37

11. Ong SA, Lin HH, Chen YZ, et al. Efficacy of different protein descriptors in predicting protein functional families. BMC Bioinformatics 2007; 8:300

12. Cao DS, Liu S, Xu QS, et al. Large-scale prediction of drug-target interactions using protein sequences and drug topological structures. Anal. Chim. Acta 2012; 752:1–10

13. Shen J, Zhang J, Luo X, et al. Predicting protein-protein interactions based only on sequences information. PNAS 2007; 104:4337–41

14. Schneider G, Wrede P. The Rational Design of Amino Acid Sequences by Artificial Neural Networks and Simulated Molecular Evolution: De Novo Design of an Idealized Leader Peptidase Cleavage Site. Biophys. J. 1994; 66:335–344

15. Chou K-C. Prediction of Protein Subcellular Locations by Incorporating Quasi-Sequence-Order Effect. Biochem. Biophys. Res. Commun. 2000; 278:477–483

16. Grantham R. Amino Acid Difference Formula to Help Explain Protein Evolution. Science. 1974; 185:862–864

17. Chen Y, Li W, Cai N. Predicting Protein Structural Class Based on Ensemble Binary Classification. 2009 Fifth Int. Conf. Nat. Comput. 2009; 167–170

18. Chou K-C. Prediction of protein cellular attributes using pseudo-amino acid composition. Proteins Struct. Funct. Genet. 2001; 44:60–60

19. Rao HB, Zhu F, Yang GB, et al. Update of PROFEAT: a web server for computing structural and physicochemical features of proteins and peptides from amino acid sequence. Nucleic Acids Res. 2011; 39:W385–W390

20. Chou K-C. Using amphiphilic pseudo amino acid composition to predict enzyme subfamily classes. Bioinformatics 2005; 21:10–19

21. Sandberg M, Eriksson L, Jonsson J, et al. New chemical descriptors relevant for the design of biologically active peptides. A multivariate characterization of 87 amino acids. J. Med. Chem. 1998; 41:2481–2491

22. Zaliani A, Gancia E. MS-WHIM Scores for Amino Acids: A New 3D-Decription for Peptide QSAR and QSPR Studies. J. Chem. Inf. Comput. Sci. 1999; 39:525–533

23. Mei H, Liao ZH, Zhou Y, et al. A new set of amino acid descriptors and its application in peptide QSARs. Biopolym. Pept. Sci. 2005; 80:775–786

24. Liang G, Li Z. Factor Analysis Scale of Generalized Amino Acid Information as the Source of a New Set of Descriptors for Elucidating the Structure and Activity Relationships of Cationic Antimicrobial Peptides. QSAR Comb. Sci. 2007; 26:754–763

25. van Westen GJP, Swier RF, Cortes-Ciriano I, et al. Benchmarking of protein descriptor sets in proteochemometric modeling (part 1): Modeling performance of 13 amino acid descriptor sets. J. Cheminform. 2013; 5:1

26. Henikoff S, Henikoff JG. Amino acid substitution matrices from protein blocks. Proc. Natl. Acad. Sci. 1992; 89:10915–10919

27. Dayhoff MO, Schwartz RM, Orcutt BC. A model of evolutionary change in proteins. Atlas protein Seq. Struct. 1978; 5 suppl. 3:345–351

28. Needleman SB, Wunsch CD. A general method applicable to the search for similarities in the amino acid sequence of two proteins. J. Mol. Biol. 1970; 48:443–453

29. Smith TF, Waterman MS. Identification of common molecular subsequences. J. Mol. Biol. 1981; 147:195–197

30. Gribskov M, McLachlan A, Eisenberg D. Profile analysis: detection of distantly related proteins. Proc. Natl. Acad. Sci. U. S. A. 1987; 84:4355–4358

31. Yu X, Zheng X, Liu T, et al. Predicting subcellular location of apoptosis proteins with pseudo amino acid composition: Approach from amino acid substitution matrix and auto covariance transformation. Amino Acids 2012; 42:1619–1625

32. Kufareva I, Abagyan R. Methods of protein structure comparison. Methods Mol. Biol. 2012; 857:231–57

33. Zemla A, Venclovas C, Moult J, et al. Processing and evaluation of predictions in CASP4. Proteins Struct. Funct. Genet. 2001; 45:13–21

34. Xu J, Zhang Y. How significant is a protein structure similarity with TM-score = 0.5? Bioinformatics 2010; 26:889–895

35. Siew N, Elofsson A, Rychlewski L, et al. MaxSub: An automated measure for the assessment of protein structure prediction quality. Bioinformatics 2000; 16:776–785

36. Zhang Y, Skolnick J. Scoring function for automated assessment of protein structure template quality. Proteins Struct. Funct. Bioinforma. 2004; 57:702–710

37. Shulman-Peleg A, Nussinov R, Wolfson HJ. Recognition of Functional Sites in Protein Structures. J. Mol. Biol. 2004; 339:607–633

38. Gao M, Skolnick J. APoc: large-scale identification of similar protein pockets. Bioinformatics 2013; 29:597–604

39. Brylinski M. eMatchSite: Sequence Order-Independent Structure Alignments of Ligand Binding Pockets in Protein Models. PLoS Comput Biol 2014; 10:

40. Lee HS, Im W. G-LoSA: An efficient computational tool for local structure-centric biological studies and drug design. Protein Soc. 2016; 25:865–876

41. Couto FM, Silva MJ, Coutinho PM. Measuring semantic similarity between Gene Ontology terms. Data Knowl. Eng. 2007; 61:137–152

42. Tian F, Zhou P, Li Z. T-scale as a novel vector of topological descriptors for amino acids and its application in QSARs of peptides. J. Mol. Struct. 2007; 830:106–115

43. Yang L, Shu M, Ma K, et al. ST-scale as a novel amino acid descriptor and its application in QSAM of peptides and analogues. Amino Acids 2010; 38:805–816

44. Hvidsten TR, Kryshtafovych A, Komorowski J, et al. A novel approach to fold recognition using sequence-derived properties from sets of structurally similar local fragments of proteins. Bioinformatics 2003; 19:ii81-91

45. Berkholz DS, Krenesky PB, Davidson JR, et al. Protein Geometry Database: a flexible engine to explore backbone conformations and their relationships to covalent geometry. Nucleic Acids Res. 2009; 38:D320–D325

46. Richardson JS. The Anatomy and Taxonomy of Protein Structure. Adv. Protein Chem. 1981; 167–339

47. Kurgan L, Disfani FM. Structural protein descriptors in 1-dimension and their sequence-based predictions. Curr Protein Pept Sci 2011; 12:470–89

48. Teilum K, Olsen JG, Kragelund BB. Functional aspects of protein flexibility. Cell. Mol. Life Sci. 2009; 66:2231–2247

49. Yamanishi Y, Pauwels E, Saigo H, et al. Extracting sets of chemical substructures and protein domains governing drug-target interactions. J. Chem. Inf. Model. 2011; 51:1183–1194

50. Stank A, Kokh DB, Fuller JC, et al. Protein Binding Pocket Dynamics. Acc. Chem. Res. 2016; 49:809–815

51. Ain QU, Méndez-Lucio O, Ciriano IC, et al. Modelling ligand selectivity of serine proteases using integrative proteochemometric approaches improves model performance and allows the multi-target dependent interpretation of features. Integr. Biol. 2014; 6:1023–1033

52. Weill N, Rognan D. Alignment-Free Ultra-High-Throughput Comparison of Druggable Protein−Ligand Binding Sites. J. Chem. Inf. Model. 2010; 50:123–135

53. Baroni M, Cruciani G, Sciabola S, et al. A common reference framework for analyzing/comparing proteins and ligands. Fingerprints for Ligands and Proteins (FLAP): Theory and application. J. Chem. Inf. Model. 2007; 47:279–294

54. RDKit: Open-Source Cheminformatics Software. http//www.rdkit.org/, Accessed January 8, 2018

55. O ’boyle NM, Banck M, James CA, et al. Open Babel: An open chemical toolbox. J. Cheminform. 2011; 3:1–14

56. Open Babel. http//openbabel.org/wiki/Main_Page, Accessed January 8, 2018

57. Mauri a, Consonni V, Pavan M, et al. Dragon software: An easy approach to molecular descriptor calculations. Match Commun. Math. Comput. Chem. 2006; 56:237–248

58. Tetko I V., Gasteiger J, Todeschini R, et al. Virtual Computational Chemistry Laboratory – Design and Description. J. Comput. Aided. Mol. Des. 2005; 19:453–463

59. Daylight Toolkit. http//www.daylight.com/products/toolkit.html, Accessed January 8, 2018

60. The Chemistry Development Kit. https//cdk.github.io/, Accessed January 8, 2018

61. OpenEye Toolkit. . https//docs.eyesopen.com/toolkits/python/index.html, Accessed January 8, 2018

62. Cao Y, Charisi A, Cheng LC, et al. ChemmineR: A compound mining framework for R. Bioinformatics 2008; 24:1733–1734

63. Indigo Toolkit. http//lifescience.opensource.epam.com/indigo/, Accessed January 8, 2018

64. Cao DS, Xu QS, Liang YZ. Propy: A tool to generate various modes of Chou’s PseAAC. Bioinformatics 2013; 29:960–962

65. Cao D-S, Liang Y-Z, Yan J, et al. PyDPI: Freely Available Python Package for Chemoinformatics, Bioinformatics, and Chemogenomics Studies. J. Chem. Inf. Model. 2013; 53:3086–3096

66. Xiao N, Cao D-S, Zhu M-F, et al. protr/ProtrWeb: R package and web server for generating various numerical representation schemes of protein sequences. Bioinformatics 2015; 31:1857–1859

67. Cao D-S, Xiao N, Xu Q-S, et al. Rcpi: R/Bioconductor package to generate various descriptors of proteins, compounds and their interactions. Bioinformatics 2015; 31:279–281

68. Murrell DS, Cortes-Ciriano I, Van Westen GJP, et al. Chemically Aware Model Builder (camb): An R package for property and bioactivity modelling of small molecules. J. Cheminform. 2015; 7:1–10

69. Ofer D, Linial M. ProFET: Feature engineering captures high-level protein functions. Bioinformatics 2015; 31:3429–3436

70. Boratyn GM, Camacho C, Cooper PS, et al. BLAST: a more efficient report with usability improvements. Nucleic Acids Res. 2013; 41:W29–W33

71. Schäffer AA, Aravind L, Madden TL, et al. Improving the accuracy of PSI-BLAST protein database searches with composition-based statistics and other refinements. Nucleic Acids Res. 2001; 29:2994–3005

72. Thompson JD, Higgins DG, Gibson TJ. CLUSTAL W: improving the sensitivity of progressive multiple sequence alignment through sequence weighting, position-specific gap penalties and weight matrix choice. Nucleic Acids Res. 1994; 22:4673–4680

73. Sievers F, Wilm A, Dineen D, et al. Fast, scalable generation of high-quality protein multiple sequence alignments using Clustal Omega. Mol. Syst. Biol. 2011; 7:1–6

74. Holm L, Sander C. Protein Structure Comparison by Alignment of Distance Matrices. J. Mol. Biol. 1993; 233:123–138

75. Holm L, Laakso LM. Dali server update. Nucleic Acids Res. 2016; 44:W351-5

76. Shatsky M, Nussinov R, Wolfson HJ. A method for simultaneous alignment of multiple protein structures. Proteins Struct. Funct. Bioinforma. 2004; 56:143–156

77. Zhang Y, Skolnick J. TM-align: a protein structure alignment algorithm based on the TM-score. Nucleic Acids Res. 2005; 33:2302–2309

78. Prlic A, Bliven S, Rose PW, et al. Pre-calculated protein structure alignments at the RCSB PDB website. Bioinforma. Appl. NOTE 2010; 26:2983–298510

79. Wang J, Yang B, Revote J, et al. POSSUM: a bioinformatics toolkit for generating numerical sequence feature descriptors based on PSSM profiles. Bioinformatics 2017; 33:2756–2758

80. Yu G, Li F, Qin Y, et al. GOSemSim: an R package for measuring semantic similarity among GO terms and gene products. Bioinforma. Appl. NOTE 2010; 26:976–97810

81. Björkholm P, Daniluk P, Kryshtafovych A, et al. Using multi-data hidden Markov models trained on local neighborhoods of protein structure to predict residue–residue contacts. Bioinformatics 2009; 25:1264–1270

82. McGuffin LJ, Bryson K, Jones DT. The PSIPRED protein structure prediction server. Bioinformatics 2000; 16:404–405

83. Hubbard SJ, Thornton JM. ‘NACCESS’, computer program. Dep. Biochem. Mol. Biol. Univ. Coll. London. 1993;

84. Cavallo L, Kleinjung J, Fraternali F. POPS: a fast algorithm for solvent accessible surface areas at atomic and residue level. Nucleic Acids Res. 2003; 31:3364–3366

85. Weisel M, Proschak E, Schneider G. PocketPicker: analysis of ligand binding-sites with shape descriptors. Chem. Cent. J. 2007; 1:1–17

86. Nayal M, Honig B. On the nature of cavities on protein surfaces: Application to the identification of drug-binding sites. Proteins Struct. Funct. Bioinforma. 2006; 63:892–906

87. Paramo T, East A, Garzón D, et al. Efficient characterization of protein cavities within molecular simulation trajectories: trj-cavity. J. Chem. Theory Comput. 2014; 10:2151–2164

88. Wang Y, Suzek T, Zhang J, et al. PubChem BioAssay: 2014 update. Nucleic Acids Res. 2014; 42:1075–1082

89. Kim S, Thiessen PA, Bolton EE, et al. PubChem Substance and Compound databases. Nucleic Acids Res. 2016; 44:D1202-13

90. Bento AP, Gaulton A, Hersey A, et al. The ChEMBL bioactivity database: An update. Nucleic Acids Res. 2014; 42:1083–1090

91. OrangeBook. https//www.fda.gov/Drugs/InformationOnDrugs/ucm129662.htm, Accessed January 8, 2018

92. Law V, Knox C, Djoumbou Y, et al. DrugBank 4.0: Shedding new light on drug metabolism. Nucleic Acids Res. 2014; 42:1091–1097

93. Kuhn M, Szklarczyk D, Pletscher-Frankild S, et al. STITCH 4: Integration of protein-chemical interactions with user data. Nucleic Acids Res. 2014; 42:401–407

94. Gilson MK, Liu T, Baitaluk M, et al. BindingDB in 2015: A public database for medicinal chemistry, computational chemistry and systems pharmacology. Nucleic Acids Res. 2016; 44:D1045–D1053

95. Ahmed A, Smith RD, Clark JJ, et al. Recent improvements to Binding MOAD: A resource for protein-ligand Binding affinities and structures. Nucleic Acids Res. 2015; 43:D465–D469

96. Kanehisa M, Sato Y, Kawashima M, et al. KEGG as a reference resource for gene and protein annotation. Nucleic Acids Res. 2016; 44:D457–D462

97. Hattori M, Tanaka N, Kanehisa M, et al. SIMCOMP/SUBCOMP: Chemical structure search servers for network analyses. Nucleic Acids Res. 2010; 38:1–5

98. Kuhn M, Letunic I, Jensen LJ, et al. The SIDER database of drugs and side effects. Nucleic Acids Res. 2016; 44:D1075-9

99. Liu Y, Wei Q, Yu G, et al. DCDB 2.0: a major update of the drug combination database. Database (Oxford). 2014; 2014:1–6

100. Wishart DS, Feunang YD, Marcu A, et al. HMDB 4.0: the human metabolome database for 2018. Nucleic Acids Res. 2018; 46:D608–D617

101. Wishart D, Arndt D, Pon A, et al. T3DB: the toxic exposome database. Nucleic Acids Res. 2015; 43:D928-34

102. Williams A, Tkachenko V. The Royal Society of Chemistry and the delivery of chemistry data repositories for the community. J. Comput. Aided. Mol. Des. 2014; 1023–1030

103. Hastings J, De Matos P, Dekker A, et al. The ChEBI reference database and ontology for biologically relevant chemistry: Enhancements for 2013. Nucleic Acids Res. 2013; 41:456–463

104. Sterling T, Irwin JJ. ZINC 15 - Ligand Discovery for Everyone. J. Chem. Inf. Model. 2015; 55:2324–2337

105. Kawashima S, Pokarowski P, Pokarowska M, et al. AAindex: amino acid index database, progress report 2008. Nucleic Acids Res. 2008; 36:D202–D205

106. The UniProt Consortium. UniProt: the universal protein knowledgebase. Nucleic Acids Res. 2017; 45:D158–D169

107. Finn RD, Attwood TK, Babbitt PC, et al. InterPro in 2017––beyond protein family and domain annotations. Nucleic Acids Res. 2017; 45:D190–D199

108. Finn RD, Coggill P, Eberhardt RY, et al. The Pfam protein families database: towards a more sustainable future. Nucleic Acids Res. 2016; 44:D279–D285

109. Berman HM, Westbrook J, Feng Z, et al. The Protein Data Bank. Nucleic Acids Res. 2000; 28:235–242

110. Desaphy J, Bret G, Rognan D, et al. sc-PDB: a 3D-database of ligandable binding sites––10 years on. Nucleic Acids Res. 2015; 43:D399–D404

111. Dawson NL, Lewis TE, Das S, et al. CATH: an expanded resource to predict protein function through structure and sequence. Nucleic Acids Res. 2017; 45:D289–D295

112. Conte L Lo, Ailey B, Hubbard TJP, et al. SCOP: a Structural Classification of Proteins database. Nucleic Acids Res. 2000; 28:257–259

113. Fox NK, Brenner SE, Chandonia J-M. SCOPe: Structural Classification of Proteins—extended, integrating SCOP and ASTRAL data and classification of new structures. Nucleic Acids Res. 2014; 42:D304–D309

114. Brenner SE, Koehl P, Levitt M. The ASTRAL compendium for protein structure and sequence analysis. Nucleic Acids Res. 2000; 28:254–256

115. Alexander SP, Davenport AP, Kelly E, et al. The Concise Guide to PHARMACOLOGY 2015/16: G protein-coupled receptors. Br. J. Pharmacol. 2015; 172:5744–5869

116. Alexander SP, Kelly E, Marrion N, et al. The Concise Guide to PHARMACOLOGY 2015/16: Overview. Br. J. Pharmacol. 2015; 172:5729–5743

117. Alexander SP, Fabbro D, Kelly E, et al. The Concise Guide to PHARMACOLOGY 2015/16: Enzymes. Br. J. Pharmacol. 2015; 172:6024–6109

118. Alexander SP, Cidlowski JA, Kelly E, et al. The Concise Guide to PHARMACOLOGY 2015/16: Nuclear hormone receptors. Br. J. Pharmacol. 2015; 172:5956–5978

119. Hall M. Correlation-based Feature Selection for Machine Learning. PhD thesis 1999; 21i195-i20:1–5

120. Powell WB, Wiley InterScience (Online service). Approximate dynamic programming : solving the curses of dimensionality. Wiley Intersci. 2011; 627

121. Sawada R, Kotera M, Yamanishi Y. Benchmarking a wide range of chemical descriptors for drug-target interaction prediction using a chemogenomic approach. Mol. Inform. 2014; 33:719–731

122. Guyon I, Elisseeff A. An Introduction to Variable and Feature Selection. J. Mach. Learn. Res. 2003; 3:1157–1182

123. Padmaja DL, Vishnuvardhan B. Comparative Study of Feature Subset Selection Methods for Dimensionality Reduction on Scientific Data. 2016 IEEE 6th Int. Conf. Adv. Comput. 2016; 31–34

124. Janecek A, Gansterer WNW, Demel M, et al. On the Relationship Between Feature Selection and Classification Accuracy. Fsdm 2008; 4:90–105

125. Saeys Y, Inza I, Larrañaga P. A review of feature selection techniques in bioinformatics. Bioinformatics 2007; 23:2507–2517

126. Liu Y. A comparative study on feature selection methods for drug discovery. J. Chem. Inf. Comput. Sci. 2004; 44:1823–1828

127. Fodor IK. A survey of dimension reduction techniques. Library (Lond). 2002; 18:1–18

128. John G, Kohavi R, Pfleger K. Irrelevant Features and the Subset Selection Problem. Mach. Learn. Proc. 1994; 121–129

129. Kohavi R, John GH. Wrappers for feature subset selection. Artif. Intell. 1997; 97:273–324

130. Lal TN, Chapelle O, Weston J, et al. Embedded Methods. Stud. Fuzziness Soft Comput. 2006; 207:137–165

131. Liu L, Chen L, Zhang Y-H, et al. Analysis and prediction of drug-drug interaction by minimum redundancy maximum relevance and incremental feature selection. J. Biomol. Struct. Dyn. 2016; 1102:1–452

132. Korkmaz S, Zararsiz G, Goksuluk D. Drug/nondrug classification using Support Vector Machines with various feature selection strategies. Comput. Methods Programs Biomed. 2014; 117:51–60

133. Johansson U, Sonstrod C, Norinder U, et al. Using Feature Selection with Bagging and Rule Extraction in Drug Discovery. Adv. Intell. Decis. Technol. 2010; 413–422

134. Khan AU. Descriptors and their selection methods in QSAR analysis: paradigm for drug design. Drug Discov. Today 2016; 21:1291–1302

135. Liu Z, Guo F, Gu J, et al. Similarity-based prediction for Anatomical Therapeutic Chemical classification of drugs by integrating multiple data sources. Bioinformatics 2015; 31:1788–1795

136. Dahl, George E; Jaitly, Navdeep; Salakhutdinov R. Multi-task Neural Networks for QSAR Predictions. arXiv 2014; 1–21
